# Asparagine endopeptidase cleaves apolipoprotein A1 and accelerates pathogenesis of atherosclerosis

**DOI:** 10.1172/JCI185128

**Published:** 2025-05-15

**Authors:** Mengmeng Wang, Bowei Li, Shuke Nie, Xin Meng, Guangxing Wang, Menghan Yang, Wenxin Dang, Kangning He, Tucheng Sun, Ping Xu, Xifei Yang, Keqiang Ye

**Affiliations:** 1 Brain Cognition and Brain Disease Institute, Shenzhen Institutes of Advanced Technology, Chinese Academy of Sciences, Shenzhen, Guangdong, China.; 2 Faculty of Life and Health Sciences, Shenzhen University of Advanced Technology (SUAT), University of Chinese Academy of Science, Shenzhen, Guangdong, China.; 3 Department of Neurology, Renmin Hospital of Wuhan University, Wuhan, Hubei Province, China.; 4 Department of Laboratory Medicine, Sichuan Provincial People’s Hospital, University of Electronic Science and Technology of China, Chengdu, Sichuan, China.; 5 Zhejiang University-University of Edinburgh Institute (ZJU-UoE Institute), Zhejiang University School of Medicine, International Campus, Zhejiang University, Haining, China.; 6 Department of Cardiac Surgery, Guangdong Cardiovascular Institute, Guangdong Provincial Key Laboratory of South China Structural Heart Disease, Guangdong Provincial People’s Hospital, Guangdong Academy of Medical Sciences, Guangzhou, China.; 7 State Key Laboratory of Proteomics, Beijing Proteome Research Center, National Center for Protein Sciences (Beijing), Research Unit of Proteomics & Research and Development of New Drug of Chinese Academy of Medical Sciences, Institute of Lifeomics, Beijing, China.; 8 Key Laboratory of Modern Toxicology of Shenzhen, Shenzhen Medical Key Discipline of Health Toxicology (2020–2024), Shenzhen Center for Disease Control and Prevention, Shenzhen, Guangdong, China.

**Keywords:** Cardiology, Vascular biology, Atherosclerosis, Cholesterol, Macrophages

## Abstract

Atherosclerosis is a slowly progressing inflammatory disease characterized with cholesterol disorder and intimal plaques. Asparagine endopeptidase (AEP) is an endolysosomal protease that is activated under acidic conditions and is elevated substantially in both plasma and plaques of patients with atherosclerosis. However, how AEP accelerates atherosclerosis development remains incompletely understood, especially from the view of cholesterol metabolism. This project aims to reveal the crucial substrate of AEP during atherosclerosis plaque formation and to lay the foundation for developing novel therapeutic agents for Atherosclerosis. Here, we show that AEP is augmented in the atherosclerosis plaques obtained from patients and proteolytically cuts apolipoprotein A1 (APOA1) and impairs cholesterol efflux and high-density lipoprotein (HDL) formation, facilitating atherosclerosis pathologies. AEP is activated in the liver and aorta of apolipoprotein E–null (APOE-null) mice, and deletion of AEP from APOE^–/–^ mice attenuates atherosclerosis. APOA1, an essential lipoprotein in HDL for cholesterol efflux, is cleaved by AEP at N208 residue in the liver and atherosclerotic macrophages of APOE^–/–^ mice. Blockade of APOA1 cleavage by AEP via N208A mutation or its specific inhibitor, #11a, substantially diminishes atherosclerosis in both APOE^–/–^ and LDLR^–/–^ mice. Hence, our findings support that AEP disrupts cholesterol metabolism and accelerates the development of atherosclerosis.

## Abstract 2

In the current work, we show that AEP cleaves APOA1, a major component in HDL, diminishes cholesterol clearance, facilitating atherosclerosis onset.

## Graphical abstract

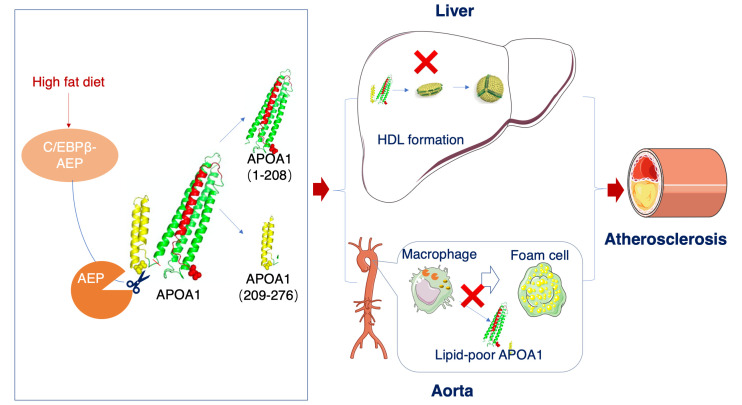

## Introduction

Atherosclerosis, the most prevalent form of cardiovascular disease, is characterized by lipid deposition in arterial walls. Chronic inflammation of large arteries serves as a primary pathological mechanism underlying cardiovascular disorders and cerebrovascular events such as stroke (1). A defining characteristic of atherosclerosis is the presence of cholesterol-laden plaques within arterial walls and dysregulated cholesterol profiles in the bloodstream, notably elevated levels of low-density lipoprotein (LDL) cholesterol and lipoproteins, coupled with reduced concentrations of high-density lipoprotein (HDL) cholesterol. Asparagine endopeptidase (AEP), alternatively referred to as legumain (*LGMN*), is a cysteine protease activated under acidic conditions and belongs to the C13 peptidase family. Predominantly localized within the endolysosomal system, this enzyme has recently emerged as a novel biomarker associated with atherosclerosis development (2). AEP is widely distributed, mainly in the kidneys and testis. As an enzyme, AEP specifically cleaves its substrates after asparagine residues and is mainly localized to the endolysosomal system; however, under pathophysiologic conditions, AEP also locates in the cytoplasm or in the nucleus (3, 4). However, the effect of AEP on cholesterol metabolism has rarely been reported.

AEP expression increases substantially in arteries of apolipoprotein E (APOE)^–/–^ mice with atherogenic plaques and human atherosclerotic lesions (5). As a protease, previous research on AEP and atherosclerosis has focused on its degradation of the extracellular matrix, which led to increased plaque instability and chance of rupture (6). Recently, it has been reported that, not only in the plaques, the levels of AEP also increase markedly in the plasma of patients with carotid atherosclerosis compared with healthy controls, and patients with recent symptoms have increased expression of AEP compared with patients who are asymptomatic (7). Interestingly, atorvastatin (referred to also as “statin”) substantially reduces the expression of AEP in monocytes from patients with atherosclerosis (8) and in macrophages in vitro (9). In addition, atherogenic lipids, especially cholesterol crystal, increase AEP secretion (7), underscoring the interplay between macrophages and cholesterol in the secretion of AEP.

C/EBP-β is a transcription factor with well-established roles in the transcriptional and translational regulation of lipid metabolism in hepatic and adipose tissues (10, 11). Our research reveals that C/EBP-β serves as a primary regulator of AEP transcription during aging processes (12). Specific deletion of C/EBP-β in hematopoietic cells of APOE^–/–^ mice demonstrates marked reductions in total cholesterol and LDL-cholesterol levels (13, 14). Furthermore, genetic silencing of C/EBP-β in RAW264.7 macrophage cells effectively inhibits oxLDL-induced foam cell formation while enhancing cellular cholesterol efflux capacity (13), suggesting that C/EBP-β plays a key role in cholesterol metabolism. Atherosclerosis is an age-dependent inflammatory disease (15) associated with infiltrated macrophages and vascular pathology. C/EBP-β and AEP are intimately implicated in atherosclerosis (12, 16–18). Recently, we show that the C/EBP-β/AEP pathway mediates atherosclerosis pathology (19).

Apolipoprotein A1 (APOA1), the primary protein component of HDLs, is a 243 amino–acid polypeptide that serves as an essential cofactor for lecithin-cholesterol acyltransferase (LCAT). This enzyme catalyzes the synthesis of nearly all plasma cholesteryl esters, a critical process in lipid metabolism (20). The transfer of cholesterol and phospholipids from macrophages to APOA1 plays a cardioprotective role through the reverse cholesterol transport (RCT) pathway. This process facilitates the transport of cholesterol to the liver, where it is excreted as a component of bile (21). The efflux of cholesterol and phospholipids to APOA1 leads to the formation of nascent, discoidal HDL particles, which are quickly transformed by LCAT into the spherical HDL particles present in plasma. Plasma HDL-C levels are reportedly inversely associated with cardiovascular risk, with HDL believed to offer cardiovascular protection by modulating the RCT process (22). However, clinical trials focused on increasing HDL levels have sparked considerable debate over the validity of the HDL hypothesis (23, 24). Administration of purified APOA1 to mice, as well as induction of human APOA1 gene overexpression through transgenic or adenoviral approaches, has been shown to attenuate atherosclerosis. These findings demonstrate that APOA1 exerts antiatherogenic effects by modulating multiple pathways, including reduction of plasma lipid levels, decreased macrophage accumulation, suppression of inflammatory responses, and inhibition of immune cell retention within atherosclerotic lesions (25–27). However, several clinical trials reported that APOA1 infusion into patients with acute coronary syndrome (ACS) did not show positive outcomes (28). Posttranslational modification or degradation of APOA1 might contribute to the failure of the clinical trials (29).

The vast majority of APOA1 within atherosclerotic human arterial tissue is lipid poor and does not reside on an HDL-like particle, which is in contrast with the APOA1 within circulation. The concentration of APOA1 in atherosclerotic lesions exceeds that found in the normal arterial wall by more than 100 times. Markedly, the functional analysis of APOA1 quantitatively retrieved from the aorta reveals approximately 80% reduced cholesterol efflux activity and about 90% diminished LCAT activity compared with circulating APOA1 (30). Sequencing of protein from human carotid atherosclerotic plaques showed APOA1 peptides ending with N, suggesting that APOA1 at plaques might be cleaved by AEP (31). Hence, we hypothesize that elevated AEP might cut APOA1 and disrupt cholesterol metabolism, accelerating the development of lesions. In this study, we explore the pathological functions of AEP in the development of atherosclerosis using APOE^–/–^ and LDLR^–/–^ mouse models and assess the therapeutic potential of its specific inhibitor.

## Results

### AEP activation is increased in the liver and aorta of APOE^–/–^ mice.

**Figure 1:**
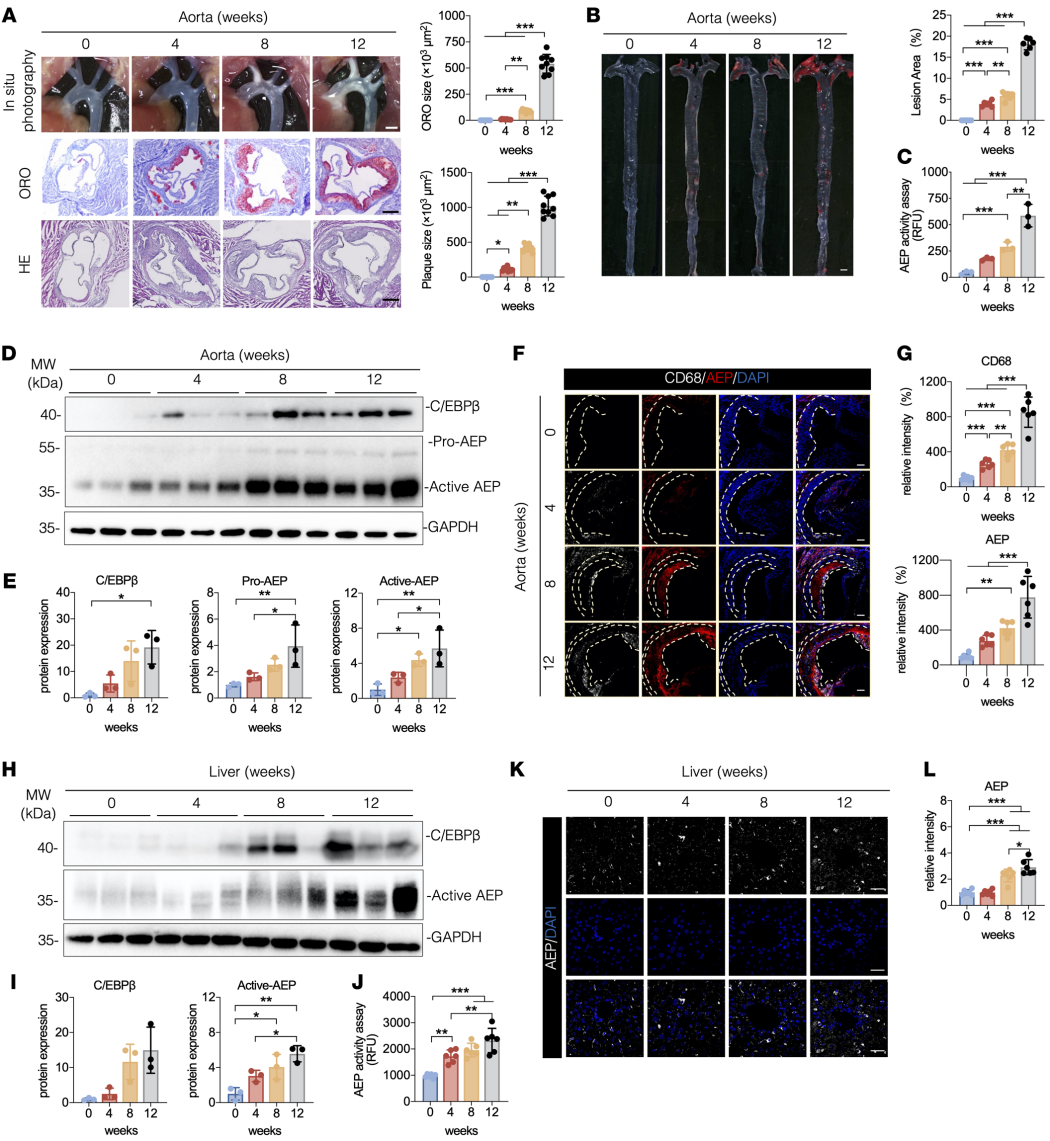
Increased AEP activation in the liver and aorta of APOE^–/–^ mice. (**A**–**L**) APOE^–/–^ mice were fed with a HFD for 0–12 weeks. (**A**) Representative macroscopic images and quantification of aortic arch and aortic root stained with H&E and ORO (*n* = 9 per group). Scale bars: 1 mm (top); 25 μm (bottom 2 rows). (**B**) Representative macrographs and quantification of aorta stained with ORO (*n* = 6 per group). Scale bar: 1 mm. (**C**) AEP enzymatic activities of aorta (*n* = 3 per group). (**D** and **E**) Western blot images and quantification of C/EBP-β and AEP levels in aorta (*n* = 3 per group). (**F** and **G**) IF staining and quantification of CD68 (green) and AEP (red) in aorta. Nuclei were counter-stained with DAPI (blue). Scale bar: 20 μm. (**H** and **I**) Western blot images and quantification of C/EBP-β and AEP levels in liver (*n* = 3 per group). (**J**) AEP enzymatic activities of liver (*n* = 6 per group). (**K** and **L**) IF staining and quantification of AEP (white) in liver. Nuclei were counter stained with DAPI (blue). Scale bar: 20 μm. All data are presented as the mean ± SEM from 3 to 6 independent experiments. 1-way ANOVA with Tukey’s post hoc test (**A**–**C**, **E**, **G**, **I**, **K**, and **L**); 1-way ANOVA with Kruskal–Wallis test (C/EBP-β in **I** and **L**). *P < 0.05; **P < 0.01; ***P < 0.001.

To investigate whether AEP is implicated in atherosclerosis onset, we employed APOE^–/–^ mice fed with a high-fat diet (HFD) (Clinton/Cybulsky Rodent Diet D12108 with 1.25% cholesterol) for different time points. The aortic arch displayed time-dependent fat deposition in the bifurcation of the artery. Cross section analysis revealed the lipid accumulation on the wall of the artery, which was validated by Oil-Red O (ORO) and H&E staining. The ORO size and plaques size were quantified and demonstrated progressive escalation (Figure 1A). Whole aorta (en face measurement) with ORO staining showed that lesion areas in APOE^–/–^ mice were gradually augmented, as HFD treatment was progressively elongated (Figure 1B). AEP enzymatic activities in the atherosclerotic area were increased in a time-dependent manner (Figure 1C). Immunoblotting (IB) revealed that C/EBP-β, a major upstream transcription factor for *LGMN*, was also enhanced with time, so was the downstream target AEP. The 37 kDa active form tightly correlated with the upstream effector (Figure 1, D and E). Immunofluorescent (IF) costaining with aorta sections using anti-CD68, a specific macrophage marker, and anti-AEP demonstrated that AEP levels were steadily augmented as more and more macrophages accumulated in the atherosclerotic plaques (Figure 1, F and G). IB analysis with liver lysates disclosed the similar increasing escalation patterns for both C/EBP-β and active AEP (Figure 1, H and I). AEP IF staining on the liver sections from APOE^–/–^ mice also exhibited the similar pattern (Figure 1, K and L), which was validated by AEP enzymatic assays (Figure 1J). IF results indicated that AEP was primarily expressed in cells with high CD68 expression in the liver and aorta (Supplemental Figure 1, A and B; supplemental material available online with this article; https://doi.org/10.1172/JCI185128DS1). The expression and activity of AEP in hepatic macrophages were substantially increased as the mice aged. Thus, AEP is gradually activated in the atherosclerotic plaques and liver from APOE^–/–^ mice fed with a HFD.

### Depletion of AEP from APOE^–/–^ mice attenuates atherosclerosis.

**Figure 2:**
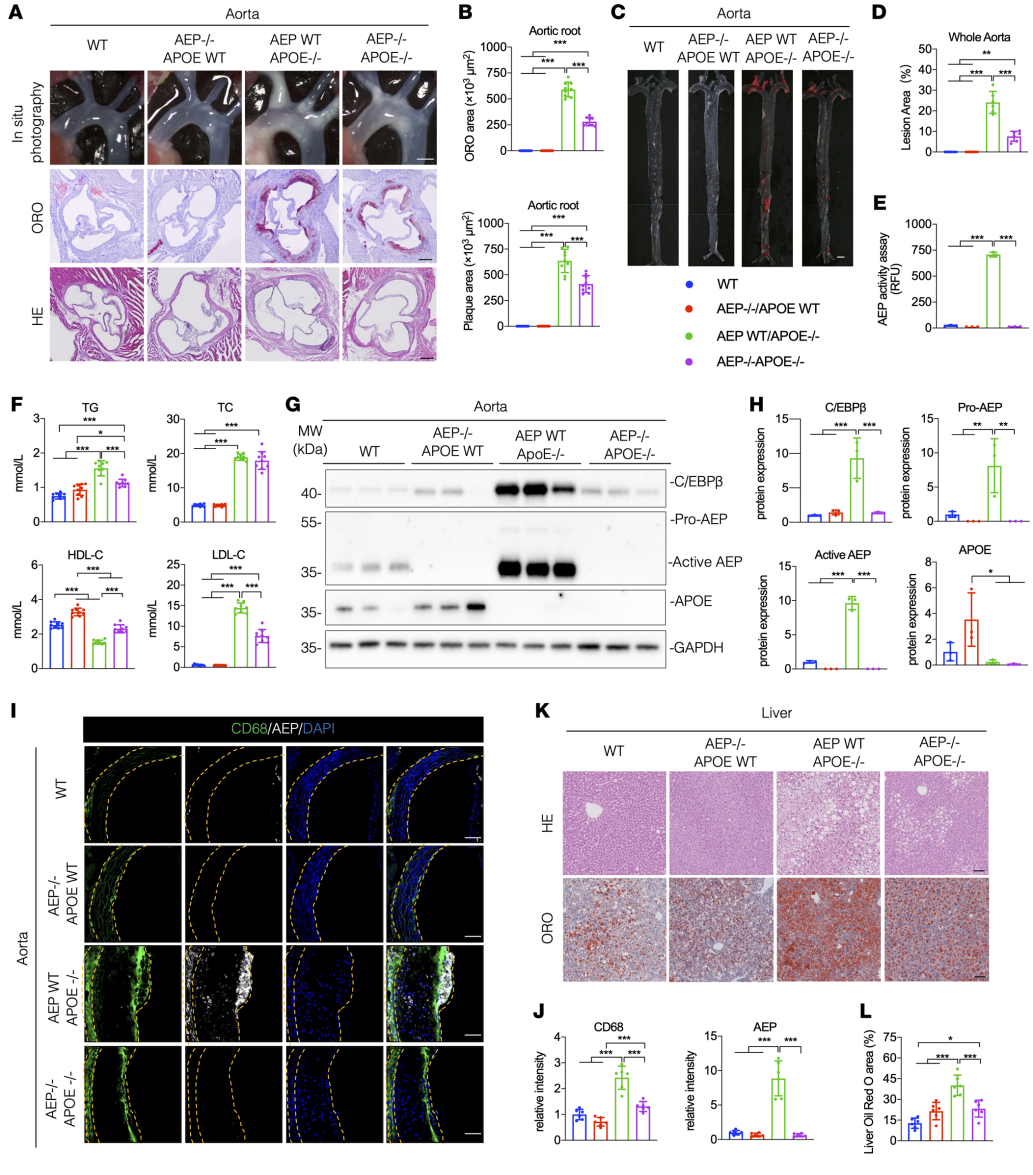
Depletion of AEP from APOE^–/–^ mice attenuates atherosclerosis. (**A**–**L**) WT, AEP^–/–^, APOE^–/–^, and AEP^–/–^APOE^–/–^ mice were fed with a HFD for 12 weeks. (**A**) Representative macroscopic images of aortic arch and aortic root stained with H&E and ORO. Scale bars: 1 mm (top); 25 μm (bottom 2 rows). (**B**) Quantification of aortic plaque and ORO area in aortic root (*n* = 9 per group). (**C**) Representative macrographs of aorta stained with ORO. Scale bar: 1 mm. (**D**) Quantification of aortic plaque area of whole aorta (*n* = 6 per group). (**E**) AEP enzymatic activities of aorta (*n* = 3 per group). (**F**) Serum levels of total cholesterol (TC), triglyceride (TG), LDL-cholesterol (LDL-C) and HDL-C (*n* = 9 per group). (**G** and **H**) Western blot images and quantification of C/EBP-β, AEP and APOE levels in aorta (*n* = 3 per group). (**I** and **J**) Immunofluorescence staining and quantification of CD68 (green) and AEP (red) in aorta. Nuclei were counterstained with DAPI (blue) (*n* = 6 per group). Scale bars: 20 μm. (**K** and **L**) H&E and ORO staining of liver and quantification (*n* = 6 per group). Scale bars: 100 μm. All data are presented as the mean ± SEM. 1-way ANOVA with Tukey’s post hoc test (**B**, **D**–**F**, **H**, **J**, and **K**). *P < 0.05; **P < 0.01; ***P < 0.001.

To assess the pathological roles of AEP in atherosclerosis development in the aorta in APOE^–/–^ mice, we crossed APOE^–/–^ mice with AEP^–/–^ mice and subsequently fed WT mice and single- and double-KO mice with a HFD consecutively for 12 weeks. In vivo AEP enzymatic assay with a fluorescent probe LE28 (32) revealed that AEP was strongly activated in the aorta, kidney, and liver tissues from APOE^–/–^ mice. As expected, these signals were completely eradicated when AEP was deleted (Supplemental Figure 2A). ORO and H&E staining revealed that depletion of AEP from APOE^–/–^ mice prominently reduced ORO areas and plaque areas in the aortic root. There were no atherosclerotic lesions found in either WT or AEP^–/–^ APOE WT mice (Figure 2, A and B). ORO staining with whole aorta showed that AEP deletion greatly eradicated the lesion area from APOE^–/–^ mice, and only APOE^–/–^ mice exhibited demonstrable AEP activation (Figure 2, C–E). Enzymatic assay analysis found that both triglycerides (TGs) and total cholesterols (TCs) were strongly elevated in APOE^–/–^ mice, and knockout of AEP from APOE^–/–^ mice significantly reduced TG but not TC in the serum. LDL-cholesterol (LDL-C) displayed a similar format to TG, whereas HDL-cholesterol (HDL-C) levels were significantly decreased in APOE^–/–^ mice, which were partially restored in APOE^–/–^AEP^–/–^ mice. These biochemical indices remained comparable between WT and AEP^–/–^ mice (Figure 2F). Fast protein liquid chromatography (FPLC) was performed to determine the distribution of cholesterol, triglycerides, and APOA1 across the lipoprotein spectrum (Supplemental Figure 2B). The distribution of cholesterol and triglycerides showed a similar pattern with the results of enzymatic assay analysis (Figure 2F). APOA1 was eluted exclusively with the HDL fraction, and APOA1 levels were lower in both AEP WT APOE^–/–^ and AEP^–/–^ APOE^–/–^ mice compared with WT and AEP^–/–^APOE WT mice (Supplemental Figure 2B). IB analysis with aorta lysates showed that elevated C/EBP-β in APOE^–/–^ mice was strongly repressed in double-KO mice (Figure 2, G and H). IF costaining showed that CD68-positive macrophages were significantly decreased in double-KO mice compared with APOE^–/–^ mice (Figure 2, I and J). The costaining analysis of endothelial cell marker (CD31) and smooth muscle cells marker (α-SMA) with macrophage marker (CD68) revealed that AEP was predominantly expressed in macrophages. Moreover, following plaque formation, the number of endothelial cells and smooth muscle cells declined, whereas the number of CD68-positive cells increased (Supplemental Figure 2, C and D). Liver ORO and H&E staining also revealed similar patterns, suggesting that depletion of AEP greatly abrogates HFD-induced lipid accumulation in liver of APOE^–/–^ mice (Figure 2, K and L). Therefore, AEP is required for HFD-induced atherosclerosis in APOE^–/–^ mice.

### AEP cleaves APOA1 at N208 and impairs cholesterol efflux and HDL formation.

**Figure 3:**
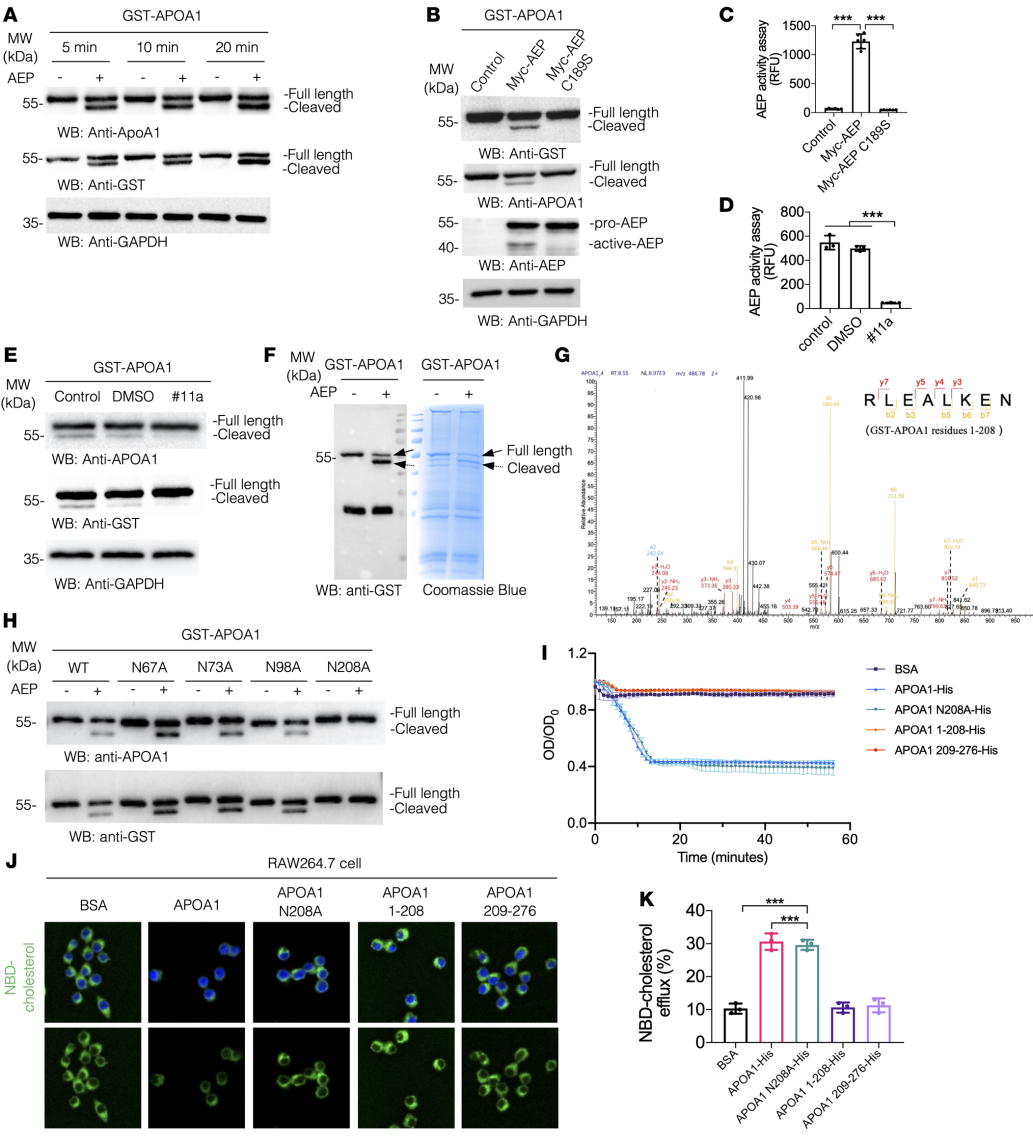
AEP cleaves APOA1 at N208 and severely impairs cholesterol efflux and HDL formation. (**A**) HEK293 cells lysates overexpressing GST-APOA1 were incubated with AEP for 5 minutes, 10 minutes, and 20 minutes. Western blot showing the cleavage of APOA1 by recombinant AEP in a time-dependent manner. (**B**) Cells cotransfected with GST-APOA1 and myc-AEP WT or myc-AEP C189S. Western blot showing that WT AEP but not C189S mutant AEP cleaved GST-APOA1. (**C**) AEP activity was diminished by the C189S mutant of AEP. (**D**) Cleavage of APOA1 was blocked by AEP inhibitor #11a. (**E**) AEP activity was inhibited by #11a. (**F**) Cleavage of purified GST-APOA1 analyzed by immunoblotting (left panel) or Coomassie blue staining (right panel). (**G**) Proteomic analysis of APOA1 recombinant proteins processed by AEP. The detected peptide sequences indicate that N208 is the main cleavage site with the shed bands of molecular weight (MW) 50 kDa. (**H**) Cell lysates overexpressing GST-APOA1 WT, APOA1 mutant (N67A, N73A, N98A, N208A) were incubated with AEP. Western blot showing that the N208A mutant blocked the cleavage. (**I**) DMPC multilamellar vesicles were incubated with BSA alone (control, purple), APOA1 (blue), APOA1 N208Al (green), APOA1 1-208 (orange)or APOA1 209-276 (red). Ability to solubilize DMPC was determined. (**J**) Representative fluorescent images of the NBD-cholesterol burden in RAW264.7 obtained in the indicated group after incubation with APOA1, APOA1 N208A, APOA1 1–208, or APOA1 209–276 for 4 hours. Original maginification, ×20. (**K**) Quantification of NBD-cholesterol efflux. All data are presented as the mean ± SEM from 3 independent experiments. 1-way ANOVA with Tukey’s post hoc test (**C**, **D**, and **K**).*P < 0.05; **P < 0.01; ***P < 0.001.

These observations indicate that APOA1 is impaired in the lesion. To explore whether elevated active AEP could directly cleave APOA1, we conducted a proteolytic assay with GST-APOA1 and recombinant AEP proteins for different time points. IB analysis showed that APOA1 was time-dependently cleaved, revealed by both anti-APOA1 and anti-GST antibodies (Figure 3A). AEP is a cysteine protease with C189 as the key active residue. Cotransfection demonstrated that GST-APOA1 was evidently cleaved by Myc-AEP, and this process was abolished in dominant-negative C189S mutant–transfected cells, underscoring that AEP is accountable for cutting APOA1 into fragments. AEP enzymatic activities in these cells were validated by in vitro assay (Figure 3, B and C). To further confirm that AEP is responsible for APOA1 proteolytic truncation, we found that its specific inhibitor, #11a, abrogated APOA1 fragmentation. #11a is an inhibitor of AEP developed in-house through high-throughput screening and modification (IC_50_ approximately 5–10 nM), which can specifically inhibit AEP activity without affecting other cysteine proteases (18, 33). As expected, #11a strongly blocked AEP enzymatic activity (Figure 3, D and E). To determine the exact cutting sites on APOA1 by AEP, we purified GST-APOA1 recombinant proteins and conducted an AEP cleavage assay, and the cleaved band was validated by IB and Coomassie blue staining (Figure 3F). LC/MS/MS study with the AEP-cleaved APOA1 fragment revealed that N208 was the proteolytic cutting site (Figure 3G). APOA1 possesses numerous N residues in the polypeptide. Only mutation of N208 but not other locations totally blocked APOA1 cleavage by AEP (Figure 3H), which suggests that N208 in APOA1 as a major cutting site by AEP. Accordingly, we generated a rabbit polyclonal antibody that specifically recognized N208 in APOA1. After affinity purification, we showed that anti-APOA1 N208 antibody selectively labeled the fragmented, but not full-length, APOA1 (Supplemental Figure 3A). The antibody specificity was further corroborated by IHC staining in the presence of antigen peptide (aa. 198–208 from APOA1), which completely stripped IHC staining signals on APOE^–/–^ liver tissues (Supplemental Figure 3B). An in vitro DMPC (1,2-dimyristoyl-sn-glycero-3-phosphocholine) multilamellar vesicle assay was used to measure the ability of APOA1 or APOA1 fragments to bind lipids; the results showed that truncated APOA1 1–208 or APOA1 209–276 recombinant proteins failed to solubilize DMPC and was similar to the control, whereas full-length APOA1 or uncleavable mutant N208A effectively cleared DMPC multilamellar vesicles (Figure 3I), indicating that fragmentation of APOA1 at N208 sabotages HDL formation capability by APOA1. The recombinant protein purity was validated by IB with anti-APOA1 antibody (Supplemental Figure 3C). Next, we performed an NBD-cholesterol efflux assay with RAW264.7 cells and found that both APOA1 full-length and N208A mutant strongly mediated fluorescent cholesterol efflux compared with N208-truncated N-terminal or C-terminal fragments (Figure 3, J and K). APOA1 is a major constituent protein of HDL, and coincubation of AEP with HDL also produces a fragment of APOA1 (Supplemental Figure 3D). Hence, AEP cuts APOA1 at the N208 residue and impairs its cholesterol efflux effect, diminishing HDL formation.

### APOA1 is cleaved by AEP in the liver of APOE^–/–^ mice.

**Figure 4:**
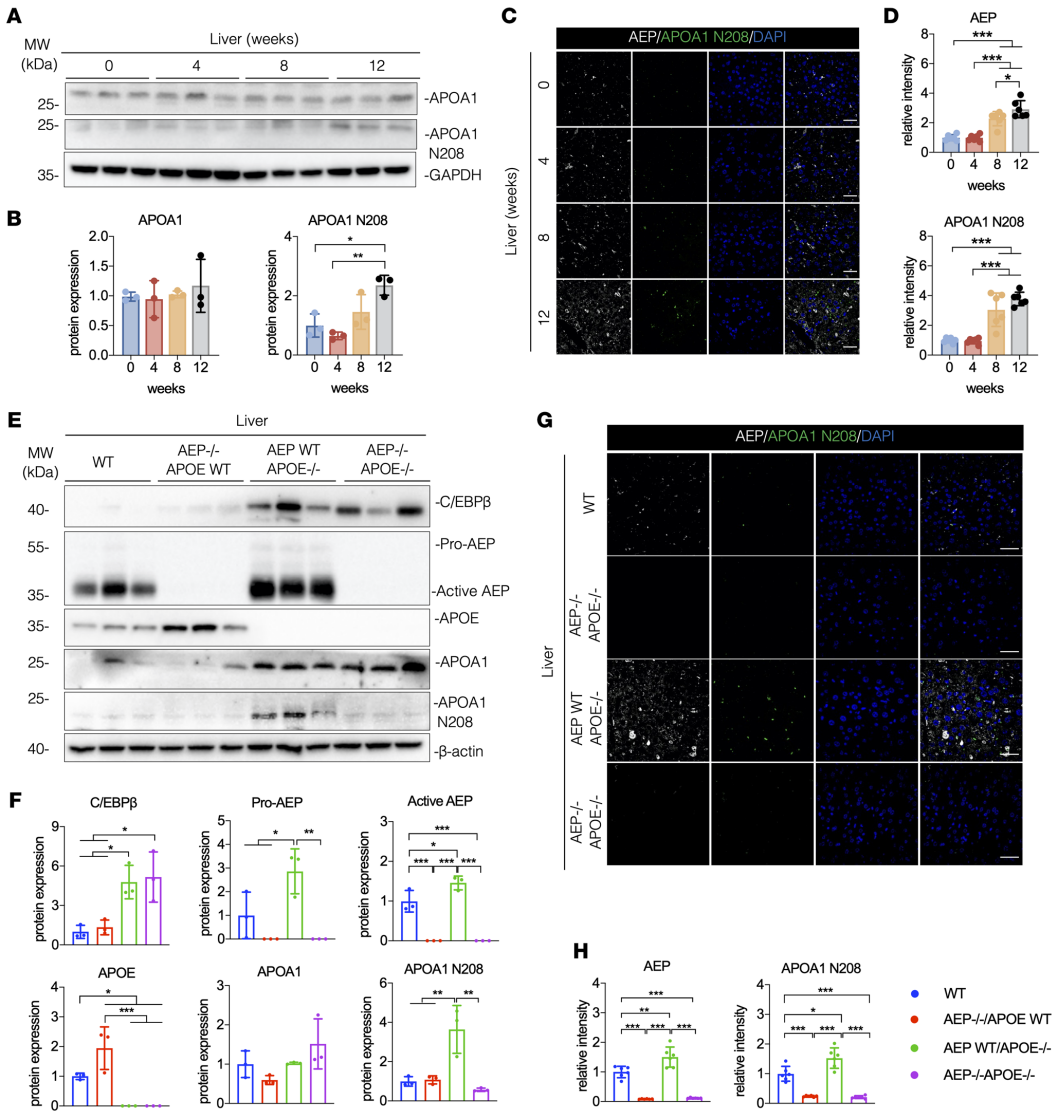
Increased APOA1 cleavage in the liver of APOE^–/–^ mice. (**A**–**D**) APOE^–/–^ mice were fed with a HFD for 0–12 weeks. (**A** and **B**) Western blot images and quantification of APOA1 and APOA1 N208 levels in liver (*n* = 3 per group). (**C** and **D**) Immunofluorescence staining and quantification of AEP (white) and APOA1 N208 (red) in aorta. Nuclei were counterstained with DAPI (blue) (*n* = 6 per group). Scale bars: 20 μm. (**E**–**H**) WT, AEP^–/–^, APOE^–/–^, and AEP^–/–^APOE^–/–^ mice were fed with HFD for 12 weeks. (**E** and **F**) Western blot images and quantification of C/EBP-β, AEP, APOA1 and APOA1 N208 levels in liver (*n* = 3 per group). (**G** and **H**) IF staining and quantification of AEP (white) and APOA1 N208 (red) in liver. Nuclei were counterstained with DAPI (blue) (*n* = 6 per group). Scale bars: 20 μm. All data are presented as the mean ± SEM. 1-way ANOVA with Tukey’s post hoc test (**B**, **D**, **F**, and **H**). *P < 0.05; **P < 0.01; ***P < 0.001.

APOA1 is predominantly synthesized in the liver. To investigate whether endogenous APOA1 in the liver is also cleaved by AEP, we performed IB analysis and found that APOA1 is time-dependently cleaved in the liver of APOE^–/–^ mice fed a HFD for 0–12 weeks, whereas the total APOA1 levels remained relatively stable among the groups (Figure 4, A and B). IF costaining showed that both AEP and truncated APOA1 N208 signals were progressively elevated in the liver (Figure 4, C and D), suggesting that active AEP robustly cleaves APOA1 at N208 site in the liver. Compared with WT mice, total APOA1 levels were strongly escalated in the liver of APOE^–/–^ mice; consequently, APOA1 N208 was prominently fragmented. In agreement with these findings, C/EBP-β/AEP signaling in APOE-null mice was augmented. Depletion of AEP from APOE-null mice abolished N208 cleavage, while total APOA1 and C/EBP-β levels remained unchanged (Figure 4, E and F). IF costaining with liver sections showed that AEP and APOA1 N208 fluorescent intensities tightly coupled to IB signals (Figure 4, G and H). Hence, AEP cleaves APOA1 at N208 in liver of APOE^–/–^ mice.

### APOA1 cleavage by AEP is augmented in the macrophages in the atherosclerotic plaques.

**Figure 5:**
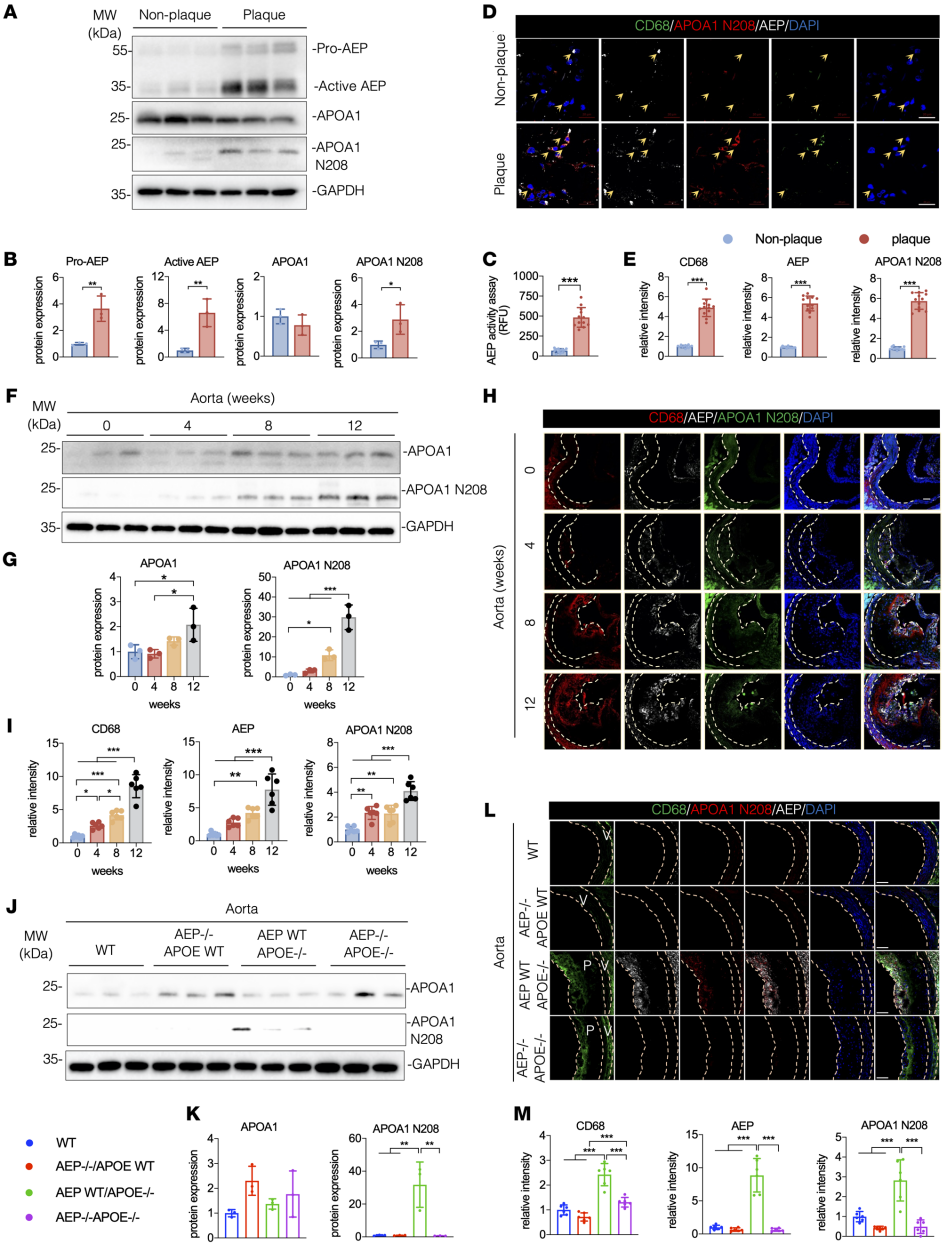
Elevated APOA1 cleavage in the plaques from patients and mice with atherosclerosis. (**A**–**E**) Aortic tissue was taken from patients with atherosclerosis and analyzed. (**A** and **B**) Western blot images and quantification of AEP, APOA1, and APOA1 N208 levels in patients (*n* = 3 per group). (**E**) AEP enzymatic activities of aorta (*n* = 12 per group). (**D** and **E**) IF staining and quantification of CD68 (green), AEP (white), and APOA1 N208 (red) in aorta. The nuclei were counterstained with DAPI (blue) (*n* = 12 per group). Scale bars: 20 μm. (**F**–**I**) APOE^–/–^ mice were fed with a HFD for 0–12 weeks. (**F** and **G**) Western blot images and quantification of APOA1 and APOA1 N208 levels in aorta (*n* = 3 per group). (**H** and **I**) Immunofluorescence staining and quantification of CD68 (green), AEP (white), and APOA1 N208 (red) in aorta. The nuclei were counterstained with DAPI (blue) (*n* = 6 per group). Scale bars: 20 μm. (**J**–**M**) WT, AEP^–/–^, APOE^–/–^, and AEP^–/–^APOE^–/–^ mice were fed with a HFD for 12 weeks. (**J** and **K**) Western blot images and quantification of C/EBP-β, AEP, APOA1, and APOA1 N208 levels in aorta (*n* = 3 per group). (**L** and **M**) Immunofluorescence staining and quantification of CD68 (green), AEP (white), and APOA1 N208 (red) in aorta. Nuclei were counterstained with DAPI (blue) (*n* = 6 per group). Scale bars: 20 μm. All data are presented as the mean ± SEM. 2-tailed, unpaired *t* test (**B**, **C**, and **E**); 1-way ANOVA with Tukey’s post hoc test (**G**, **I**, **K**, and **M**). *P < 0.05; **P < 0.01; ***P < 0.001.

Most of APOA1 within atherosclerotic human arterial tissue, in contrast to within the circulation, is lipid-poor and does not reside on HDL-like particles (30). It is possible that APOA1 in the lesion areas is truncated and dysfunctional. To test this notion, we performed IB with healthy control and patient tissues with atherosclerotic plaques and found that APOA1 N208 levels were greatly enhanced in the plaque compared with nonplaque arterial tissues, which was accompanied with conspicuously elevated active AEP, whereas full-length APOA1 remained comparable between the groups (Figure 5, A and B). Quantification revealed that AEP enzymatic activity was higher in the plaques than nonplaques (Figure 5C). IF costaining demonstrated that both AEP and APOA1 N208 activities were significantly increased in CD68-positive macrophages in plaques versus nonplaques (Figure 5, D and E). IB analysis with tissue from the aorta from APOE^–/–^ mice revealed that both full-length APOA1 and its N208 fragment were gradually increased upon HFD treatment (Figure 5, F and G). IF costaining showed that CD68, active AEP, and APOA1 N208 fluorescent activities were progressively escalated in the aorta in APOE^–/–^ mice (Figure 5, H and I). Consistently, deletion of AEP from APOE^–/–^ mice abrogated APOA1 N208 cleavage in the aorta (Figure 5, J and K). Again, AEP depletion significantly reduced CD68 and APOA1 N208 fluorescent signals in the aorta from APOE^–/–^/AEP^–/–^ mice versus APOE^–/–^ mice (Figure 5, L and M). Therefore, macrophages are greatly increased in both human atherosclerotic plaques and in the aorta from APOE^–/–^ mice, associated with augmented APOA1 N208 and active AEP.

### Blockade of APOA1 cleavage by AEP diminishes atherosclerosis in APOE^–/–^ mice.

**Figure 6:**
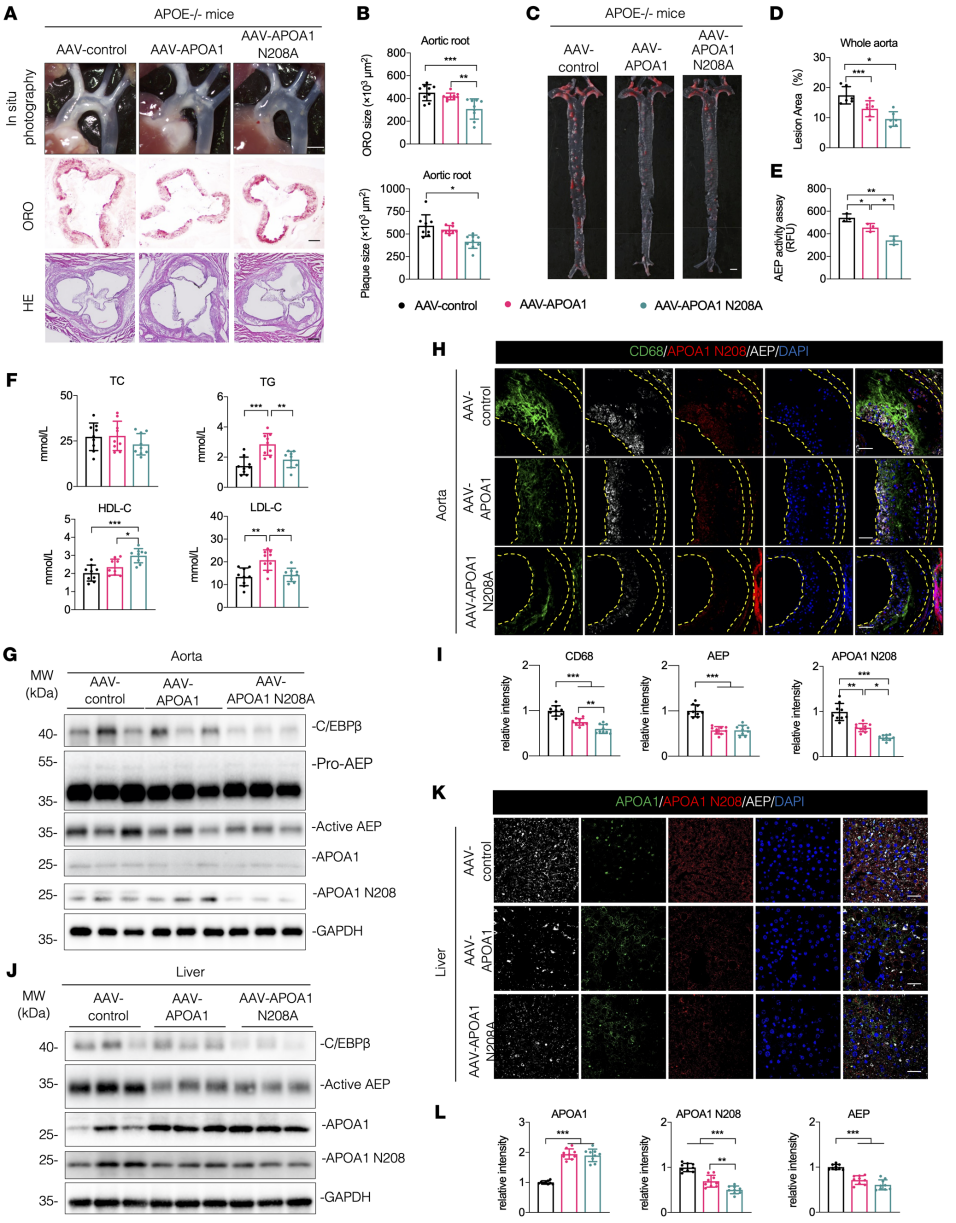
Blockade of APOA1 cleavage by AEP attenuates atherosclerosis in APOE^–/–^ mice. (**A**–**L**) APOE^–/–^ mice were injected with AAV-control, AAV-APOA1, or AAV-ApoA 1 N208A virus at 6-week-old and fed with HFD for 12 weeks beginning at 8-weeks old. (**A**) Representative macroscopic images of aortic arch and aortic root stained with H&E and ORO. Scale bars: 1 mm (top); 25 μm (bottom 2 rows). (**B**) Quantification of aortic plaque and ORO area in aortic root (*n* = 9 per group). (**C**) Representative macrographs of aorta stained with ORO. Scale bar: 1 mm. (**D**) Quantification of aortic plaque area of whole aorta (*n* = 6 per group). (**E**) AEP enzymatic activities of aorta (*n* = 3 per group). (**F**) Serum levels of total cholesterol (TC), triglyceride (TG), LDL-cholesterol (LDL-C) and HDL-C (*n* = 9 per group). (**G**) Western blot analysis of C/EBP-β, AEP, APOA1, and APOA1 N208 levels in aorta (*n* = 3 per group). (**H** and **I**) IF staining and quantification of CD68 (green), AEP (white), and APOA1 N208 (red) in aorta. The nuclei were counterstained with DAPI (blue) (*n* = 9 per group). Scale bars, 20 μm. (**J**) Western blot analysis of C/EBP-β, AEP, APOA1, and APOA1 N208 levels in liver (*n* = 3 per group). (**K** and **L**) IF staining and quantification of AEP (white) and APOA1 N208 (red) in liver. Nuclei were counterstained with DAPI (blue) (*n* = 9 per group). Scale bars, 20 μm. All data are presented as the mean ± SEM. 1-way ANOVA with Tukey’s post hoc test (**B**, **D**–**F**, **I**, and **L**). *P < 0.05; **P < 0.01; ***P < 0.001.

To examine the pathological roles of APOA1 cleavage by AEP in the aorta, we infected APOE^–/–^ mice with AAV-APOA1 and uncleavable AAV-APOA1 N208A via tail vein injection, followed by HFD treatment for 12 weeks. These animals exhibited comparable body weight growth curves, albeit N208A mice displayed higher food intake than APOA1 mice. In contrast to AAV-control and AAV-APOA1–infected mice, lipid deposition in the aortic root in N208A mice was clearly decreased. Both ORO size and plaque size were significantly diminished in N208A groups compared with control and APOA1 full-length groups (Figure 6, A and B). ORO staining of the whole aorta (en face measurement) demonstrated a significantly attenuated lesion area in N208A mice compared with the control group. Full-length APOA1 showed the reduction trend but it was not statistically significant (Figure 6, C and D). Interestingly, AEP enzymatic activities were decreased in both APOA1 and N208A groups, with a greater decrease in the N208A group (Figure 6E). Though TC levels remained the same among the groups, both TG and LDL-C levels were highly augmented in full-length APOA1–expressing mice compared with the 2 other groups. By contrast, HDL-C levels were significantly increased in N208A mice (Figure 6F and Supplemental Figure 4A). There was no significant difference in the distribution of APOA1 in mouse serum (Supplemental Figure 4A). IB analysis with aorta and liver tissues from these mice showed that C/EBP-β/AEP signaling was significantly suppressed in N208A mice compared with control mice (Figure 6, G and J, and Supplemental Figure 4, B and C). Though total APOA1 levels in the aorta were similar among the groups, N208 fragmentation was significantly diminished in N208A mice versus control and full-length APOA1 mice (Figure 6G and Supplemental Figure 4B). Notably, both APOA1 and N208A levels in the liver were elevated compared with control mice; nevertheless, endogenous APOA1 N208 cleavage was reduced in the uncleavable N208A mutant group, without statistical significance (Figure 6J and Supplemental Figure 4C), indicating that the N208A mutant strongly blunts APOA1 cleavage by AEP in the aorta compared with the liver. IF costaining with aorta sections demonstrated that both macrophages and active AEP were significantly attenuated in APOA1 and N208A groups compared with control; APOA1 and N208 fluorescent intensities were increasingly diminished from the control to APOA1 to the N208A group (Figure 6, H and I). On liver sections, we found that APOA1 fluorescent intensities were highly increased in both APOA1 and N208A groups compared with the control group, inversely correlated with attenuated N208 cleavage and active AEP signals (Figure 6, K and L). Remarkably, liver staining with H&E and ORO showed the lipid deposition was greatly reduced in N208A group. Quantification revealed ORO area was significantly decreased in N208A group compared with the other 2 groups (Supplemental Figure 4D). Ex vivo AEP enzymatic assay with LE28 probe indicated that AEP activities were increasingly reduced from control to APOA1 to N208A groups, fitting with immunoblotting observations and APOA1 N208 signals (Supplemental Figure 4E).

### AEP inhibitor #11a decreases atherosclerosis in APOE^–/–^ and LDLR^–/–^ mice.

**Figure 7:**
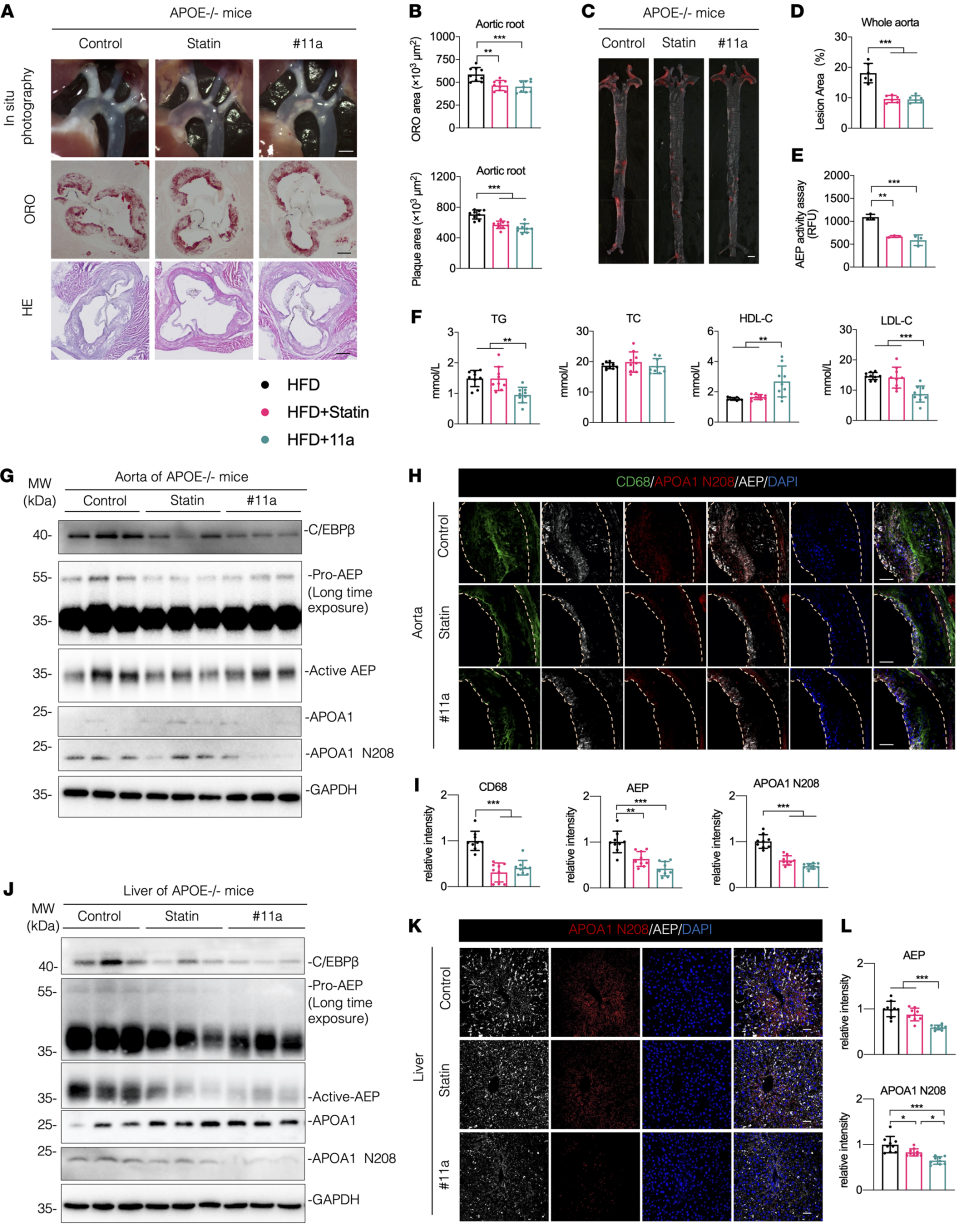
Inhibition of AEP with specific inhibitor #11a attenuates atherosclerosis in APOE^–/–^ mouse model. (**A**–**L**) APOE^–/–^ mice were fed with a HFD (control), HFD + statin (Statin, 10.0 mg/kg) or HFD + #11a (#11a, 7.5 mg/kg) for 12 weeks beginning at 8-weeks old. (**A**) Representative macroscopic images and quantification of aortic arch and aortic root stained with H&E and ORO. scale bars: 1 mm(top;) 25 μm(bottom 2 rows). (**B**) Quantification of aortic plaque and ORO area in aortic root (*n* = 9 per group). (**C** and **D**) Representative macrographs and quantification of aorta stained with ORO (*n* = 6 per group). Scale bar: 1 mm. (**E**) AEP enzymatic activities of aorta (*n* = 3 per group). (**F**) Serum levels of total cholesterol (TC), triglyceride (TG), LDL-cholesterol (LDL-C) and HDL-C (*n* = 9 per group). (**G**) Western blot analysis of C/EBP-β, AEP, APOA1, and APOA1 N208 levels in aorta (*n* = 3 per group). (**H** and **I**) IF staining and quantification of CD68 (green), AEP (white), and APOA1 N208 (red) in aorta. Nuclei were counterstained with DAPI (blue) (*n* = 9 per group). Scale bars: 20 μm. (**J**) Western blot analysis of C/EBP-β, AEP, APOA1, and APOA1 N208 levels in liver (*n* = 3 per group). (**K** and **L**) IF staining and quantification of AEP (white) and APOA1 N208 (red) in liver. Nuclei were counterstained with (blue) (*n* = 9 per group). Scale bars: 20 μm. All data are presented as the mean ± SEM. 1-way ANOVA with Tukey’s post hoc test (**B**, **D**–**F**, **I**, and **L**). *P < 0.05; **P < 0.01; ***P < 0.001.

Our laboratory developed the AEP-specific inhibitor #11a through high-throughput screening and chemical modification. #11a specifically inhibits AEP enzyme activity and does not affect other cysteine enzymes (18, 34). Chronic treatment of Parkinson’s disease mice with #11a markedly inhibits AEP activity and increasing TH-positive dopaminergic neurons. To ascertain the pathological roles of AEP in atherosclerosis, we fed 2-month-old APOE^–/–^ mice with a HFD mixed with #11a (7.5 mg/kg) or statin (10.0 mg/kg) for 12 weeks. Statins are well-known lipid-lowering drugs by inhibiting 3-hydroxy-3-methylglutaryl coenzyme-A (HMG-CoA) reductase (35), which is widely used in clinical practice as a lipid-lowering medication for managing ATH. In this study, atorvastatin was used as comparison medicine to explore whether #11a could ameliorate ATH as effectively as statin does. There was no significant difference in the body weights of several groups of mice (Supplemental Figure 5A). Compared with the control group, both statin and #11a significantly reduced ORO area and plaque area in the aortic root (Figure 7, A and B). ORO staining also revealed that lesion areas in the whole aorta were highly decreased by statin or #11a (Figure 7, C and D). Consequently, AEP activities were substantially blocked by statin or #11a (Figure 7E). Remarkably, TG and LDL-C but not TC were conspicuously reduced by #11a. By contrast, HDL-C levels were strongly augmented by #11a compared with the control or statin groups (Figure 7F and Supplemental Figure 5C). APOA1 was slightly elevated in the serum of statin-treated and #11a-treated groups of mice. To evaluate potential off-target effects of #11a, we administered it to AEP^–/–^APOE^–/–^ mice. The results indicated that #11a did not further reduce plaque levels in these mice (Supplemental Figure 6, A–E). IB analysis with aorta tissue showed APOA1 N208 levels were prominently inhibited by #11a, so was the C/EBP-β/AEP pathway (Figure 7G and Supplemental Figure 5D). IF costaining disclosed that CD68, AEP, and N208 fluorescent intensities were notably blocked by statin or #11a (Figure 7, H and I). IB analysis with liver lysates indicated that APOA1 N208 cleavage was highly blocked by statin or #11a, conversely correlated with escalation of full-length APOA1 levels. As expected, C/EBP-β/AEP signaling was consequently suppressed. Consistently, AEP activities were significantly antagonized by statin or #11a (Figure 7J and Supplemental Figure 5, E and F). In alignment with IB findings, IF costaining of liver sections demonstrated that AEP and N208 fluorescent signals were substantially repressed by #11a (Figure 7, K and L). H&E and ORO staining of liver sections indicated that lipid accumulation and ORO areas were greatly reduced by the drug treatment (Supplemental Figure 5G). Ex vivo AEP enzymatic assay with LE28 demonstrated that #11a strongly blunted AEP activities in aorta. Both statin and #11a evidently reduced AEP activities in the liver of APOE^–/–^ mice (Supplemental Figure 5H).

LDLR^–/–^ mice are another commonly used model mouse for atherosclerosis, and we also examined the effect of #11a on the development of atherosclerosis in LDLR^–/–^ mice. We fed 2-month-old LDLR^–/–^ mice with a HFD mixed with #11a (7.5 mg/kg) or statin (10.0 mg/kg) for 12 weeks. Treatment with #11a increased the concentration of #11a in serum (Supplemental Figure 8A). We made similar observations in the aortic root for both ORO, plaque, and lesion areas as we made in the whole aorta. Both statin and #11a greatly reduced atherosclerosis and AEP activities (Supplemental Figure 7, A–E). Consequently, TG, TC, and LDL-C levels were substantially blocked by these 2 compounds. By contrast, HDL-C levels were inversely escalated (Supplemental Figure 7F). IB and IF costaining demonstrated that #11a robustly suppressed C/EBP-β/AEP signaling and N208 cleavage in the aorta from LDLR^–/–^ mice. The macrophages were evidently decreased by both compounds in the aorta as well (Supplemental Figure 7, G–I). IB and IF costaining with liver from LDLR^–/–^ mice revealed the similar observations to those in aorta (Supplemental Figure 7, J–L, and Supplemental Figure 8, B and C). Again, #11a significantly repressed AEP enzymatic activity in the liver (Supplemental Figure 8D). The lipid deposition and ORO area in the liver were substantially reduced by both statin and #11a (Supplemental Figure 8E). Together, these results strongly support that AEP inhibitor #11a displays a comparable antiatherosclerosis therapeutic effect to statin in the LDLR^–/–^ model.

## Discussion

In the current work, we demonstrate that AEP is greatly augmented in aortic plaques from patients with atherosclerosis and elevation of AEP is also observed in liver and plaques of mouse models with atherosclerosis. We show that AEP cleaves APOA1 at N208 residue in the liver and aorta, and APOA1 1-208 fragment loses its ability to transport cholesterol, because the last helix of APOA1 is critical for its ability to efflux cholesterol by ABCA1 (36). Blockade of APOA1 cleavage by AEP inhibitor #11a or by overexpressing the uncleavable APOA1 N208A mutant increases HDL-C levels and delays atherosclerosis progression. Statin enhances the ability of HDL to promote cholesterol efflux from hepatoma cells, but its effect on cholesterol efflux from macrophages is inconsistent (37, 38). It has been proposed that statins interfere with HDL-mediated macrophage cholesterol efflux through ABCA1-induced pathways (39). Remarkably, inhibition of AEP by #11a not only substantially decreases TG and LDL-cholesterol levels in the blood but also highly increases HDL-cholesterol concentrations in APOE^–/–^ mice compared with statin, suggesting that inhibition of AEP may be a therapeutic strategy for treating atherosclerosis. We made similar observations with statin and #11a in another well-characterized LDLR^–/–^ atherosclerosis mouse model. Nonetheless, these 2 compounds exhibit comparable antiatherosclerosis efficacy with TG, TC, and LDL-C levels significantly lower than vehicle control and HDL-C concentrations higher than control (Supplemental Figures 7 and 8).

HDL exerts its antiatherosclerotic effects via various pathways, such as enhancing endothelial function, facilitating cholesterol removal from macrophages, and providing antioxidant, antiinflammatory, and antiapoptotic properties. Conversely, dysfunctional HDL particles amplify proinflammatory signals and impair cholesterol efflux from macrophages via the ABCA1 pathway. Research has shown that myeloperoxidase-induced oxidation of specific residues on APOA1 leads to the formation of dysfunctional HDL particles, which are linked to a higher risk of cardiovascular events in prospective studies (40). In this work, we show depletion of AEP from APOE^–/–^ mice increases HDL-C levels (Figure 2F), which is consistent with AEP inhibitor #11a’s therapeutic efficacy in APOE^–/–^ and LDLR^–/–^ mice (Figure 7F and Supplemental Figure 7F). Thus, inactivation of AEP restores HDL functions by escalating its cholesterol transport. Our previous experimental results demonstrated that AEP knockout reduced LDL levels, with a modest, though not significant, increase in HDL levels (19). This variation may be attributed to differences in food composition. In our previous study, APOE^–/–^ mice began a HFD (D12079B, 45% fat, 0.5% cholesterol) at 3 months of age (19), whereas in this study, APOE^–/–^ mice were fed with HFD (Clinton-Cybulsky, 40% fat, 1.25% cholesterol) at 8 weeks. Variations in mouse age and the composition of the feed may explain the differences in lipid profiles observed between the 2 studies.

APOA1 is a predominant apolipoprotein constituent of HDLs. In atherosclerotic lesions, the concentration of APOA1 is over 100 times higher than that in normal arterial walls. Moreover, within both normal and atherosclerotic arterial tissues, the majority of APOA1 is oxidatively cross-linked (30). APOA1 relates to lower observational risk of coronary artery disease. However, epidemiological data fail to support a cardioprotective role for APOA1 (41). Alterations in APOA1 through posttranslational modifications and degradation pathways could potentially undermine its therapeutic potential, thereby accounting for the unsuccessful outcomes in certain clinical trials. The posttranslational modification and degradation of APOA1 may contribute to the lack of efficacy in at least some of these failed clinical trials. The posttranslational modifications of APOA1 include glycation, acylation, cleavage, and oxidation. The role of cleavage is discussed below, while reports on the oxidation of APOA1 in cardiovascular disease are very limited (42).

Patients with atherosclerosis exhibit a highly proinflammatory and oxidative state, which activates mast cells and triggers the release of granule-associated proteases. These proteases cleave the C-terminal domain of APOA1, specifically at Ser228, thereby inhibiting its antiinflammatory properties (43). This truncation of APOA1 substantially impairs its ability to solubilize lipids and promote cholesterol efflux via the ABCA1 pathway. Additionally, human macrophage cathepsin B–mediated cleavage of APOA1 at Ser228 severely compromises its antiatherogenic capacity (44). Here, we show that AEP, a cysteine protease, is highly activated in both the liver and aorta of APOE^–/–^ mice and in human atherosclerotic plaques. It strongly cleaves APOA1 at N208 in the liver, where APOA1 is predominantly synthesized (Figure 4). Moreover, we observed elevated active AEP and APOA1 N208 fragment in augmented macrophages in the patient-derived plaques (Figure 5). Again, depletion of AEP from APOE^–/–^ mice blunts APOA1 N208 fragmentation in the aorta from APOE^–/–^AEP^–/–^ mice (Figure 5, J–M). Employing the AEP uncleavable N208A mutant, we showed that the plaques size and lesion area are significantly diminished in the aorta from APOE^–/–^ mice (Figure 6, A–D), underscoring that blockade of APOA1 N208 cleavage is partially accountable for the antiatherosclerotic effect of AEP. In alignment with these findings, HDL-C levels are increased, associated with macrophage reduction in AEP-resistant AAV-APOA1 N208A-infected APOE^–/–^ mice (Figure 6, F–I).

In patients with carotid atherosclerosis, plasma levels of AEP are substantially elevated compared with those in healthy individuals. Moreover, individuals presenting with recent symptoms exhibit higher AEP expression within plaques than asymptomatic patients, suggesting an upregulation during the acute phases of the disease (7). Additionally, AEP levels are higher in unstable plaques compared with stable regions of carotid plaques (5, 45). These findings indicate that AEP plasma levels may serve as a potential biomarker for identifying the presence and characterizing the nature of carotid plaques in atherosclerotic disease (46). A study utilizing whole transcriptome analysis compared stable and unstable segments of human atherosclerotic plaques, revealing a marked increase in AEP expression at both mRNA and protein levels (2). Noticeably, AEP promotes atherosclerotic vascular remodeling (47). These observations are consistent with our findings that AEP is gradually elevated in APOE^–/–^ mice upon HFD treatment. Its enzymatic activities are highly escalated in the aorta, liver, and kidney of APOE^–/–^ mice (Figures 1 and 2, and Supplemental Figure 2A). Macrophage-specific deletion of AEP alleviates β-aminopropionitrile monofumarate–induced (BAPN-induced) extracellular matrix degradation and ameliorates VSMC phenotypic switch in BAPN-treated mice.

AEP, derived from macrophages, interacts with integrin αv-β3 in vascular smooth muscle cells (VSMCs), inhibiting its function. This inhibition reduces Rho GTPase activation and downregulates markers of VSMC differentiation, ultimately worsening the progression of thoracic aortic dissection (TAD). As a result, AEP acts as a natural regulator of integrin αvβ3, promoting vascular degeneration, dissection, and rupture (48). Additionally, AEP enhances the migration of both human monocytes and HUVECs. It also reduces the mRNA expression of vascular cell adhesion molecule-1 (VCAM1), induced by lipopolysaccharide (LPS), while boosting the expression of IL-6 and E-selectin (SELE) in HUVECs. Furthermore, AEP promotes the inflammatory M1 phenotype in macrophages and facilitates the formation of foam cells induced by oxidized low-density lipoprotein. Interestingly, AEP does not affect the proliferation or apoptosis of human aortic smooth muscle cells (HASMCs), but it does increase their migratory activity (47). As expected, elevated AEP in the aorta stimulates monocyte infiltration and escalates inflammation in the lesion, facilitating foam cell formation and atherosclerotic pathologies (Figures 1 and 2). Consequently, inhibition of AEP by its specific inhibitor #11a greatly antagonizes atherosclerosis in both APOE^–/–^ and LDLR^–/–^ mouse models (Figure 7 and Supplemental Figure 8).

AEP is an enzyme with numerous substrates. In various diseases, AEP can target different proteins closely associated with the disease. For instance, our research has shown that AEP cleaves APP and tau, which accelerates Alzheimer’s Disease pathology (16, 17). Similarly, in the context of ATH, there is evidence that AEP cleaves proteins like fibronectin, contributing to plaque instability (6, 49). APOA1 is not the sole substrate of AEP in ATH. APOA1 is an essential structural element of HDL and is critically involved in facilitating cholesterol export from macrophages. In studies using APOE^–/–^AEP^–/–^ mice, we observed increased HDL levels and reduced plaque formation, indicating that AEP-mediated cleavage of APOA1 is a key mechanism through which AEP accelerates ATH by influencing cholesterol metabolism.

Macrophages are pivotal in the progression of atherosclerosis (50), with the formation of foam cells due to disrupted cholesterol metabolism being a key initial step (51, 52). Although extensive research has been conducted, the precise molecular pathways through which arterial macrophages absorb cholesterol-laden lipoproteins, such as LDL, and contribute to foam cell formation and atherosclerotic plaques, are not completely understood. Notably, the transcription factor C/EBP-β in hematopoietic cells is essential for maintaining cholesterol homeostasis in macrophages and in the liver, playing a substantial role in diet-induced inflammation, hyperlipidemia, and the advancement of atherosclerosis. Removing C/EBP-β from hematopoietic cells notably decreases the formation of atherosclerotic plaques in the aortic sinuses of APOE-deficient mice on a high-fat, high-cholesterol diet. This reduction in plaque formation is linked to a marked drop in circulating cytokine levels and a moderate decrease in the expression of proinflammatory and macrophage marker genes in visceral adipose tissue. Furthermore, the absence of C/EBP-β in hematopoietic cells also lowers total serum and LDL-C levels without impacting HDL-C levels (13). In recent studies using the APOE-deficient mouse model fed a HFD, the deletion of C/EBP-β or AEP showed that disrupting the C/EBP-β/AEP pathway substantially reduces ox-LDL, inflammation, macrophage activity, and lesion areas in the proximal aorta and coronary artery (13). These findings underscore the role of the C/EBP-β/AEP signaling pathway in the pathogenesis of atherosclerosis, aligning with earlier studies (7, 13, 45–47).

Both AD and atherosclerosis are interconnected conditions with vascular involvement as a common risk factor, albeit affecting different vascular sites. Recent research has also highlighted that the C/EBP-β/AEP signaling pathway links atherosclerosis risk factors to the development of AD pathologies (19). Interestingly, the antidiabetic drug metformin downregulates C/EBP-β expression in hepatocytes (10). AEP is highly activated in the liver of APOE^–/–^ mice (Figure 1). Inactivation of C/EBP-β predominantly represses AEP levels (12). Undoubtedly, blocking its upstream transcription factor C/EBP-β will suppress its activation. Nonetheless, investigating whether the therapeutic effects of these and other antiatherosclerotic or antidiabetic medications are linked to their influence on C/EBP-β represents a promising direction for further research. In both APOE^–/–^ and LDLR^–/–^ mice, we show that AEP inhibitor #11a potently decreases C/EBP-β levels in the liver and aorta (Figure 7 and Supplemental Figures 5, 7, and 8). Because C/EBP-β is a major transcription factor for various inflammatory cytokines (53), conceivably, blockage of its activity may substantially repress inflammation in these atherosclerotic mouse models. Our findings indicate that inhibiting AEP or preventing the cleavage of APOA1 at N208 by AEP reduces C/EBP-β levels (Figures 2, 6, and 7, and Supplemental Figure 7). This aligns with prior research showing that C/EBP-β in hematopoietic cells promotes atherosclerosis by driving the expression of inflammatory genes and disrupting lipid metabolism (54). Taken together, our work strongly supports that C/EBP-β/AEP signaling plays a critical role in atherosclerosis pathogenesis, and blockade of AEP with its specific inhibitor may provide a powerful therapeutic agent for treating this cardiovascular disorder.

## Methods

### Sex as a biological variable.

For animal models, only male mice were used to avoid the potential variability introduced by the estrous cycle in female mice. For clinical samples, both sexes were involved. The sex was not considered as a biological variable.

### Animals.

APOE^–/–^ mice and LDLR^–/–^ mice on a C57BL/6J background were obtained from Cyagen Biosciences. AEP knockout mice (obtained from the laboratory of Zhentao Zhang at Wuhan University) on a mixed 129/Ola and C57BL/6 background were generated as reported (55). We crossed APOE^–/–^ mice with AEP^–/–^ mice to produce APOE^+/–^ AEP^+/–^ offspring and then intercrossed these mice to produce APOE^–/–^ AEP^–/–^ mice, APOE^–/–^ AEP WT mice, AEP^–/–^ APOE WT mice, or WT littermates. The following animal groups were analyzed: WT, AEP^–/–^, APOE^–/–^, and APOE^–/–^ AEP^–/–^ mice. Eight-week-old male mice were used for experiments and fed with Western diet (0.2% (w/w) cholesterol, *n* = 6) (TD.88137, Nantong Trophic Animal Feed High-Tech Co. Ltd.) unless otherwise mentioned. Mice were housed on a 12-hour light/dark cycle and had free access to water and food. For the treatment of #11a, APOE^–/–^ mice were separated into 3 groups and were fed a Western diet (0.2% (w/w) cholesterol, *n* = 6), a diet with statin (Western diet + 100 mg/kg (w/w) statin, *n* = 6), or a diet with #11a (Western diet + 75 mg/kg (w/w) #11a, *n* = 6) for 12 weeks. Foods were changed and recorded weekly, and body weight was recorded weekly.

### Human tissue samples.

Atherosclerotic plaque samples were collected from patients undergoing coronary endarterectomy and immediately snap frozen in liquid nitrogen for Western blot analysis or fixed in 4% PFA for IF staining.

### Atherosclerosis evaluation.

Whole aortas (from the root to the femoral artery bifurcation) were carefully pooled from mice in each group after dissecting perivascular fat from vascular tissue under the dissecting microscope. Three aortas in each group were prepared for Western blot analysis, and the other 3 aortas were placed in 10% formalin for ORO staining to evaluate plaques. The heart was isolated and embedded in OCT compound (Sakura). Serial frozen sections (10 μm) of the aortic root were obtained throughout the region. Then, 10 slides containing aortic sinus tissue samples were prepared from each heart, among which 1–2 slides per heart were processed for ORO staining. For en face analysis, the aorta was opened longitudinally and stained with ORO for 1 hour at room temperature. Plaques were quantified by morphometry of obtained images using Image J.

Serial sections (10 μm thick) of the aortic root (3–5 sections per mouse) and liver were stained with H&E, ORO, and Masson’s trichrome, and then microscopic images were collected. Images were acquired with an Olympus SZ61 microscope digital camera (Olympus) or Axio Scan 7 Scanner (Carl Zeiss Meditec).

### Measurement of blood lipids profile.

Blood samples were collected when mice were euthanized. Serum was separated by centrifugation. Serum triglyceride (Beyotime, S0219M), total cholesterol (Beyotime, S0211M), LDL-C (Abcam, ab65390) and HDL-C (Abcam, ab65390) were measured according to the manufacturer’s instructions. Fast protein liquid chromatography (FPLC) was performed to determine the distribution of cholesterol, triglycerides, and APOA1 across the lipoprotein spectrum. Briefly, 100 μL serum was injected into an ÄKTA FPLC system (GE Healthcare) using a superose 6 10/300 GL column (Cytiva, 29021596) at a flow rate of 0.3 mL/min, with running buffer (0.15 mol/L NaCl, 1 mM EDTA, pH 7.0) and collection of 30 0.5-mL fractions. In each fraction, cholesterol (Abcam, ab65390), triglycerides (Beyotime, S0211M), and APOA1 (Abcam, ab238260) were measured with commercial kits.

### AEP activity assay.

Tissue homogenates or cell lysates (10 μg) were incubated in 200 μL assay buffer (20 mM citric acid, 60 mM Na_2_HPO_4_, 1 mM EDTA, 0.1% CHAPS, and 1 mM DTT, pH 6.0) containing 20 μM δ-secretase substrate Z-Ala-Ala-Asn-AMC (Bachem). AMC released by substrate cleavage was quantified by measuring at 460 nm in a fluorescence plate reader at 37 °C for 2 hours in kinetic mode.

### Western blot analysis.

Cells and aorta sample tissue were washed with ice-cold PBS and lysed in lysis buffer (50 mM Tris, pH 7.4, 40 mM NaCl, 1 mM EDTA, 0.5% Triton X-100, 1.5 mM Na3VO4, 50 mM NaF, 10 mM sodium pyrophosphate, 10 mM sodium β-glycerophosphate, supplemented with protease inhibitors cocktail) at 4°C for 0.5 hours, and centrifuged for 15 minutes at 23,887 ×*g*. The supernatant was boiled in SDS loading buffer. After SDS-PAGE, the samples were transferred to a nitrocellulose membrane. The membrane was blocked with TBS containing 5% nonfat milk and 0.1% Tween 20 (TBST) at room temperature for 1 hour, followed by the incubation with primary antibody at 4°C overnight, and with the secondary antibody at room temperature for 2 hours. After washing with TBST, the membrane was developed using the enhanced chemiluminescent (ECL) detection system. Antibodies used for Western blotting were listed in Supplemental Table 1.

### Mass spectrometry analysis.

Protein samples were in-gel digested with trypsin. Peptide samples were resuspended in loading buffer (0.1% formic acid, 0.03% trifluoroacetic acid, and 1% acetonitrile) and loaded onto a 20-cm nano-high–performance liquid chromatography column (internal diameter 100 mm) packed with Reprosil-Pur 120 C18-AQ 1.9 mm beads (Dr. Maisch) and eluted over a 2 hour 4–80% buffer B reverse-phase gradient (buffer A: 0.1% formic acid and 1% acetonitrile in water; buffer B: 0.1% formic acid in acetonitrile) generated by a NanoAcquity UPLC system (Waters Corporation). Peptides were ionized with 2.0 kV electrospray ionization voltage from a nano-ESI source (Thermo Fisher Scientific) on a hybrid LTQ XL Orbitrap mass spectrometer (Thermo Fisher Scientific). Data-dependent acquisition of MS spectra at 120,000 resolution (full width at half maximum) and tandem mass spectrometry (MS/MS) spectra were obtained in the Orbitrap after electron-transfer dissociation with supplemental activation with high energy (EThcD) for peptide masses. To identify AEP cleavage sites in human APOA1, Proteome Discoverer 2.0 (PD) was used to search and match MS/MS spectra to a complete human proteome database (NCBI reference sequence revision 62, with 68,746 entries) with a ±10-ppm mass accuracy threshold and allowable cleavages at glutamates and asparagines. A percolator was used to filter the peptide spectral matches to a FDR of less than 1%. All MS/ MS spectra for putative AEP-generated APOA1 cleavage sites were manually inspected (16).

### Immunostaining.

We used free-floating 12 μm aorta sections in immunostaining. For IHC staining, the samples were treated with 0.3% H_2_O_2_ for 10 minutes and washed 3 times in PBS. Then the samples were blocked in 1% BSA and 0.3% Triton X-100, for 30 minutes, followed by overnight incubation with anti-APOA1 N208 (1:300) at 4 °C. The signal was developed using mouse- and rabbit-specific HRP/DAB (ABC) Detection IHC kit (Abcam). For immunofluorescence staining, the sections were incubated overnight at 4°C with primary antibodies listed in Supplemental Table 1. After washing with PBS, the sections were incubated with a mixture of Alexa Fluor 488-, 555- and 647-coupled secondary antibodies (Invitrogen; Supplemental Table 1) for detection. DAPI (1 μg/mL) (Sigma-Aldrich) was used for staining nuclei. Images were acquired with a Zeiss LSM 980 confocal microscope with z series (Carl Zeiss Meditec). ImageJ was used for intensity analysis.

### In vitro APOA1 cleavage assay.

To assess the cleavage of APOA1 by AEP in vitro, HEK293 cells (obtained from ATCC) were transfected with 10 mg GST-APOA1 plasmids by the calcium phosphate precipitation method. Forty-eight hours after transfection, the cells were collected, washed once in PBS, lysed in lysis buffer (50 mM sodium citrate, 5 mM dithiothreitol [DTT], 0.1% CHAPS and 0.5% Triton X-100, pH 7.4), and centrifuged for 10 minutes at 14,000*g* at 4 °C. The supernatant was then incubated with mouse kidney lysates at pH 7.4 or 6.0 at 37 °C for 30 minutes. To measure the cleavage of purified APOA1 fragments by AEP, GST-tagged full-length or fragmented APOA1 were purified with glutathione beads. The purified APOA1 was incubated with recombinant AEP (5 mg/mL) in AEP buffer (50 mM sodium citrate, 5 mM DTT, 0.1% CHAPS and 0.5% Triton X-100, pH 6.0) for 5–30 minutes. The samples were then boiled in SDS loading buffer and analyzed by immunoblotting.

### Generation of the anti-APOA1 N208 antibody that specifically recognizes the AEP-generated APOA1 fragment.

The anti-APOA1 N208 antibody was generated by immunizing rabbits with the peptide Ac-RLAARLEALKEN-OH. The antiserum was pooled and the titers against the immunizing peptide were determined by ELISA. The maximal dilution giving a positive response with the chromogenic substrate for horseradish peroxidase was 1:512,000. The immunoactivity of the antiserum was further confirmed by Western blotting and IHC.

### Cholesterol efflux.

Raw264.7 cell line was obtained from the Cell Bank of Chinese Academy of Sciences (Shanghai, China). Raw264.7 cells were cultured in RPMI 1640 supplemented with 10% heat-inactivated FBS, L-glutamine, and penicillin/streptomycin. Cholesterol efflux was determined as described elsewhere (56). Briefly, Raw264.7 cells were incubated with NBD-cholesterol for 4 hours. After cholesterol loading, the cells were washed, equilibrated for 2 hours and incubated for 4 hours with DMEM containing no phenol red (Gibco, 21063) with 0.2% BSA, APOA1-His, APOA1 N208A-His, APOA1 1-208-His, or APOA1 209-276-His. Wells with only 0.2% BSA were set as control to measure the background. The medium and cells were collected. Then the cells were lysed with 0.3 M NaOH solution for 15 minutes at 37°C. The fluorescence intensity of medium and cell lysates were measured with a microplate spectrophotometer. The efflux rate was calculated as follows: Efflux (%) = medium counts: (medium counts + cell lysate counts) × 100.

### Lipid clearance assay.

DMPC assay was used to determine the ability of APOA1 to bind and solubilize lipid (44, 57). In brief, APOA1-His, ApoA 1 N208A-His, APOA1 1-208-His, and APOA1 209-276-His were diluted to 0.5 mg/mL. DMPC multilamellar vesicles (0.5 mg/mL) were mixed with each APOA1 preparation in Tris reaction buffer (10 mM Tris, 8.5% KBr, 0.01% NaN3, 0.01% EDTA-Na2, pH 7.4) or with reaction buffer alone (control) in a 2.5:1 (v:v) ratio and incubated at 24°C. DMPC clearance was measured after the decrease in absorbance at 325 nm at 2 minute intervals for up to 60 minutes. Results were plotted as the absorbance at 325 nm at each time point over the initial absorbance at 0 minutes (OD/OD_0_).

### AAV infection.

The Adeno-associated virus (AAV) particles encoding full-length APOA1 and APOA1 N208 with the TBG promoter were prepared by Shumi Technologies. AAV carrying mCherry, APOA1, or APOA1 N208A cDNAs under the control of TBG promoter was injected into APOE^–/–^ mice through the tail vein at a dose of 1 × 10^11^ virus genome at 6-weeks old.

### Assessing AEP activity in vivo.

Mice were injected with LE28 (10 nmol in 20% DMSO/PBS, approximately 2 mg/kg) by tail vein. Mice were anesthetized with isoflurane and then imaged 6 hours after injection using an IVIS 100 system.

### Statistics.

Statistical analyses were performed using GraphPad Prism. After the Shapiro-Wilk test of normality, the statistical difference with normal distribution and homogeneous variance was examined by unpaired Student’s *t* test (2-group comparison) or 1-way ANOVA (more than 2 groups), otherwise, it was tested with the Kruskal-Wallis test (more than 2 groups) (quantification of C/EBP-β in Figure 1E and Figure 1I). Differences with *P* values less than 0.05 were considered significant.

### Study approval.

All animal experiments were conducted in accordance with the guidelines and approved by the Institutional Animal Care and Use Committee (IACUC) of the Shenzhen Institutes of Advanced Technology, Chinese Academy of Sciences (Approval No. [SIAT-IACUC-240409-NS-WMM-A2585]). For human experiments, ethics approval was obtained from the collection sites (Guangzhou Science and Technology Program key projects [202002020037]). Patients with severe liver and kidney dysfunction, malignant tumors, other infectious or immune diseases, and communication or cognitive dysfunction were excluded.

### Data availability.

Data are available in the Supporting Data Values file.

## Author contributions

KY conceived the project, designed the experiments, analyzed the data, and wrote the manuscript. MW and XY designed the experiments. MW performed most of the experiments. BL and SN performed the AEP cleavage in vitro. GW performed the virus injection. XM performed animal experiments. MY, WD, and KH assisted with data analysis and interpretation and critically read the manuscript. TS provided the human tissue samples. PX performed the LC-MS/MS experiments.

## Supplementary Material

Supplemental data

Unedited blot and gel images

Supporting data values

## Version 1 05/15/2025

Electronic publication
