# Supplementary material for: Asparagine endopeptidase cleaves apolipoprotein A1 and accelerates pathogenesis of atherosclerosis
Source: J Clin Invest. 2025 May 15;135(10):e185128. doi: 10.1172/JCI185128 (PMC12077905; doi:10.1172/JCI185128)
Supplement: Supplemental data [file jci-135-185128-s008.pdf]

Supplementary Materials

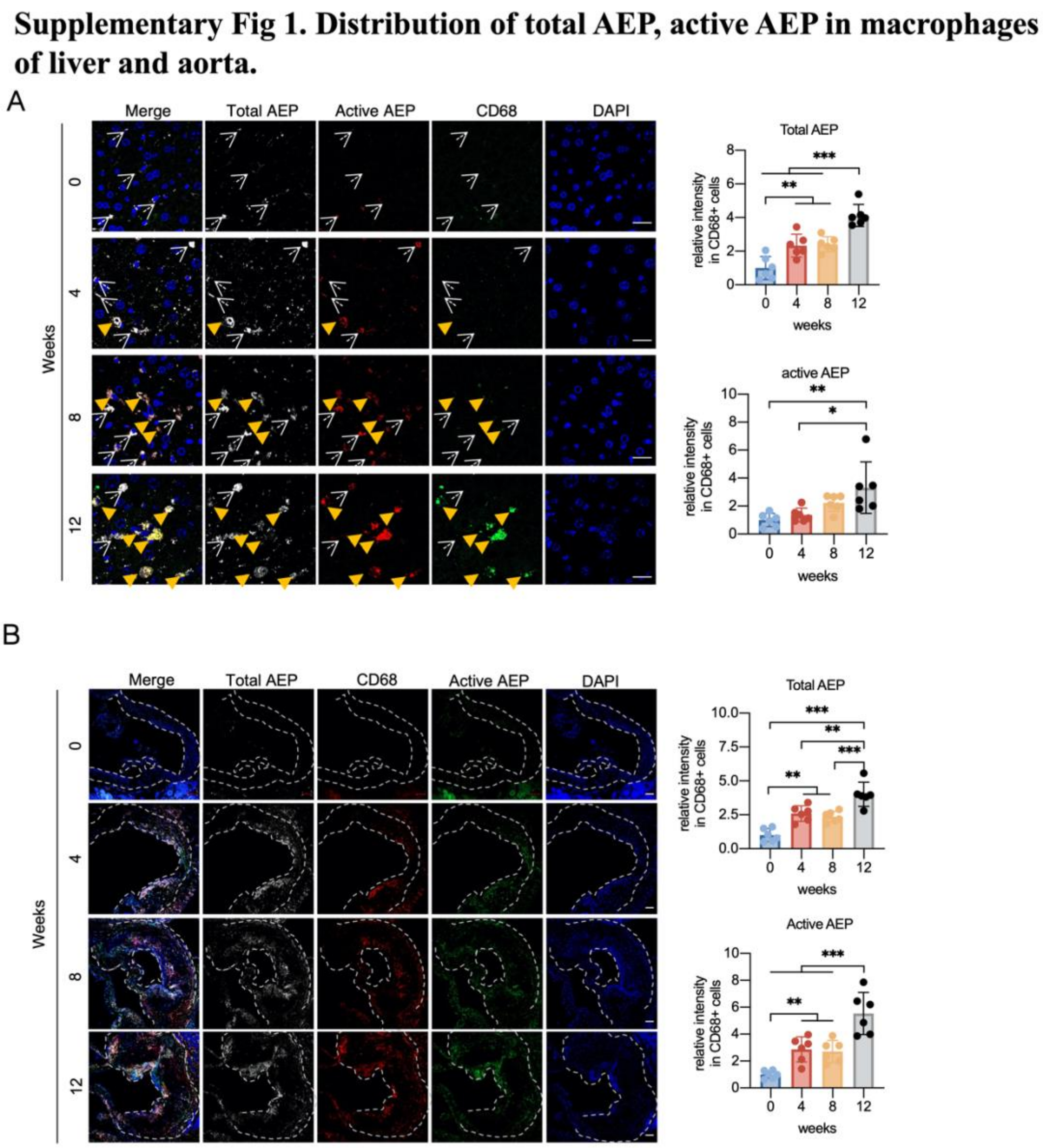

Supplementary Figure 1. Distribution of total AEP, active AEP in macrophages of liver and aorta.

A, Immunofluorescence staining and quantification of CD68 (green), active AEP (red) and total AEP (white) in liver of APOE-/- mice fed with HFD for 0,4,8 or 12 weeks. Nuclei were counterstained with DAPI (blue; scale bars, 20 μm). **B,** Immunofluorescence staining and quantification of CD68 (green), active AEP (red) and total AEP (white) in aorta of APOE-/- mice fed with HFD for 0,4,8 or 12 weeks. Nuclei were counterstained with DAPI (blue; scale bars, 20 μm). One-way ANOVA with Tukey's post-hoc test (**A** and **B**). *, $P < 0.05$; **, $P < 0.01$; ***, $P < 0.001$.

Supplementary Fig 2. Depletion of AEP from APOE-/- mice inhibits the development of atherosclerosis.

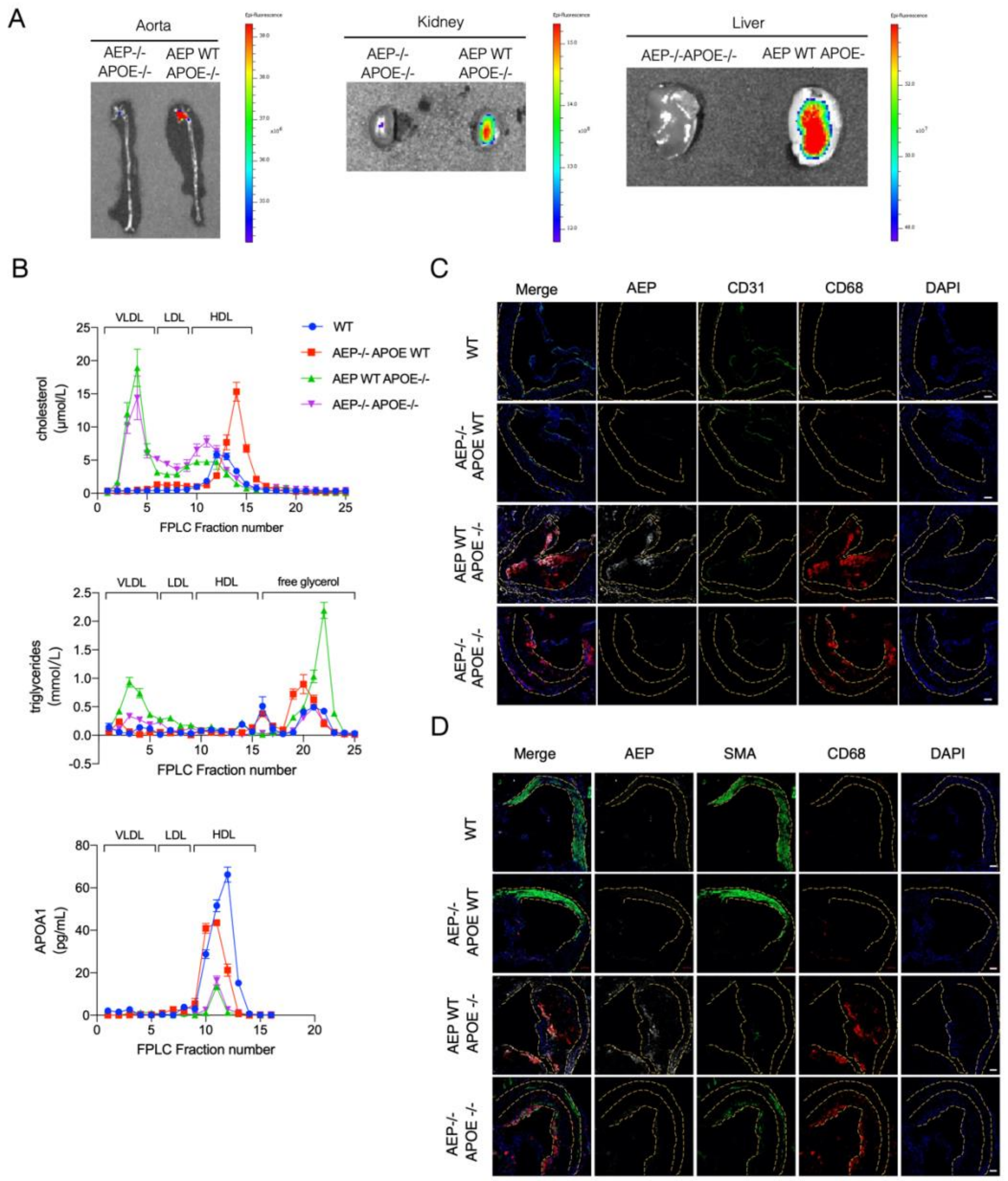

Supplementary Figure 2. Depletion of AEP from APOE-/- mice inhibits the development of ATH.

A, Imaging of AEP-/-/APOE-/- and APOE-/- mice by using IVIS 100 after injection with LE28. **B**, Cholesterol, triglycerides and APOA1 levels in each SEC-separated plasma fractions from WT, AEP-/-/APOEWT, AEP WT/APOE-/- and AEP-/-APOE-/-mice. **C**, Immunofluorescence staining and quantification of CD68 (red), CD31 (green) and total AEP (white) in aorta. **D**, Immunofluorescence staining and quantification of CD68 (red), SMA (green) and total AEP (white) in aorta.

Supplementary Figure 3. Antibody specifically recognized the APOA1 (1-208) fragment

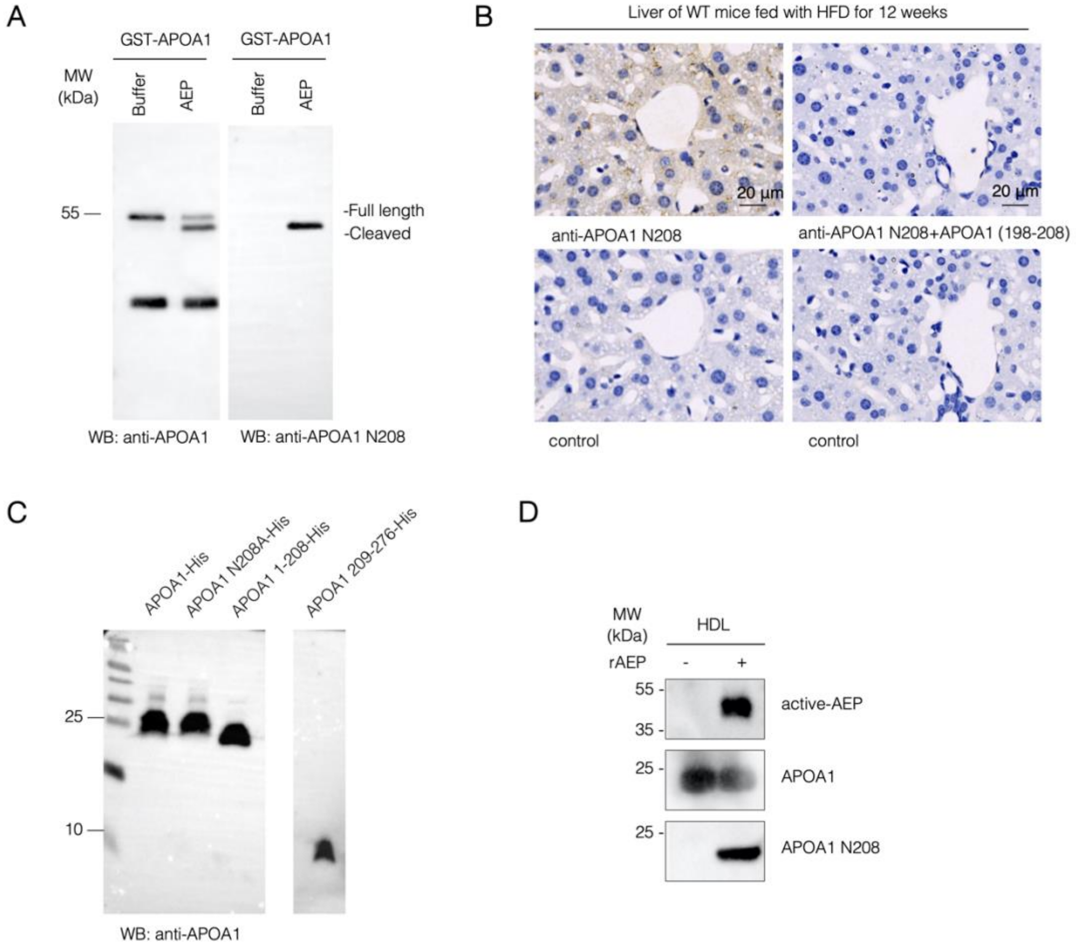

Supplementary Figure 3. Antibody specifically recognized the APOA1 (1-208) fragment

A, Western blot showed the specificity of anti-APOA1 N208 antibody. GST-APOA1 was incubated with buffer or AEP for 10 minutes, respectively. Anti-APOA1 N208 antibody only recognized APOA1 (1-208) fragments and did not recognize the entire length of APOA1. **B**, IF showed the specificity of anti-APOA1 N208 antibody. IF was performed on mouse liver tissue using anti-APOA1 N208 antibody with or without APOA1 (198-208) for blocking. APOA1 (198-208) peptide completely blocked the positive signal of anti-APOA1 N208 antibody. Bar, 20 μm. **C**, Purification of APOA1-His, APOA1 N208-His, APOA1 1-208-His and APOA1 209-276-His recombinant proteins. **D**, Western blot showed the cleavage of APOA1-HDL by AEP. HDL was incubated with buffer or AEP for 30 minutes, respectively. Anti-APOA1 N208 antibody only recognized APOA1 (1-208) fragments and did not recognize the entire length of APOA1.

Supplementary Fig 4. Mutation of APOA1 blocked the cleavage of APOA1.

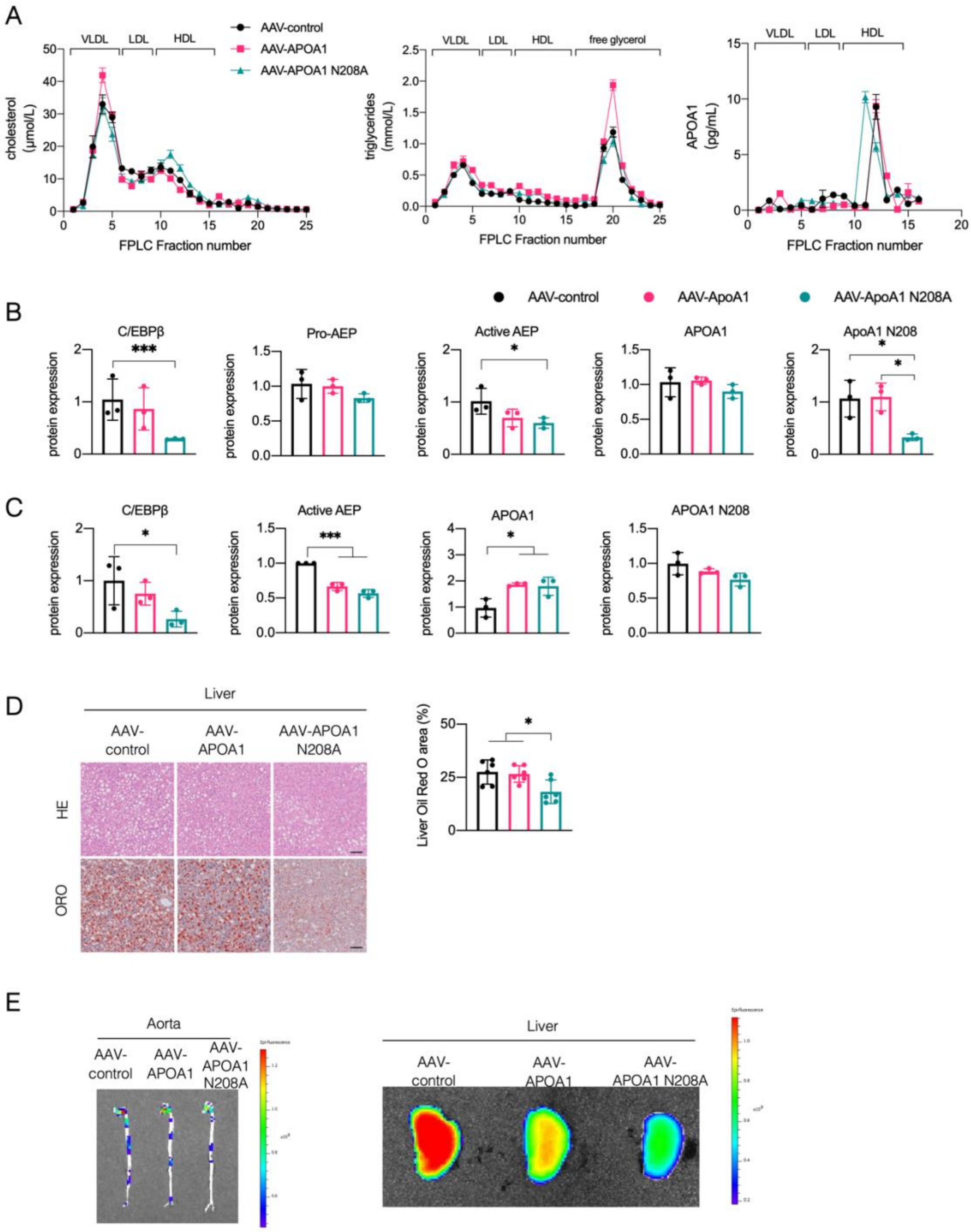

Supplementary Figure 4. Mutation of APOA1 blocked the cleavage of APOA1.
A, Cholesterol, triglycerides and APOA1 levels in each SEC-separated plasma fractions from AAV-control, AAV-APOA1 and AAV-APOA1 N208A. **B and C,** Western blot quantification of C/EBPβ, AEP, APOA1 and APOA1 N208 levels in aorta (A) and liver (B), n=3 per group. **D**, H&E and ORO staining of liver. **E**, Imaging of AEP-/-APOE-/- and APOE-/- mice by using IVIS 100 after injection with LE28. All data are presented as the mean ± SEM from 3 to 6

independent experiments. One-way ANOVA with Tukey's post-hoc test (**B**, **C** and **D**). *, $P < 0.05$; **, $P < 0.01$; ***, $P < 0.001$.

Supplementary Fig 5. #11a inhibits AEP activity in APOE-/- mouse model.

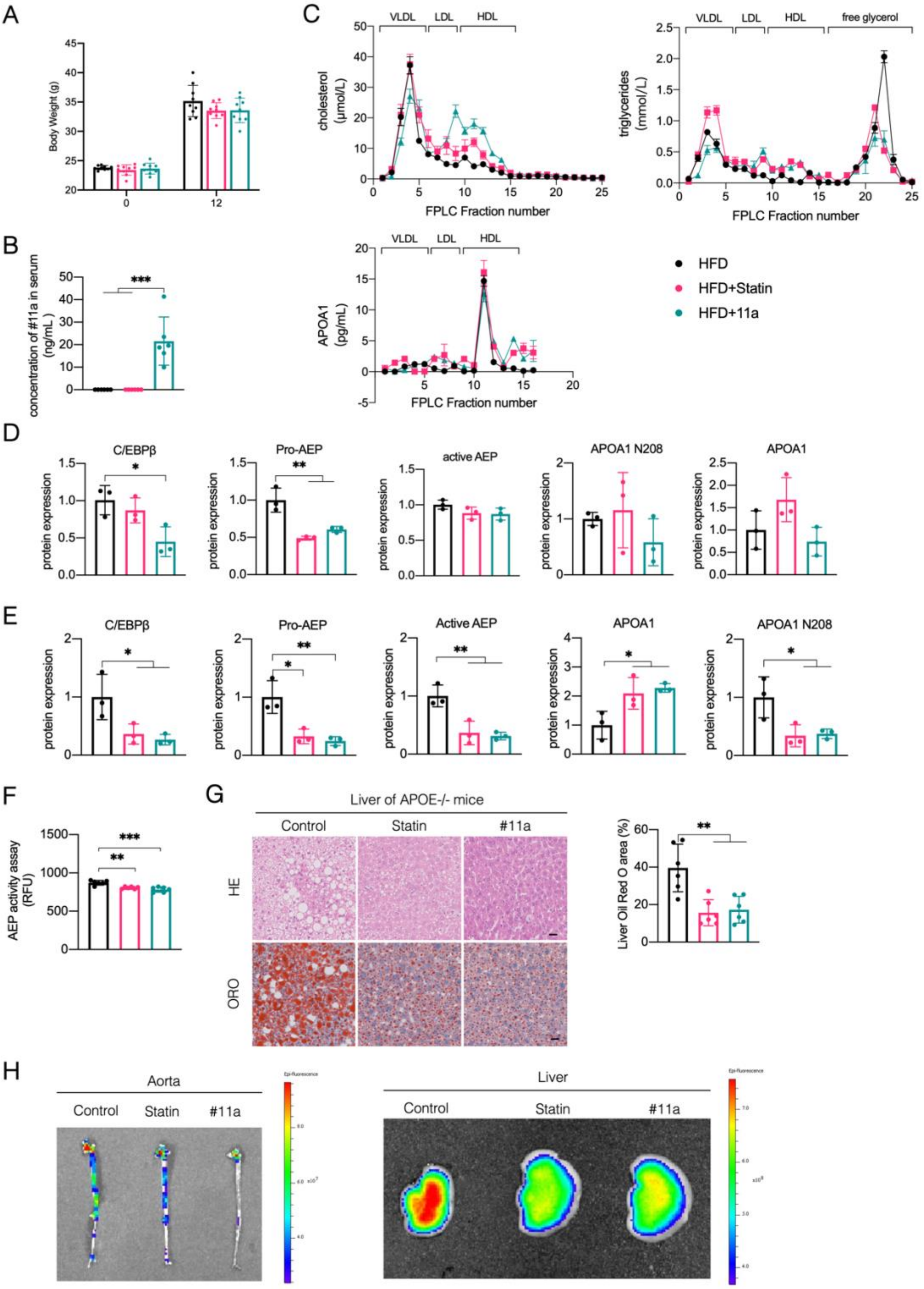

Supplementary Fig 5. #11a inhibits AEP activity in APOE-/- mouse model.

A, Body weight of APOE-/- mice fed with HFD, HFD+Statin and HFD+#11a at 0 and 12 weeks. **B,** serum levels of #11a. **C**, Cholesterol, triglycerides and APOA1 levels in each SEC-separated plasma fractions from APOE-/- mice fed with HFD, HFD+Statin and HFD+#11a. **D & E**,

Western blot quantification of C/EBPβ, AEP, APOA1 and APOA1 N208 levels in aorta (B) and liver (C), n=3 per group. **F**, AEP enzymatic activities of liver (n=6 per group). **G**, H&E and ORO staining of liver. **H**, Imaging of APOE-/- mice fed with Western diet, Western diet + statin or Western diet + #11a by using IVIS 100 after injection with LE28. All data are presented as the mean ± SEM from 3 to 6 independent experiments. One-way ANOVA with Tukey's post-hoc test (**A**, **B**, **D**, **E**, **F** and **G**).*, $P < 0.05$; **, $P < 0.01$; ***, $P < 0.001$.

Supplementary Fig 6. #11a did not further inhibits atherosclerosis development in AEP-/-APOE-/- mouse model.

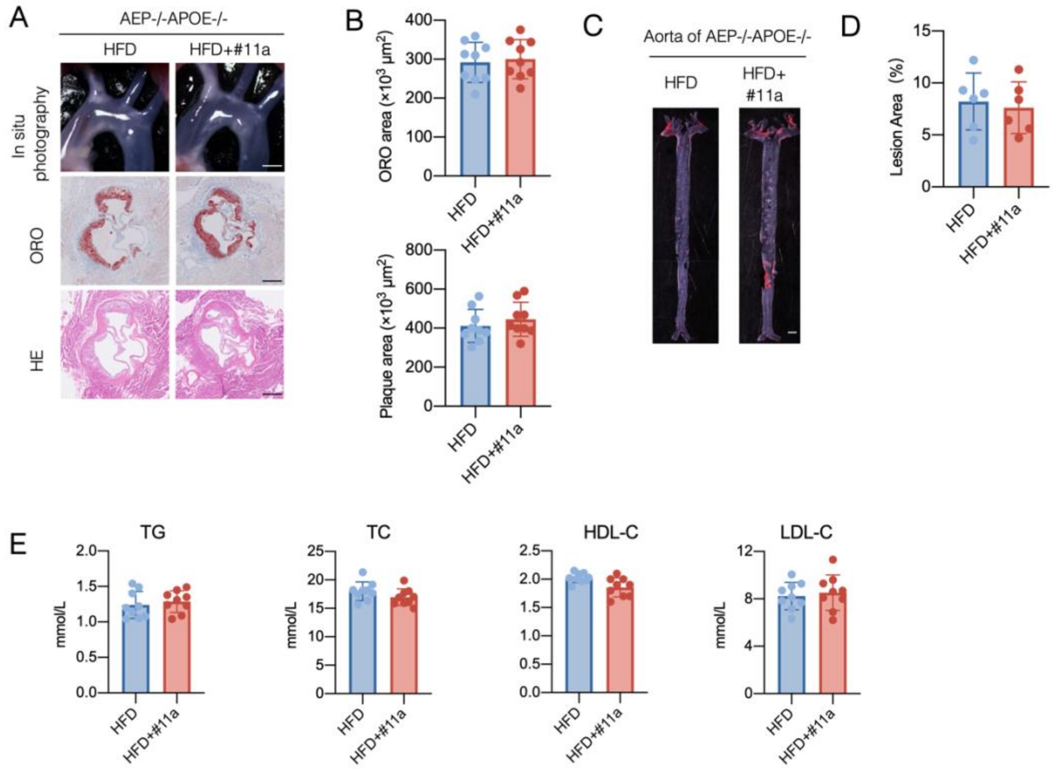

Supplementary Figure 6. #11a did not further inhibits ATH development in AEP-/-APOE-/- mouse model.

A through **E**, AEP-/-APOE-/- mice were fed with high-fat diet (control) or high-fat diet + #11a (#11a, 75 mg/kg) for 12 weeks from 8-week-old. **A**, Representative macroscopic images of aortic arch (scale bar, 1 mm) and aortic root stained with hematoxylin and eosin (HE) and Oil Red O (ORO; scale bars, 25 μm). **B**, Quantification of aortic plaque and ORO area in aortic root (n=9 per group). **C & D**, Representative macrographs of aorta stained with Oil Red O (scale bar, 1 mm) (n=6 per group). **E**, Serum levels of TC, TG, LDL-C and HDL-C (n=9 per group). All data are presented as the mean ± SEM from 9 independent experiments. Two-tailed, unpaired *t* test (**B, D** and **E**). *, $P < 0.05$; **, $P < 0.01$; ***, $P < 0.001$.

Supplementary Fig 7. #11a inhibits AEP activity and attenuates atherosclerosis in LDLR-/- mouse model.

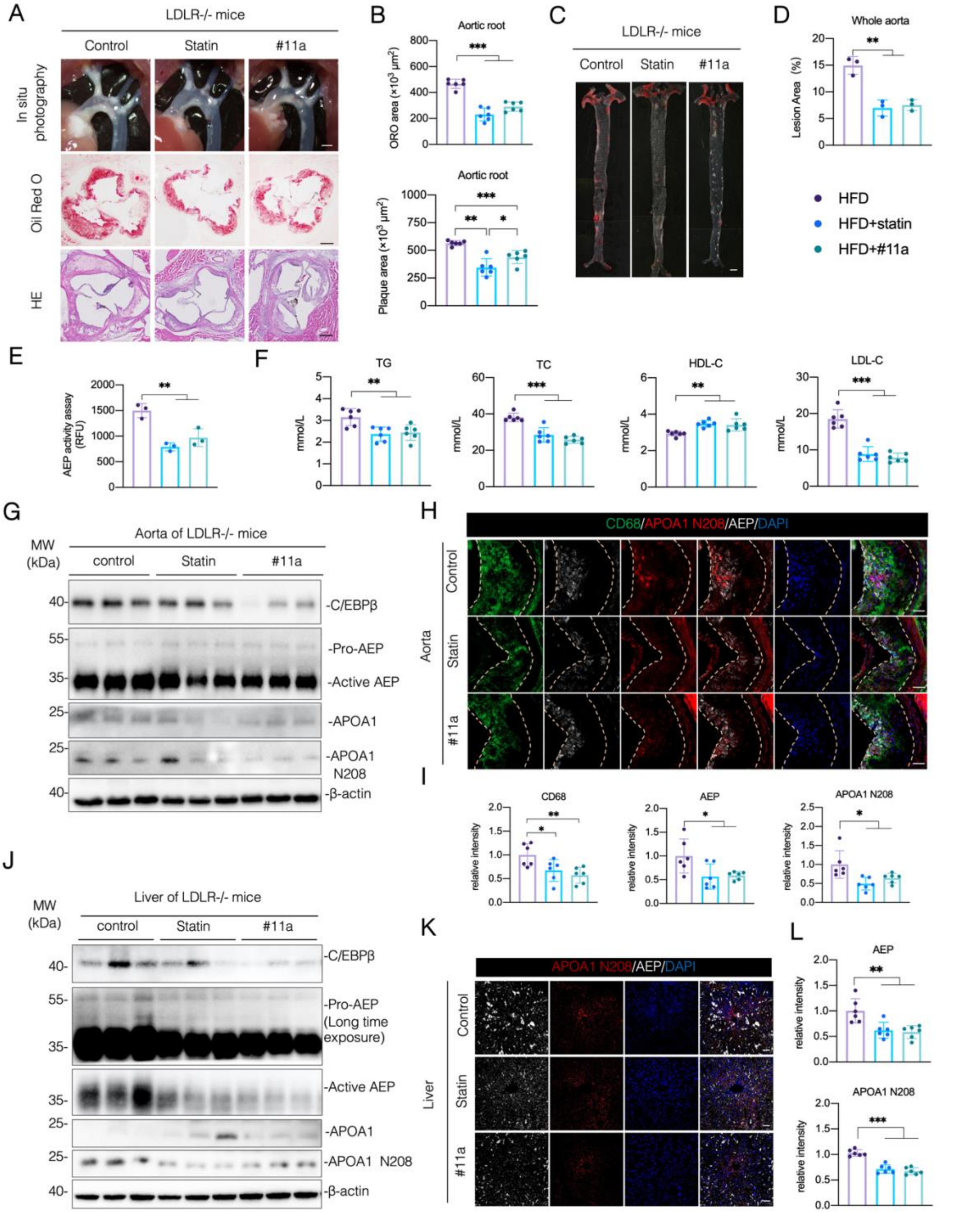

Supplementary Figure 7. #11a inhibits AEP activity and attenuates atherosclerosis in LDLR-/- mouse model.

A through **L**, LDLR$^{-/-}$ mice were fed with high-fat diet (control), high-fat diet + statin (Statin, 100 mg/kg) or high-fat diet + #11a (#11a, 75 mg/kg) for 12 weeks from 8-week-old. **A**,

Representative macroscopic images of aortic arch (scale bar, 1 mm) and aortic root stained with hematoxylin and eosin (HE) and Oil Red O (ORO; scale bars, 25 μm). **B**, Quantification of aortic plaque and ORO area in aortic root (n=6 per group). **C & D**, Representative macrographs of aorta stained with Oil Red O (scale bar, 1 mm) (n=3 per group). **E**, AEP enzymatic activities of aorta (n=3 per group). **F**, Serum levels of TC, TG, LDL-C and HDL-C (n=6 per group). **G**, Western blot analysis of C/EBPβ, AEP, APOA1 and APOA1 N208 levels in aorta (n=3 per group). **H & I**, Immunofluorescence staining and quantification of CD68 (green), AEP (white) and APOA1 N208 (red) in aorta. Nuclei were counterstained with DAPI (blue; scale bars, 20 μm). **J**, Western blot analysis of C/EBPβ, AEP, APOA1 and APOA1 N208 levels in liver (n=3 per group). **K & L**, Immunofluorescence staining and quantification of AEP (white) and APOA1 N208 (red) in liver. Nuclei were counterstained with DAPI (blue; scale bars, 20 μm). All data are presented as the mean ± SEM from 3 to 6 independent experiments. One-way ANOVA with Tukey's post-hoc test (**B**, **D**, **E**, **F**, **I** and **L**). *, $P < 0.05$; **, $P < 0.01$; ***, $P < 0.001$.

Supplementary Fig 8. #11a inhibits AEP activity of LDLR-/- mouse model.

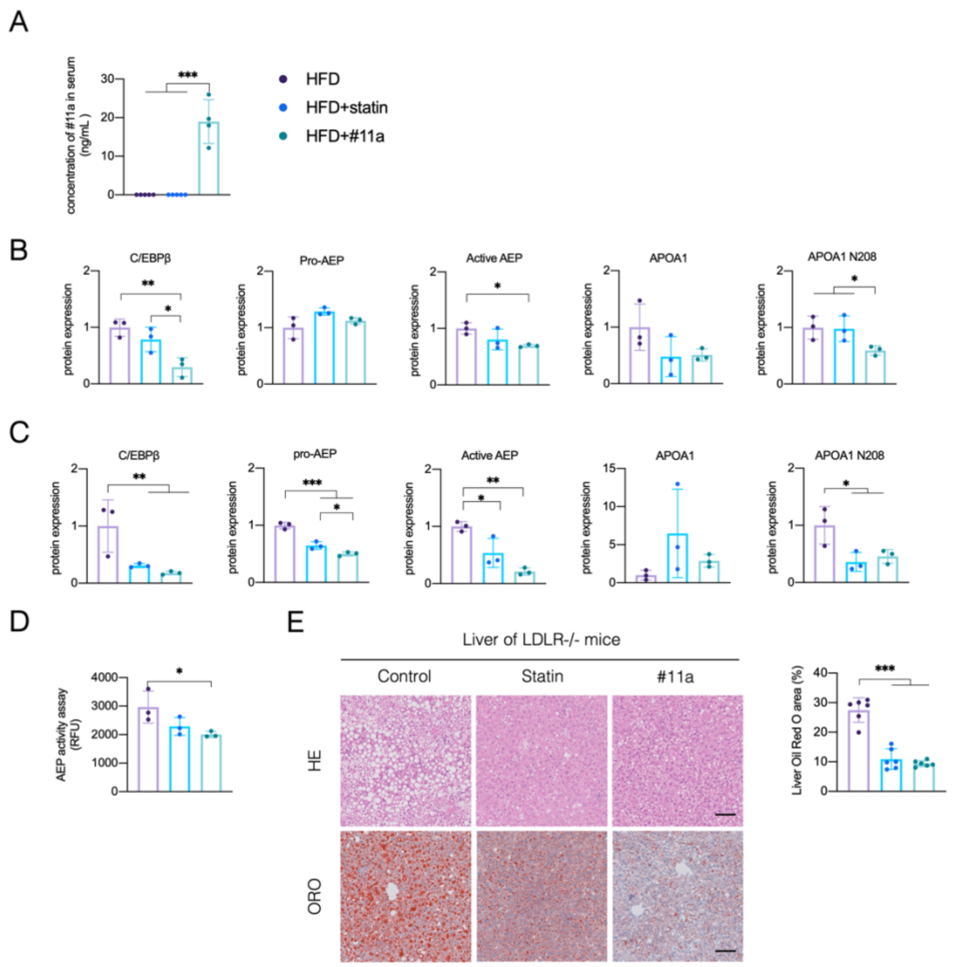

Supplementary Fig 8. #11a inhibits AEP activity in LDLR-/- mouse model.
A, serum level of #11a. **B & C**, Western blot quantification of C/EBPβ, AEP, APOA1 and APOA1 N208 levels in aorta (B) and liver (C), n=3 per group. **D**, AEP enzymatic activities of liver (n=6 per group). **E**, H&E and ORO staining of liver. All data are presented as the mean ± SEM from 3 to 6 independent experiments. One-way ANOVA with Tukey's post-hoc test (**A**, **B**, **C**, **D** and **E**). *, $P < 0.05$; **, $P < 0.01$; ***, $P < 0.001$.

99 **Supplementary Table I. Antibodies**

Antibody	Vendor name	Catalog number	Dilution	Application
AEP	Cell Signaling Technology	93627	1:1000	WB
AEP	R&D systems	AF2058	1:200	IF
CD68	Abcam	ab213363	1:200	IF
β-actin	Sigma Aldrich	A5316	1:5000	WB
GAPDH	ProteinTech	60004	1:10000	WB
APOA1	Signalway Antibody	38197	1:100, 1:1000	IF, WB
C/EBPβ	Santa Cruz	7962	1:100	WB
APOA1 N208	GenScript ProBio	-	1:300, 1:3000	IF, WB
APOE	Millipore Sigma	AB947	1:1000	WB
α-SMA	Affinity	AF1032	1:300	IF
CD31	R&D systems	AF3628	1:100	IF
Alexa FluorTM 488 donkey anti-mouse IgG (H+L)	Invitrogen	A21202	1:500	IF
Alexa FluorTM 555 donkey donkey anti-rabbit IgG (H+L)	Invitrogen	A31572	1:500	IF
Alexa FluorTM 647 donkey donkey anti-sheep IgG (H+L)	Invitrogen	A21448	1:500	IF

100 IF: immunofluorescence; WB: western blot.
