# Supplementary material for: Asparagine endopeptidase cleaves apolipoprotein A1 and accelerates pathogenesis of atherosclerosis
Source: J Clin Invest. 2025 May 15;135(10):e185128. doi: 10.1172/JCI185128 (PMC12077905; doi:10.1172/JCI185128)

Full unedited blot/gel for Figure 1D

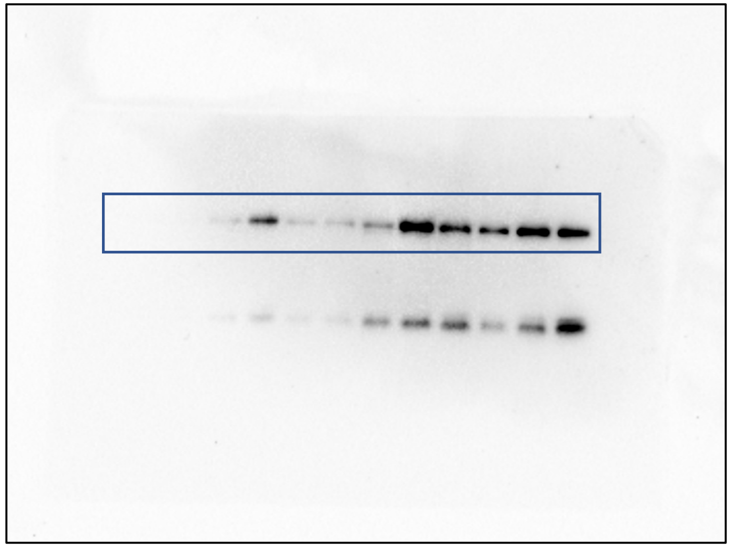

Anti-C/EBPβ

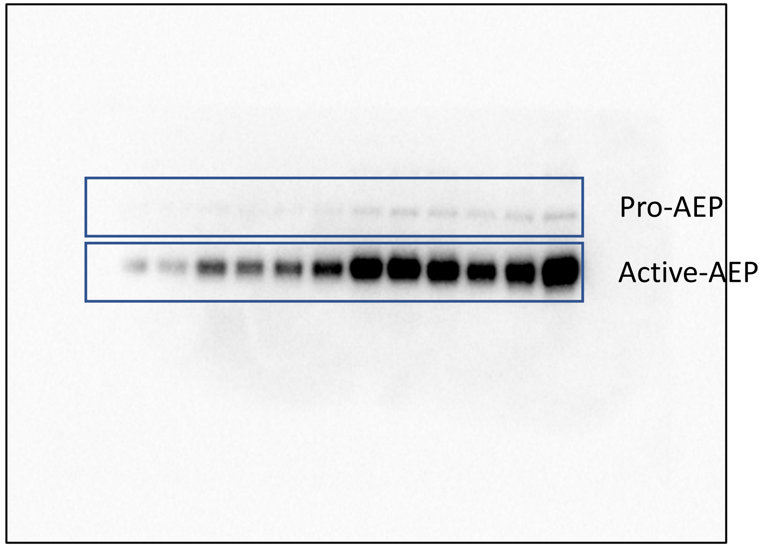

Anti-AEP

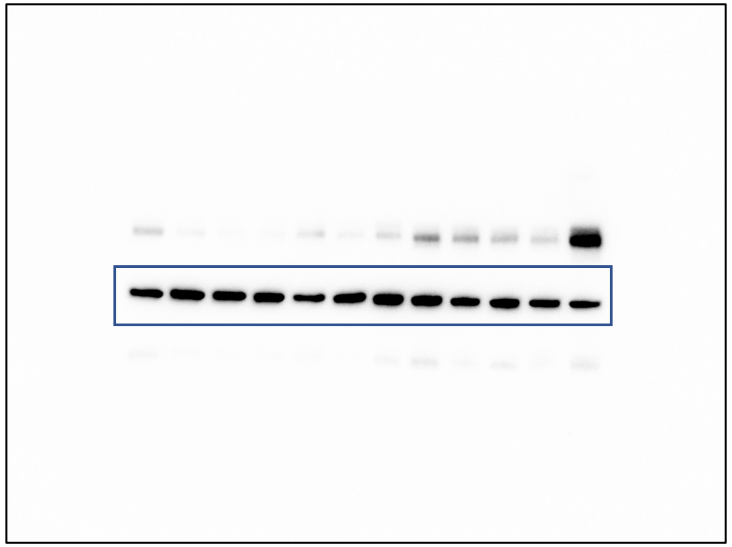

Anti-GAPDH

Full unedited blot/gel for Figure 1H

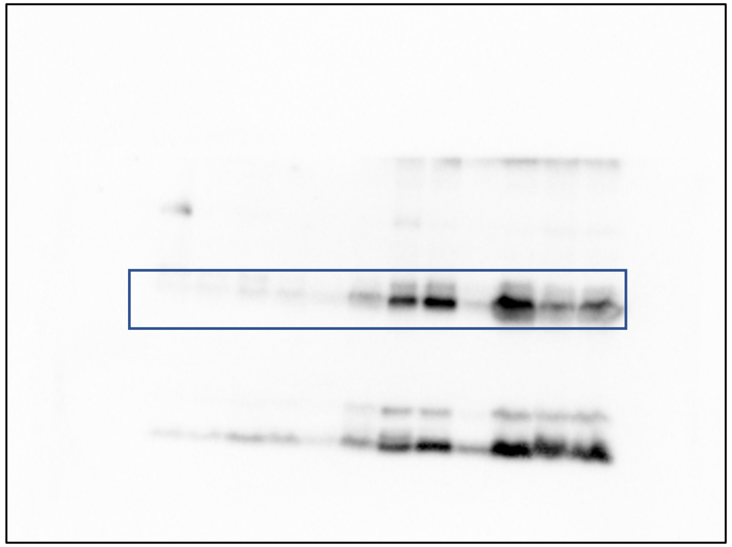

Anti-C/EBPβ

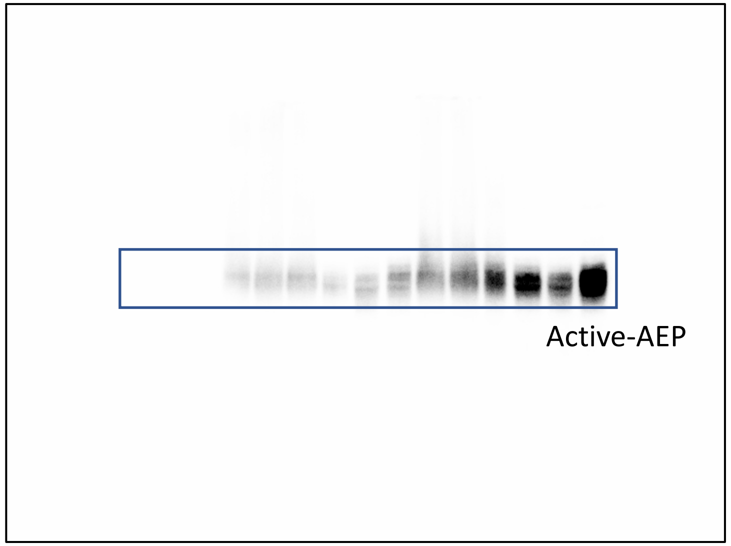

Anti-AEP

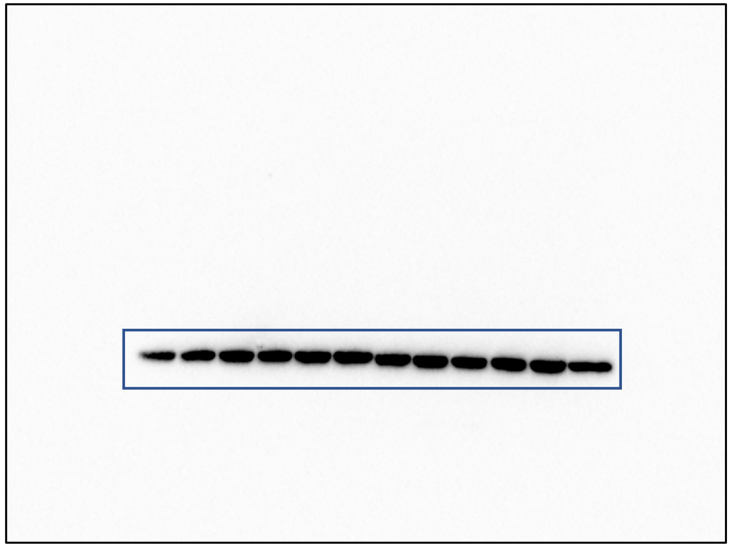

Anti-GAPDH

Full unedited blot/gel for Figure 2G

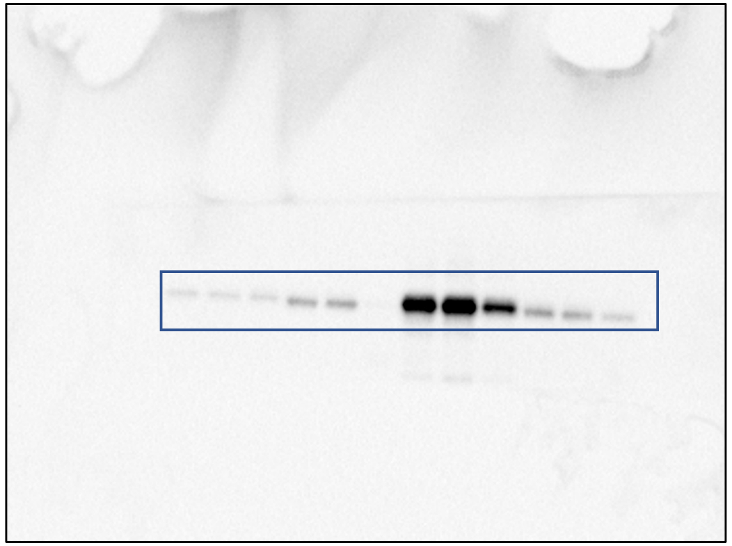

Anti-C/EBPβ

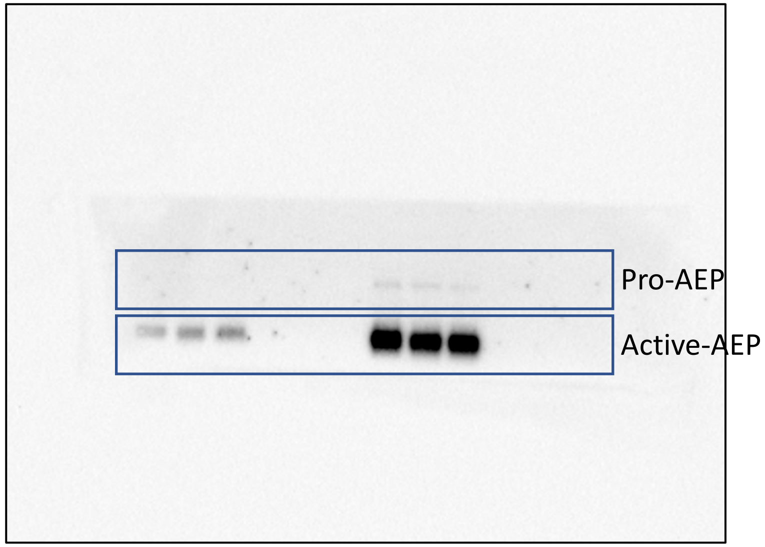

Anti-AEP

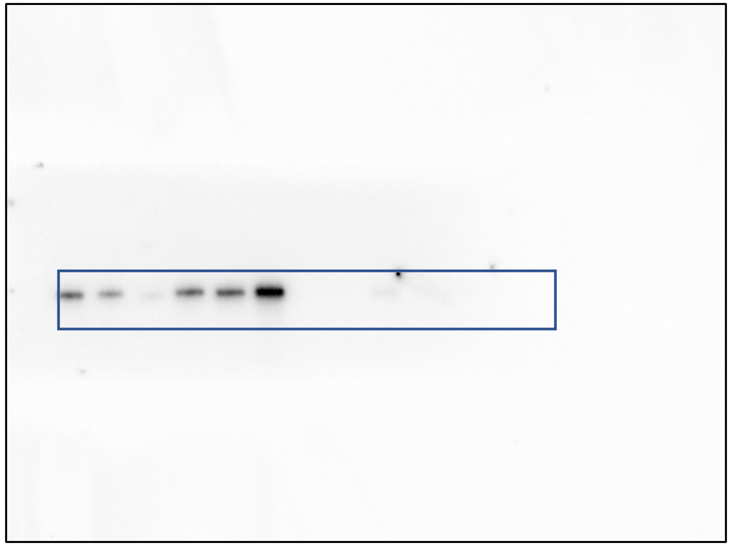

Anti-APOE

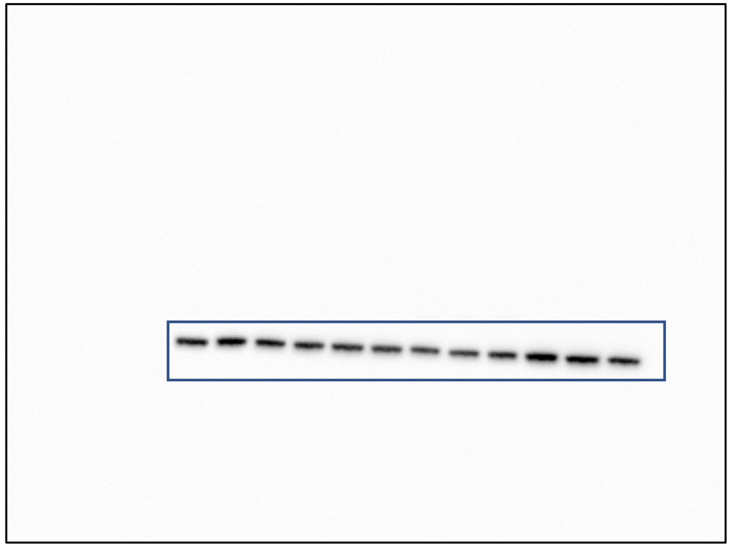

Anti-GAPDH

Full unedited blot/gel for Figure 3A

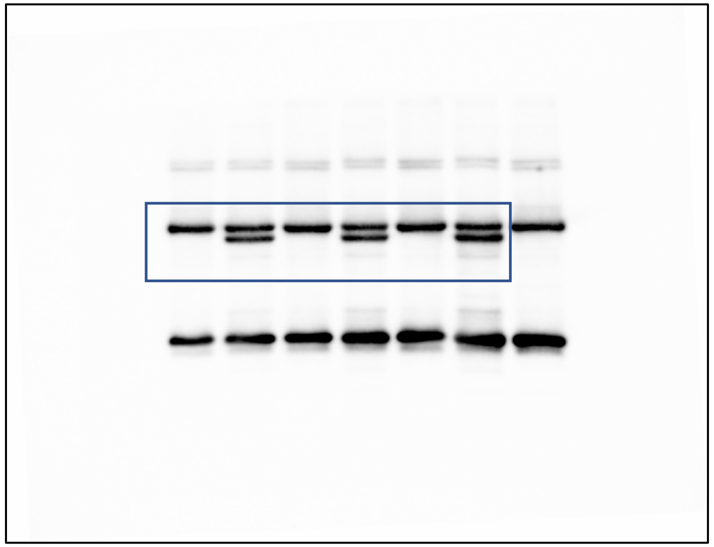

Anti-APOA1

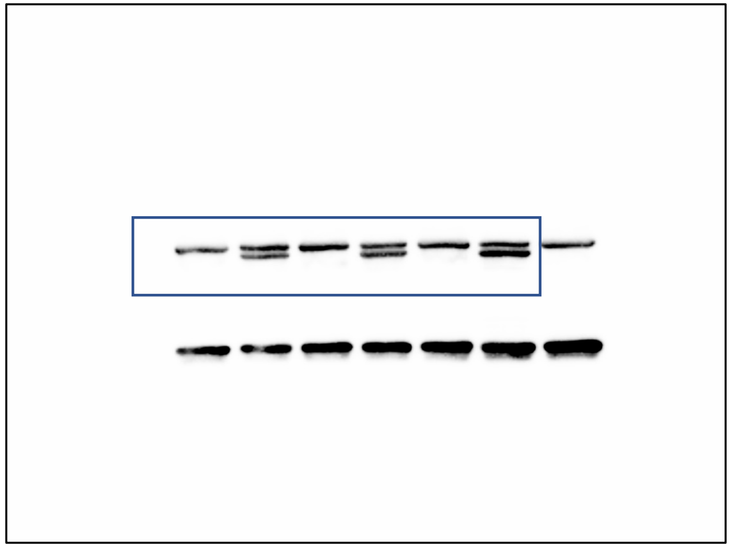

Anti-GST

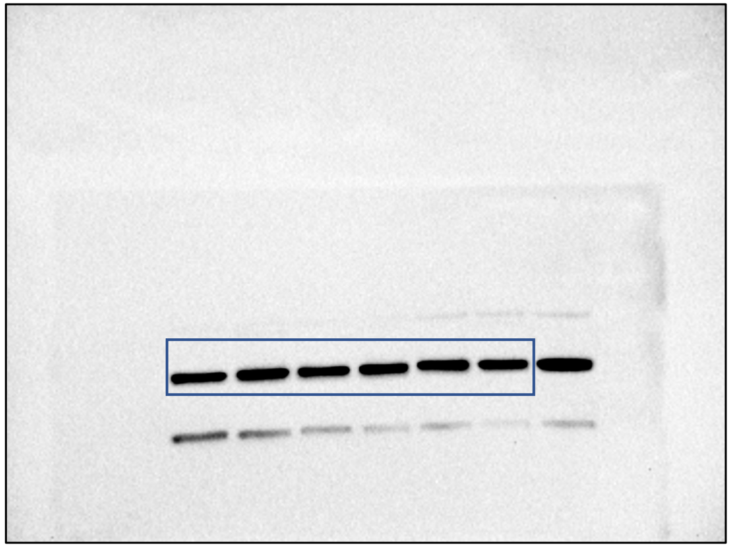

Anti-GAPDH

Full unedited blot/gel for Figure 3B

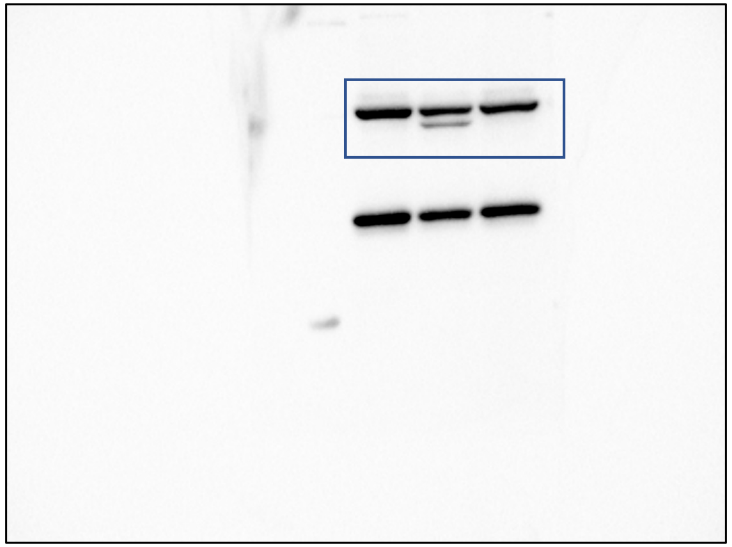

Anti-APOA1

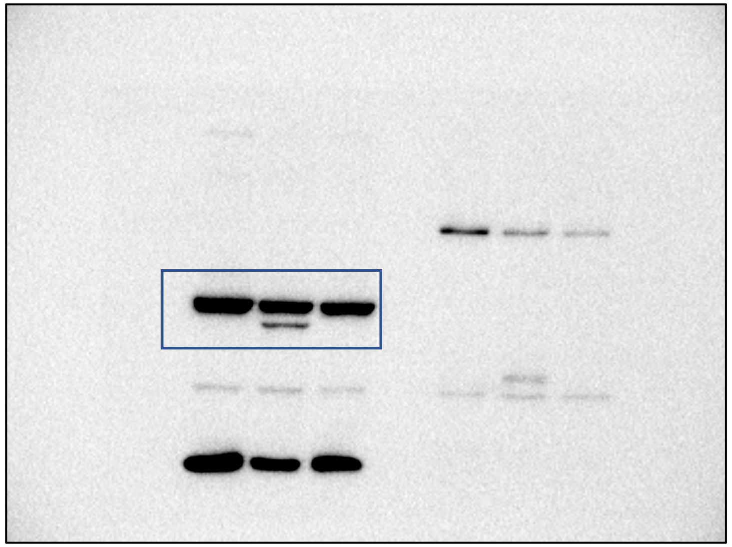

Anti-GST

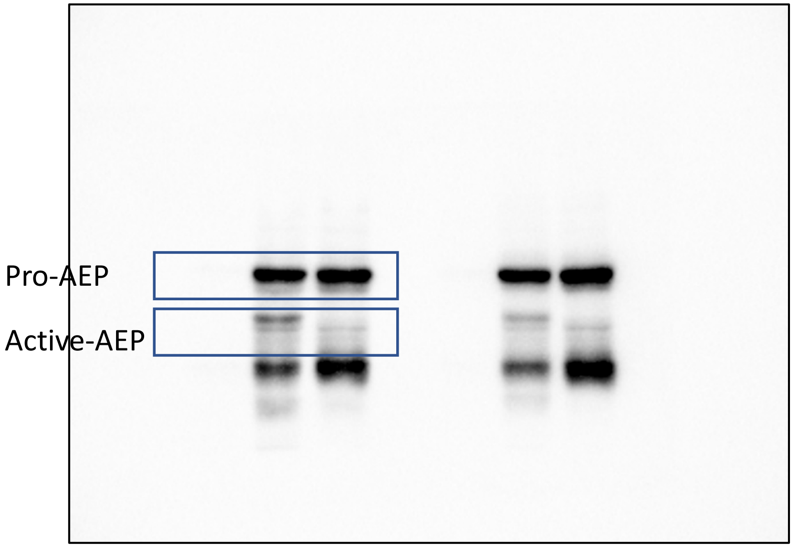

Anti-AEP

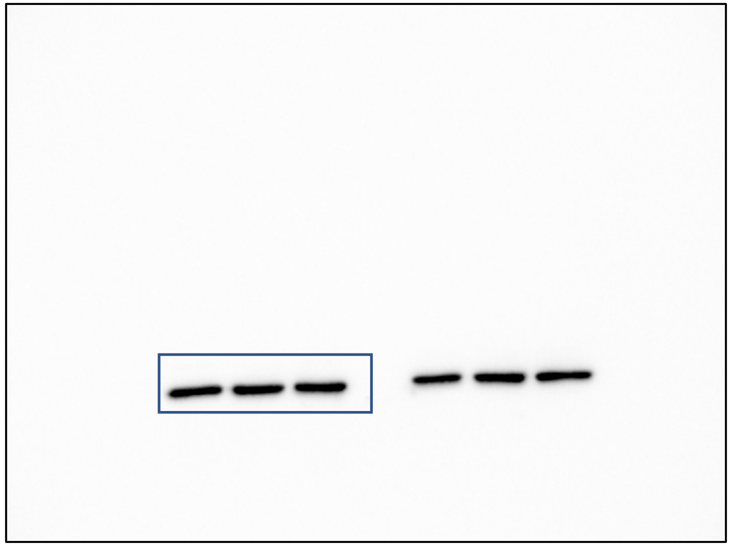

Anti-GAPDH

Full unedited blot/gel for Figure 3E

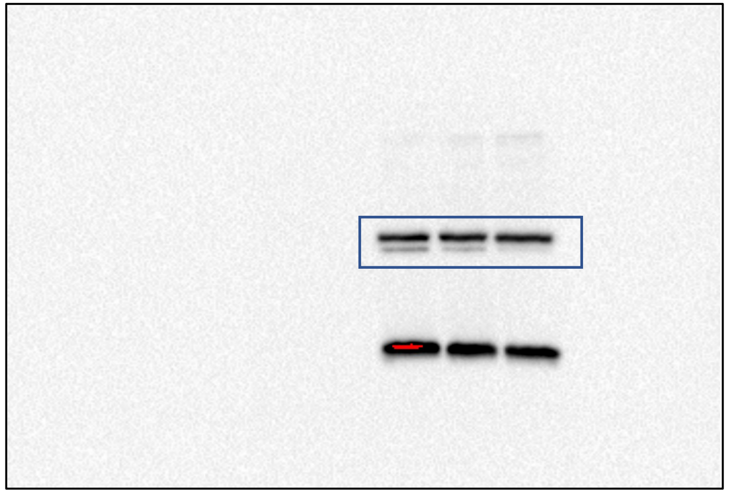

Anti-APOA1

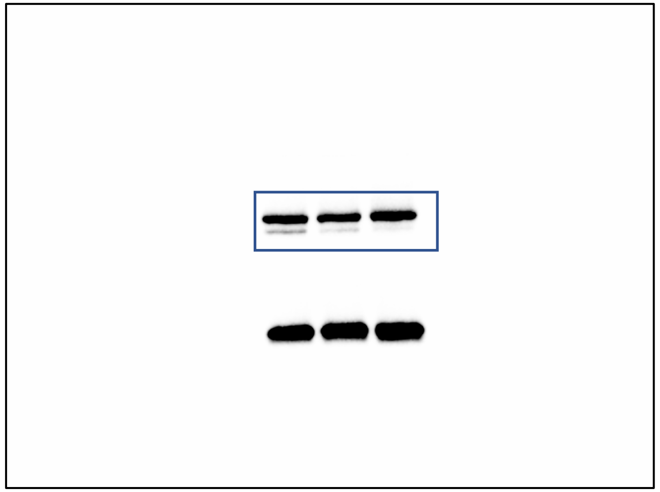

Anti-GST

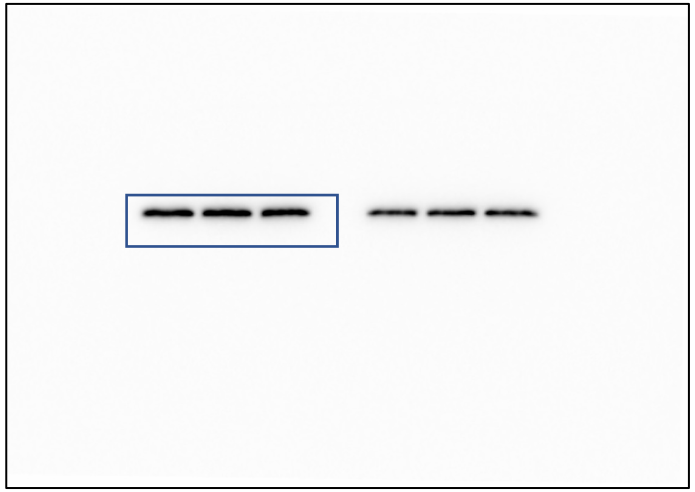

Anti-GAPDH

Full unedited blot/gel for Figure 3F

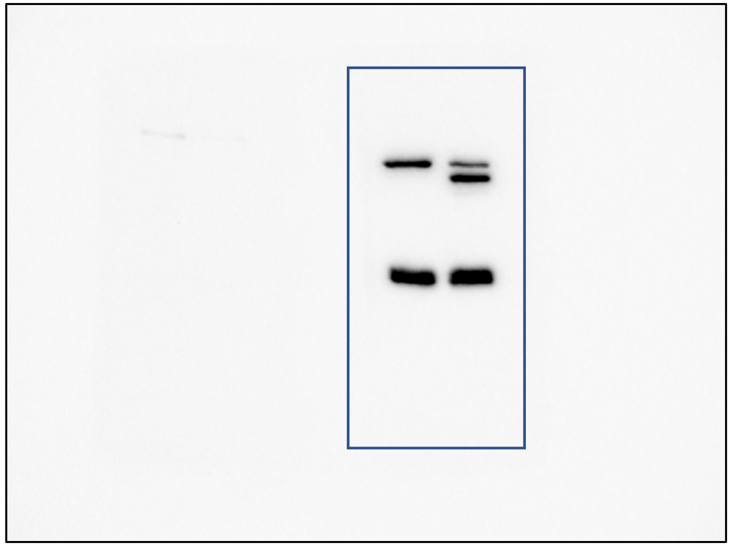

Anti-GST

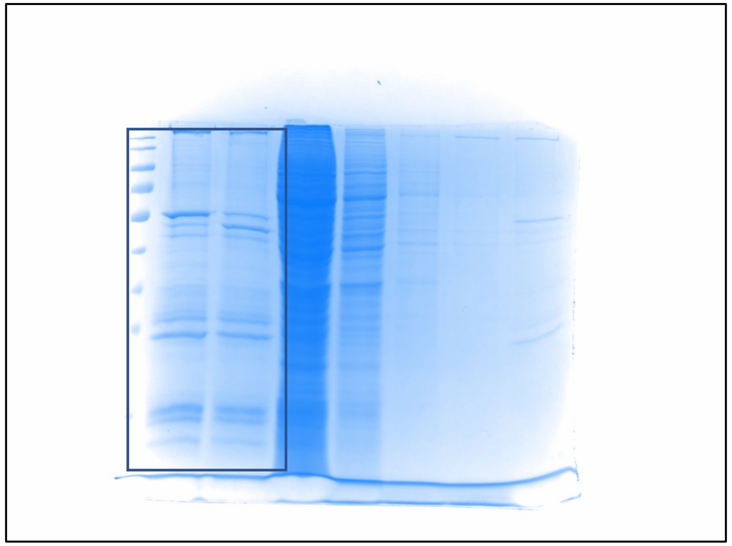

Coomassie Blue

Full unedited blot/gel for Figure 3H

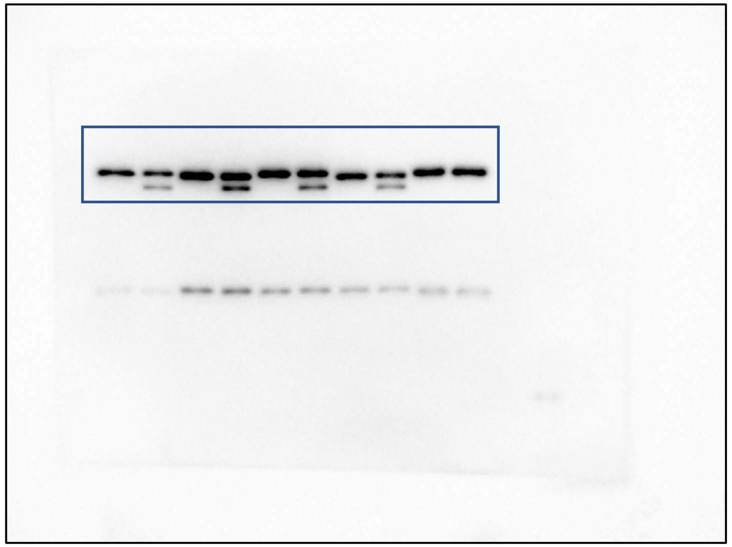

Anti-APOA1

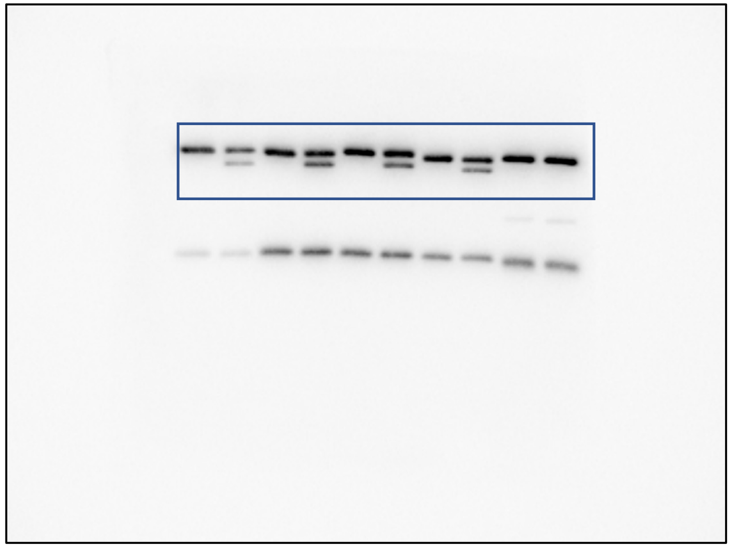

Anti-GST

Full unedited blot/gel for Figure 4A

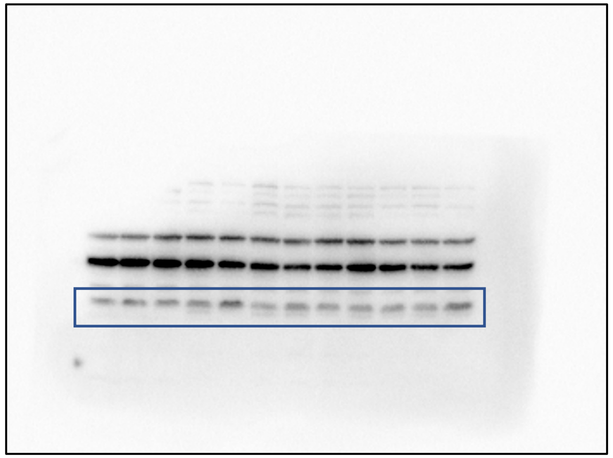

Anti-APOA1

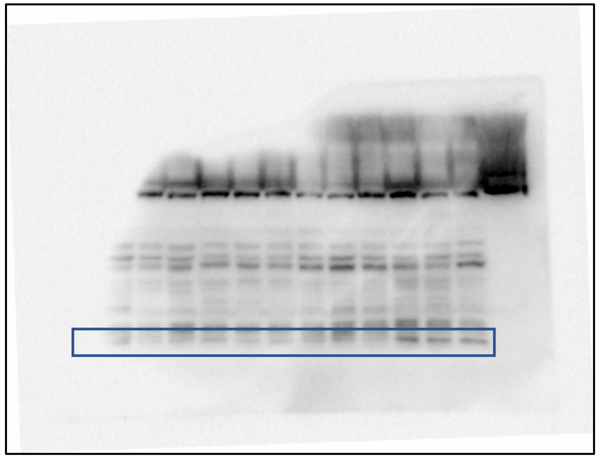

Anti-APOA1 N208

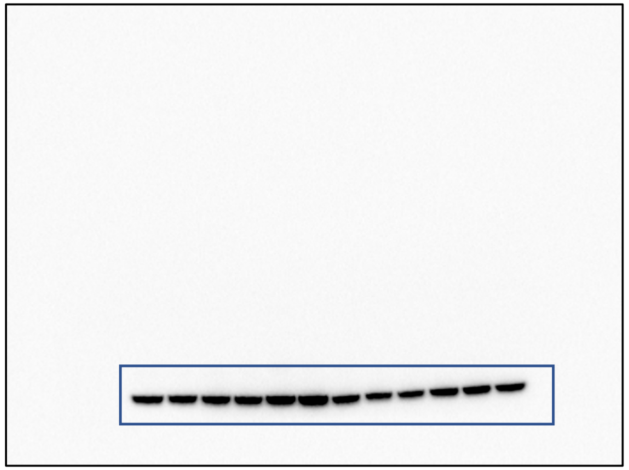

Anti-GAPDH

Full unedited blot/gel for Figure 4E

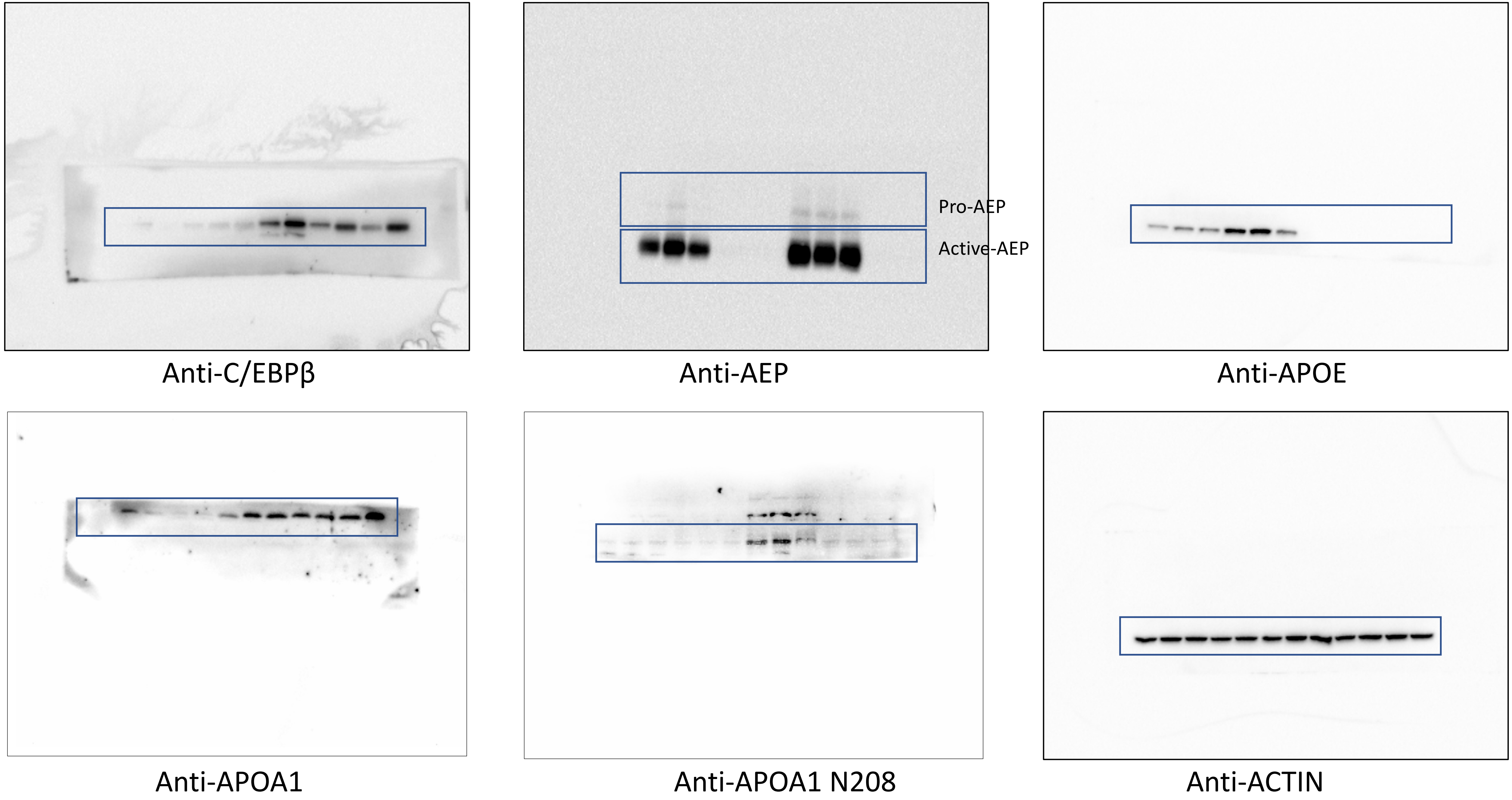

Full unedited blot/gel for Figure 5A

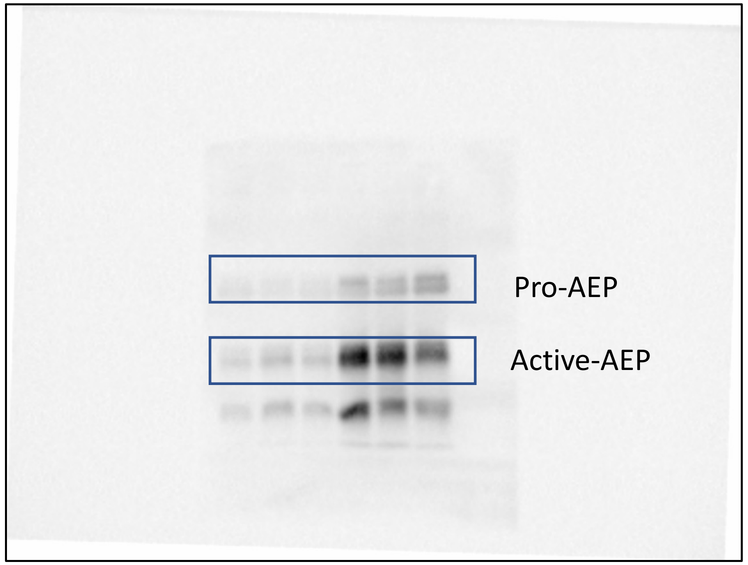

Anti-AEP

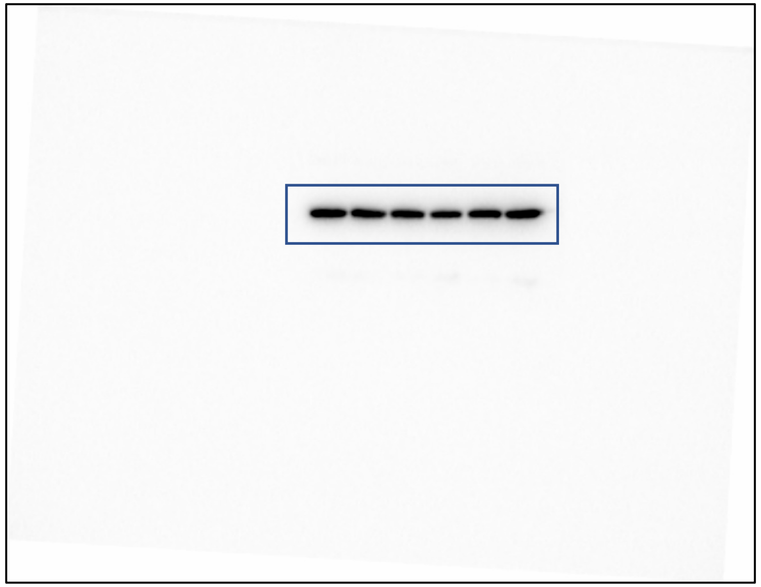

Anti-GAPDH

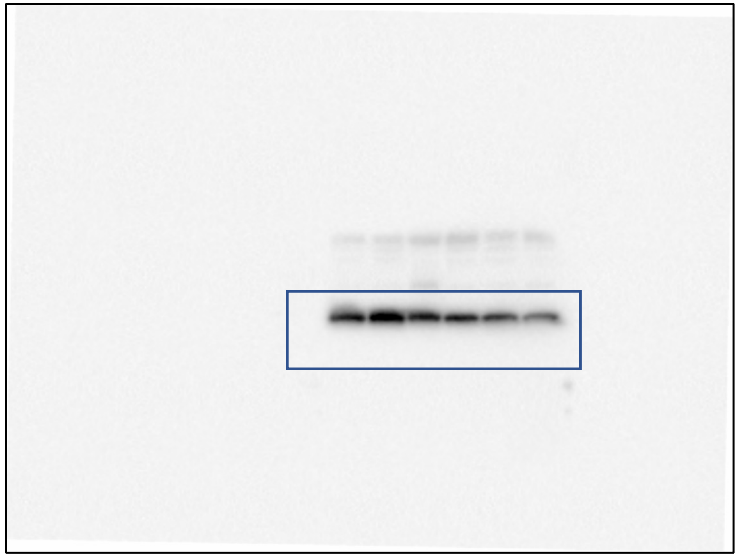

Anti-APOA1

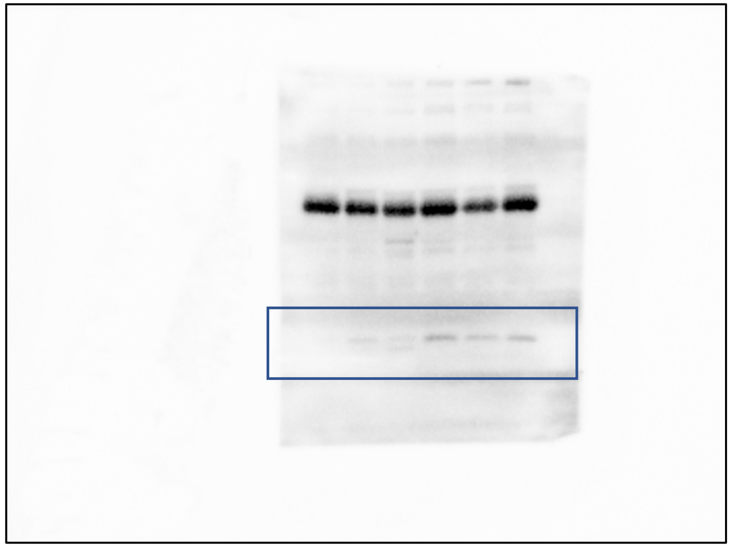

Anti-APOA1 N208

Full unedited blot/gel for Figure 5F

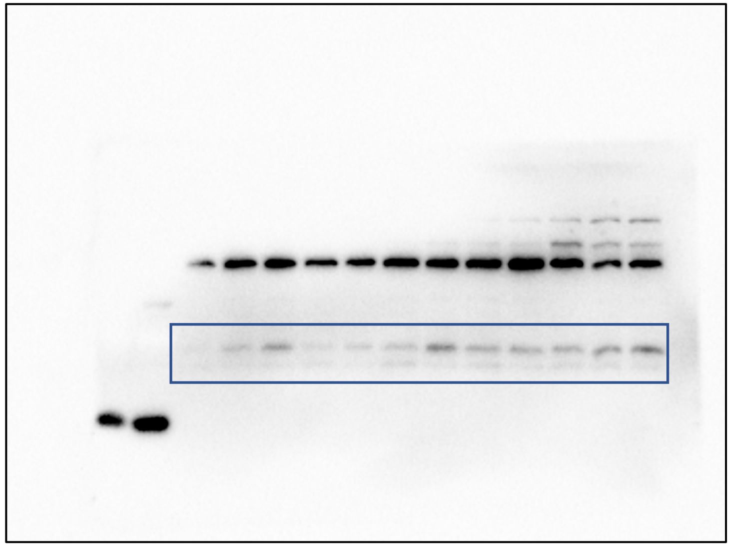

Anti-APOA1

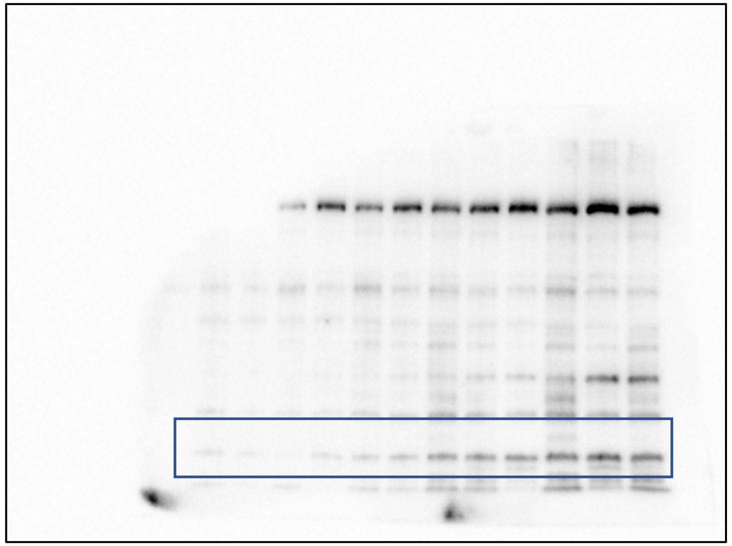

Anti-APOA1 N208

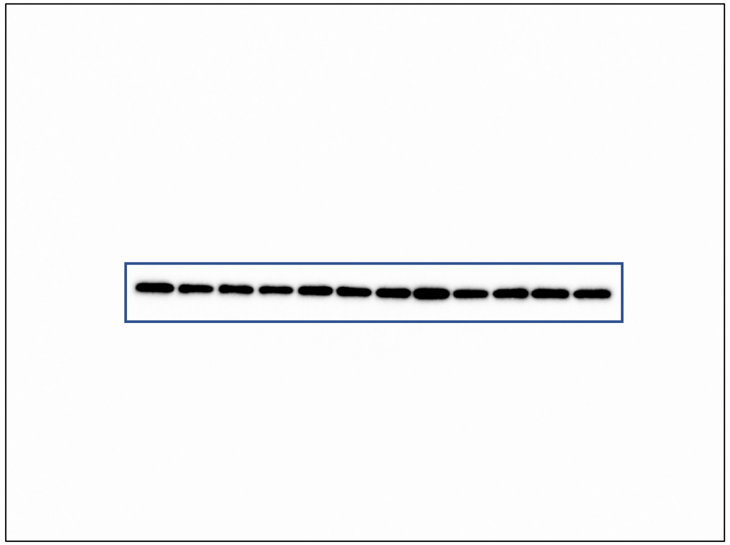

Anti-GAPDH

Full unedited blot/gel for Figure 5J

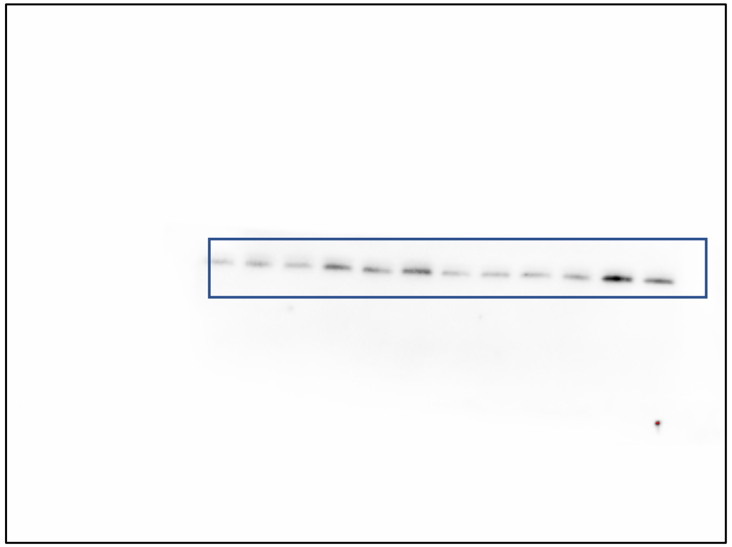

Anti-APOA1

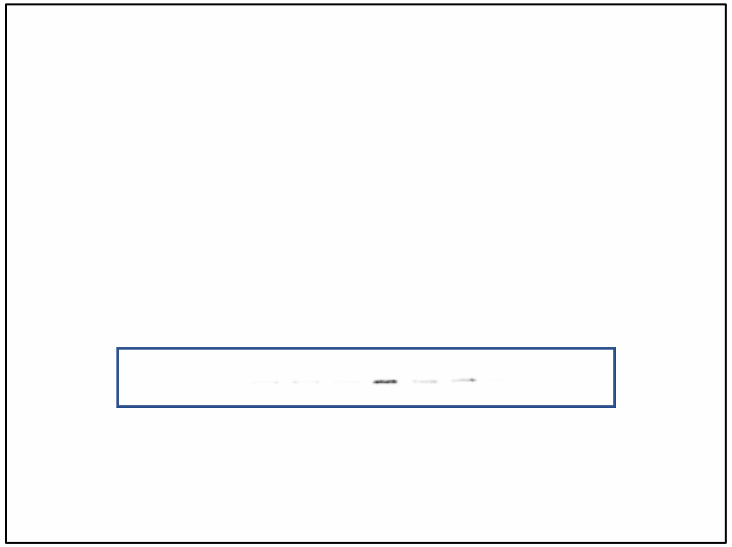

Anti-APOA1 N208

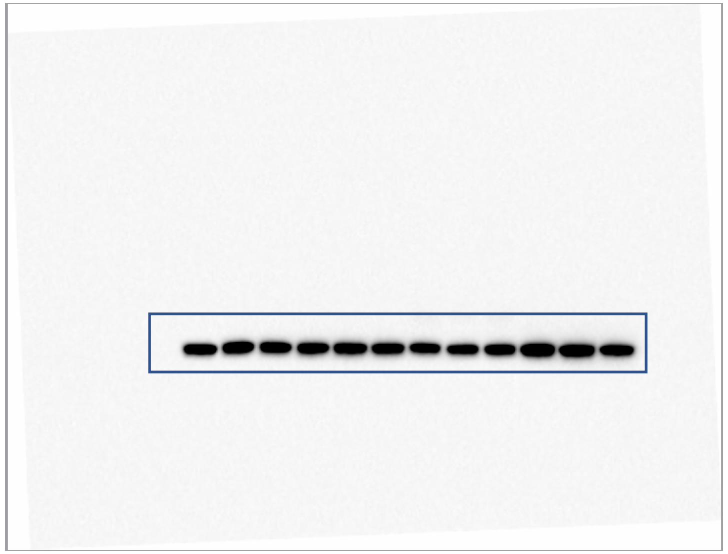

Anti-GAPDH

Full unedited blot/gel for Figure 6G

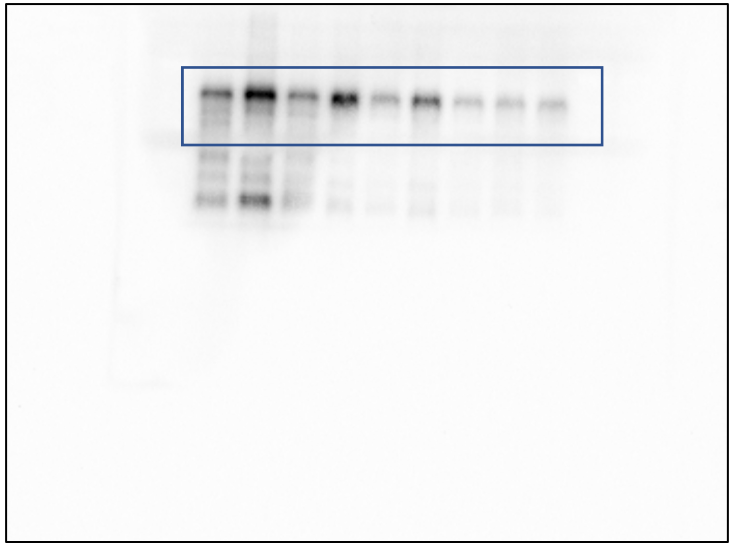

Anti-C/EBPβ

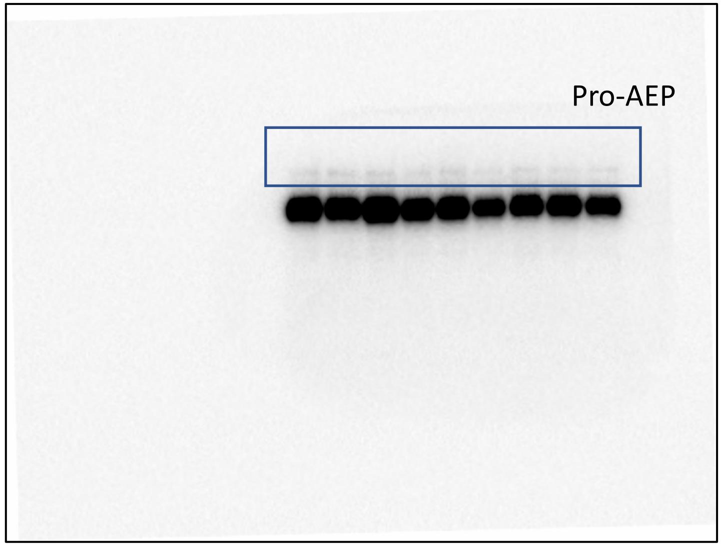

Anti-AEP (long-time exposure)

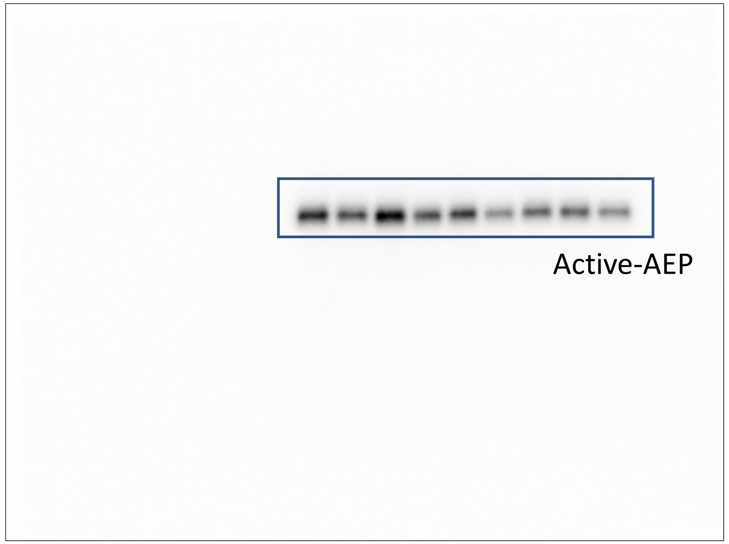

Anti-AEP (short-time exposure)

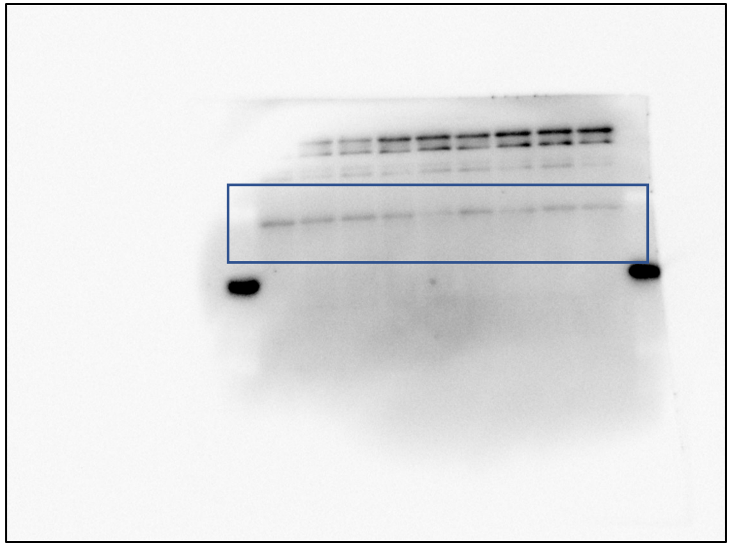

Anti-APOA1

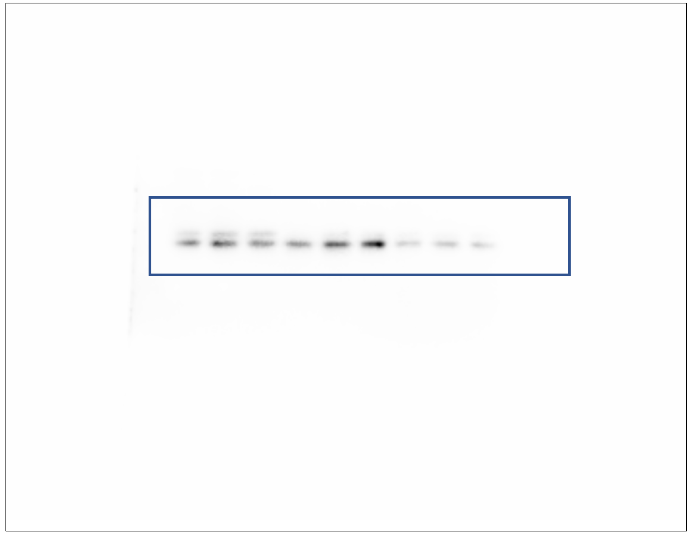

Anti-APOA1 N208

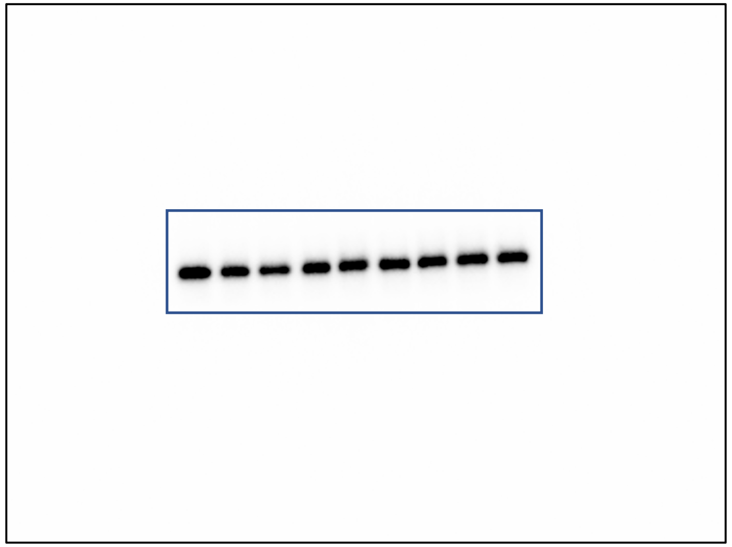

Anti-GAPDH

Full unedited blot/gel for Figure 6J

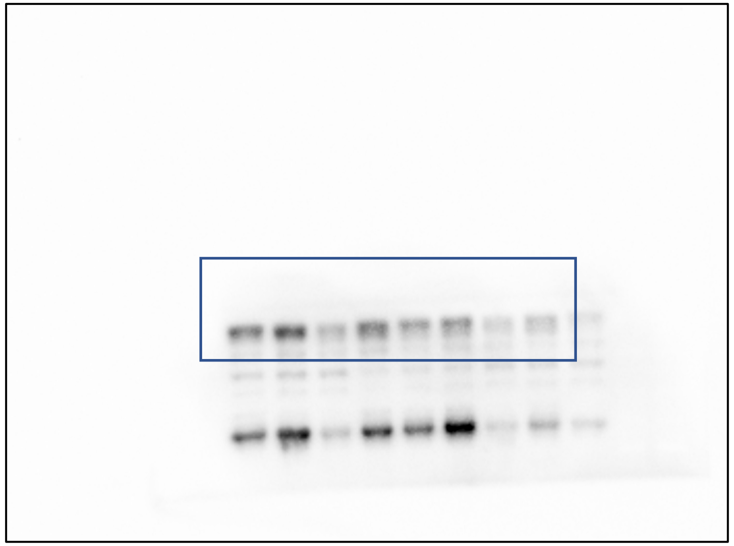

Anti-C/EBPβ

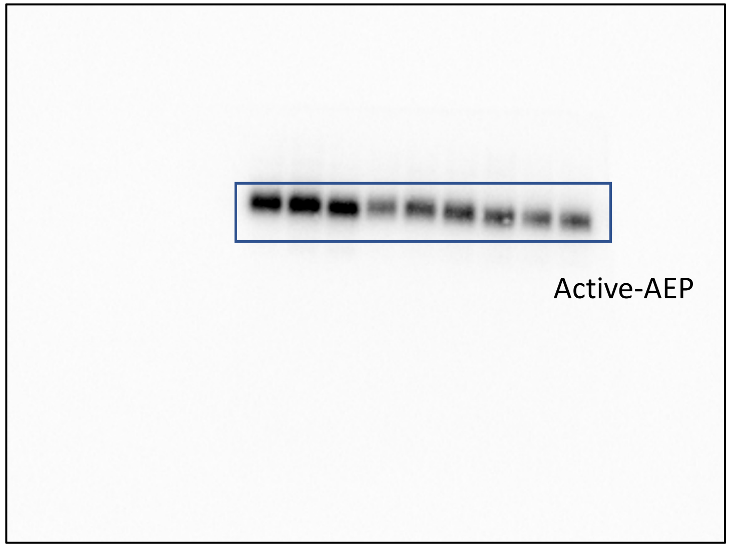

Anti-AEP

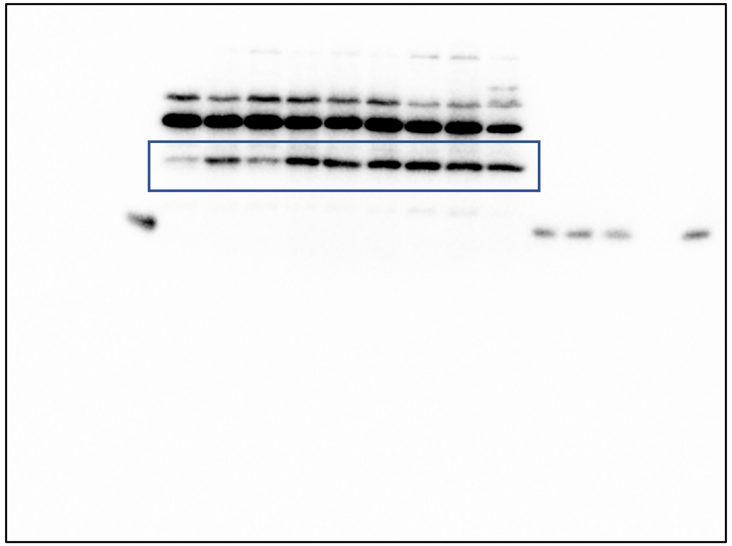

Anti-APOA1

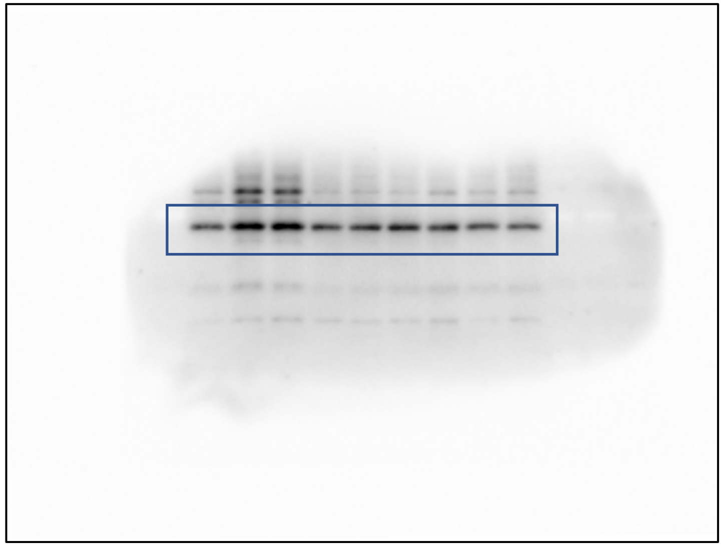

Anti-APOA1 N208

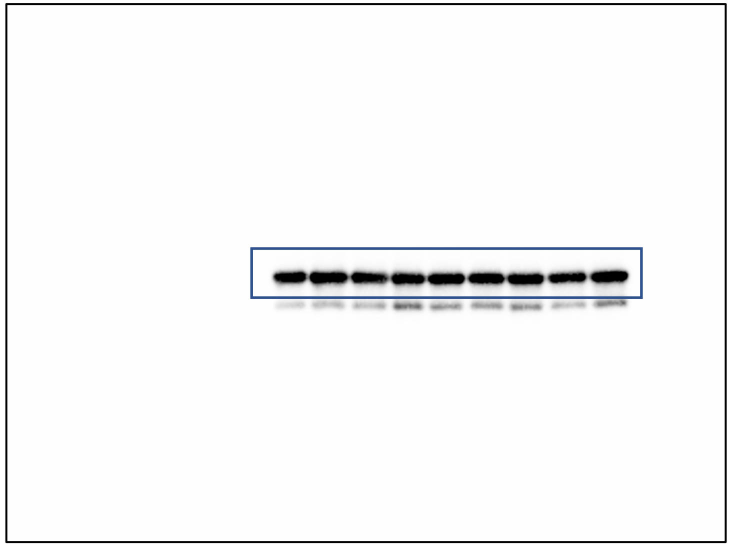

Anti-GAPDH

Full unedited blot/gel for Figure 7G

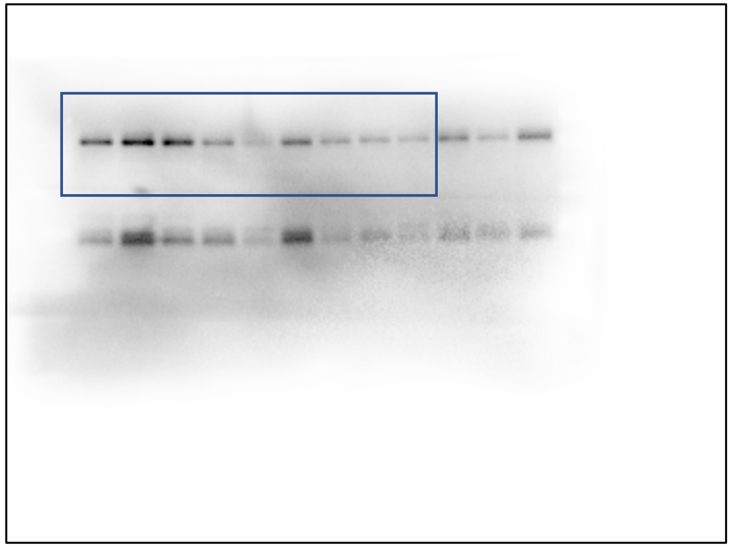

Anti-C/EBPβ

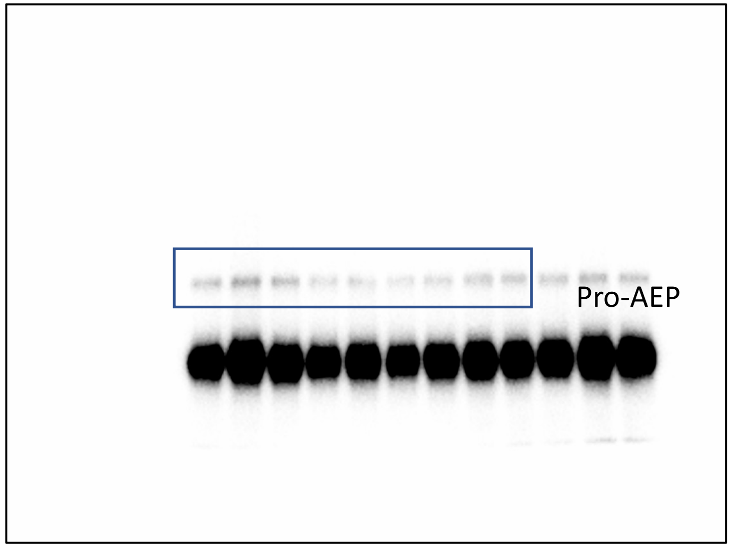

Anti-AEP (long-time exposure)

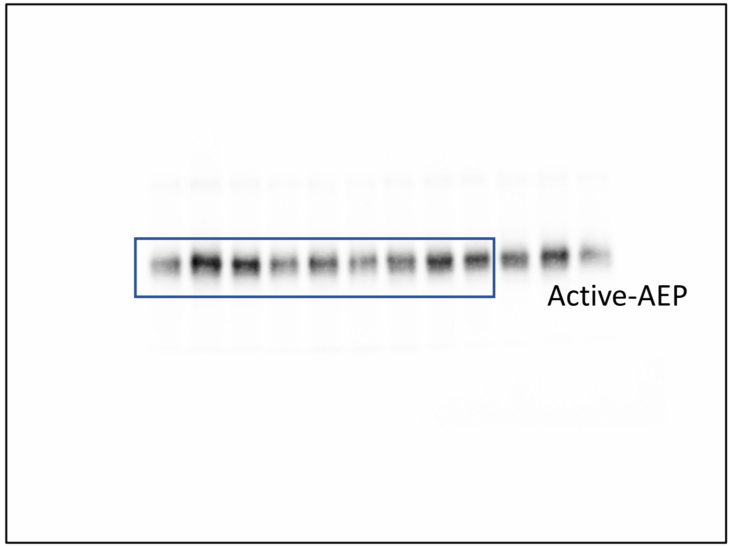

Anti-AEP (short-time exposure)

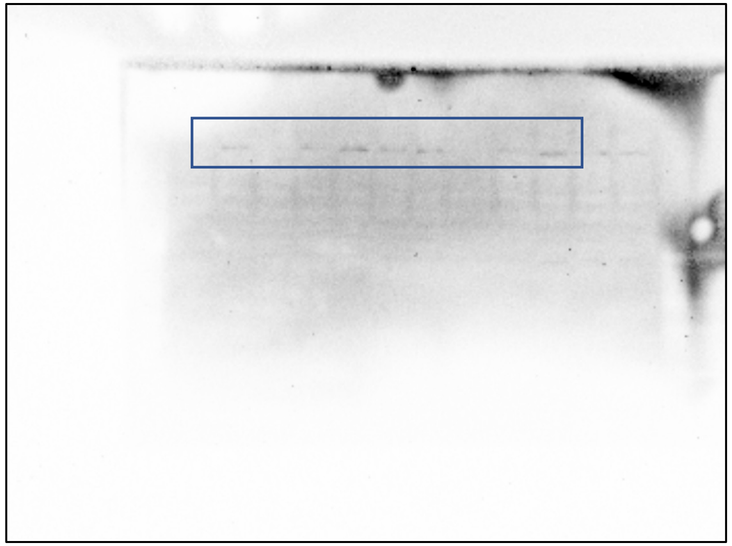

Anti-APOA1

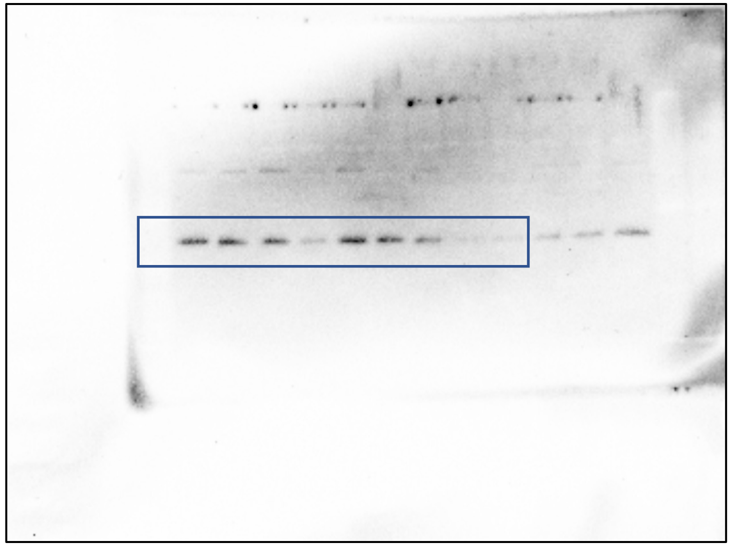

Anti-APOA1 N208

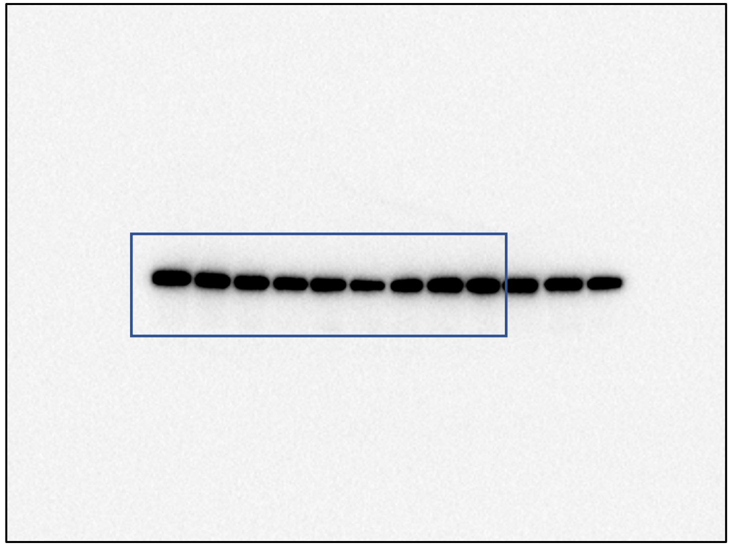

Anti-GAPDH

Full unedited blot/gel for Figure 7J

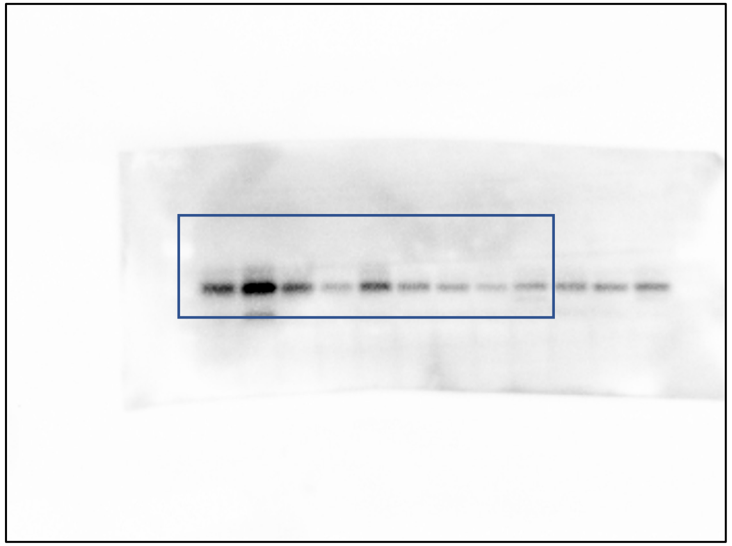

Anti-C/EBPβ

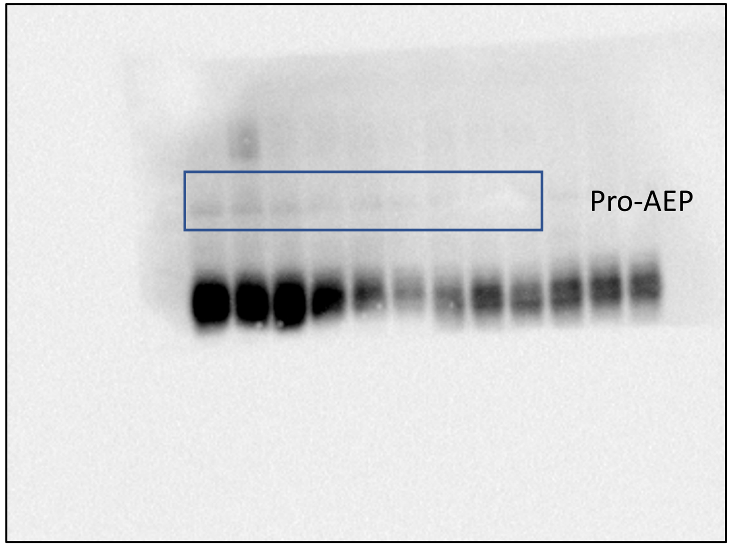

Anti-AEP (long-time exposure)

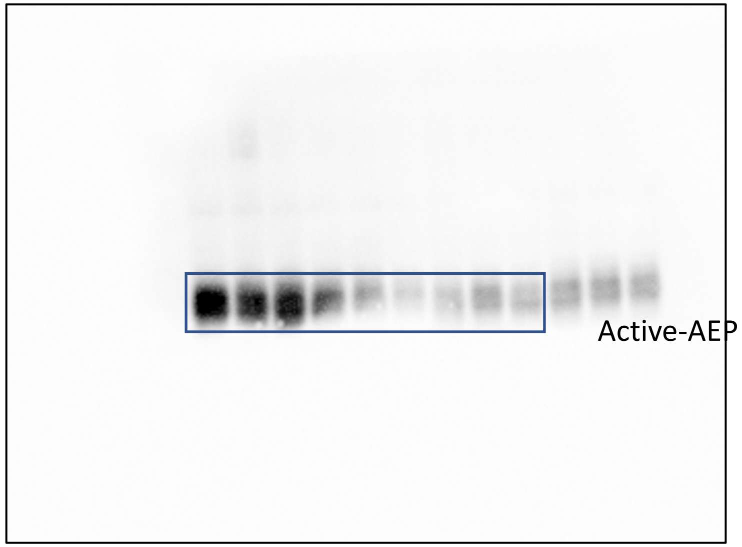

Anti-AEP (short-time exposure)

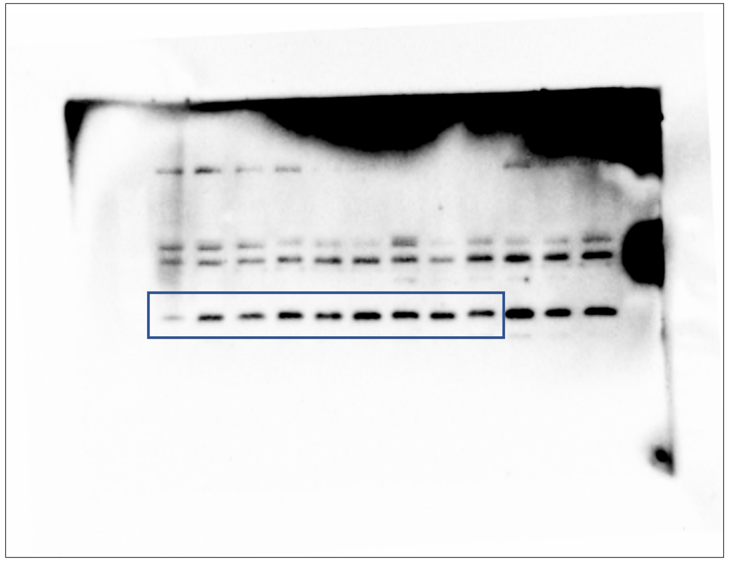

Anti-APOA1

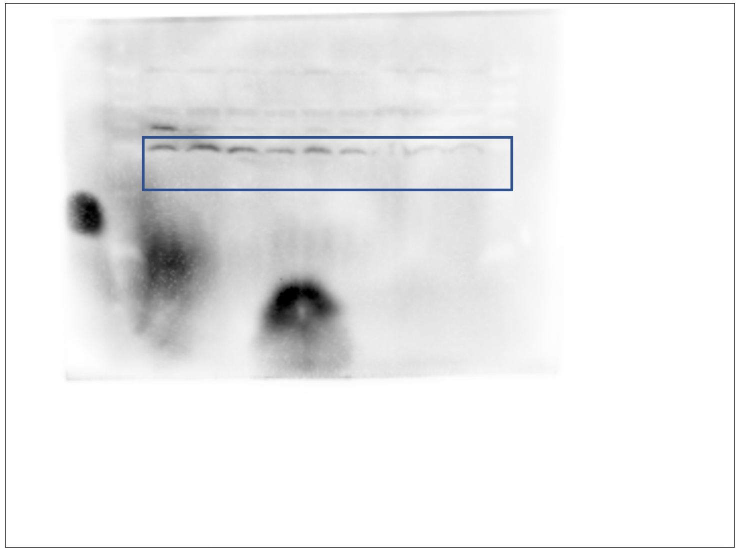

Anti-APOA1 N208

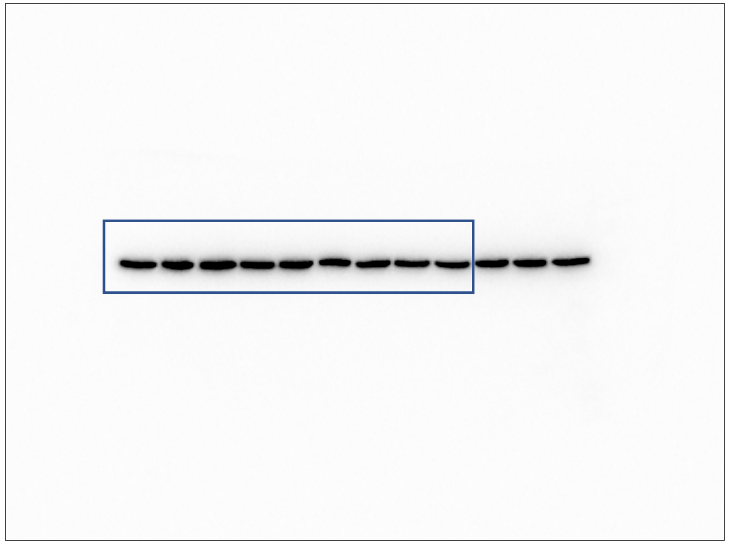

Anti-GAPDH

Full unedited blot/gel for Supplementary Figure 7G

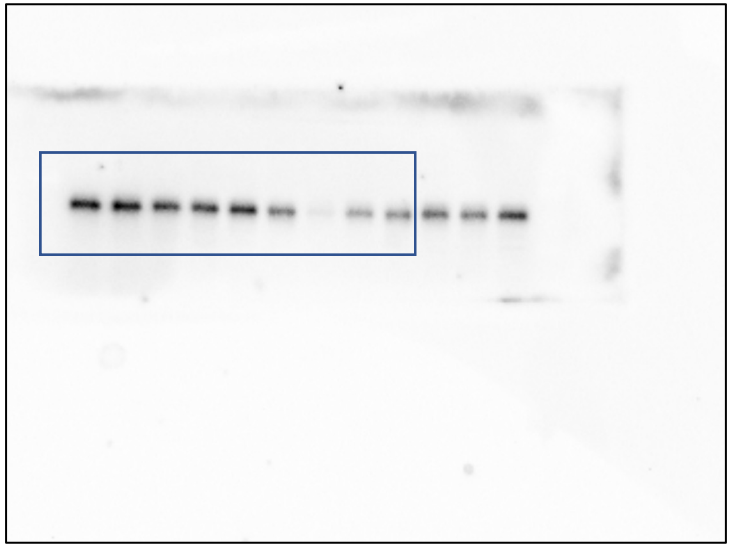

Anti-C/EBPβ

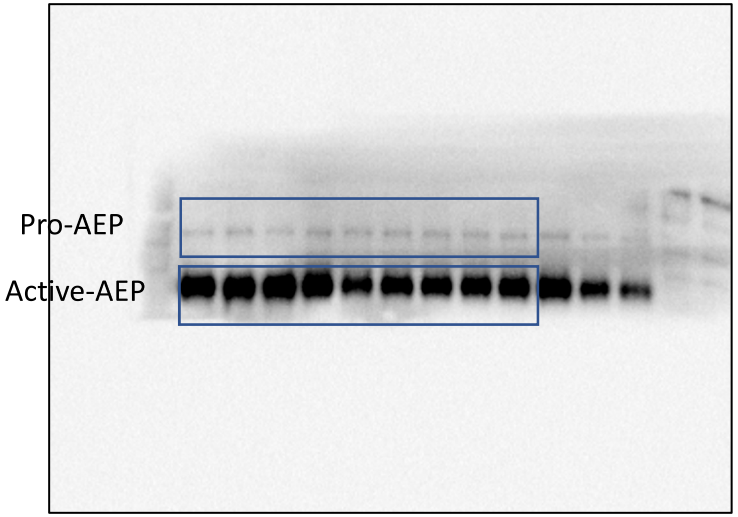

Anti-AEP

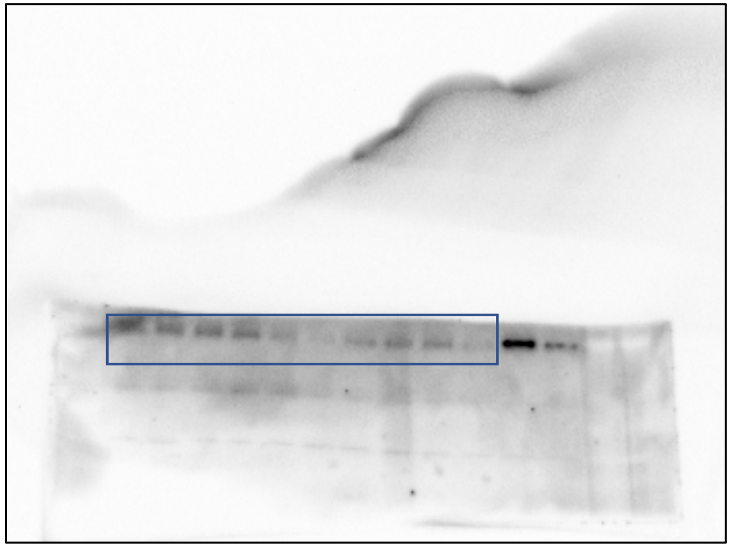

Anti-APOA1

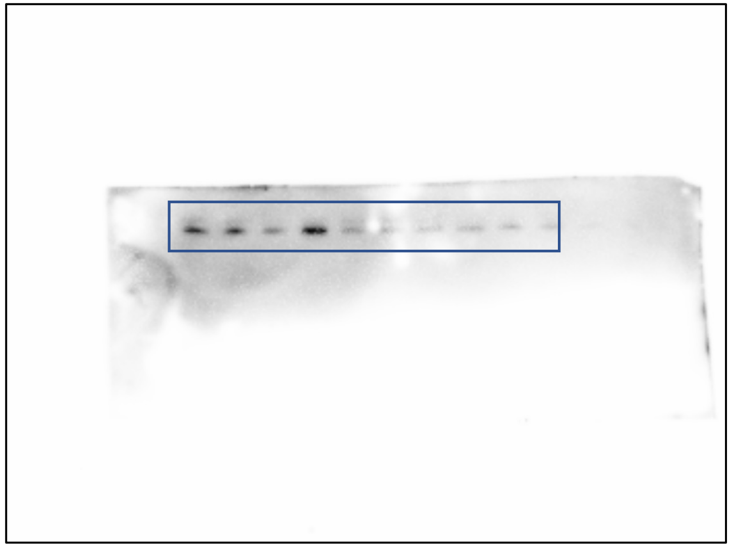

Anti-APOA1 N208

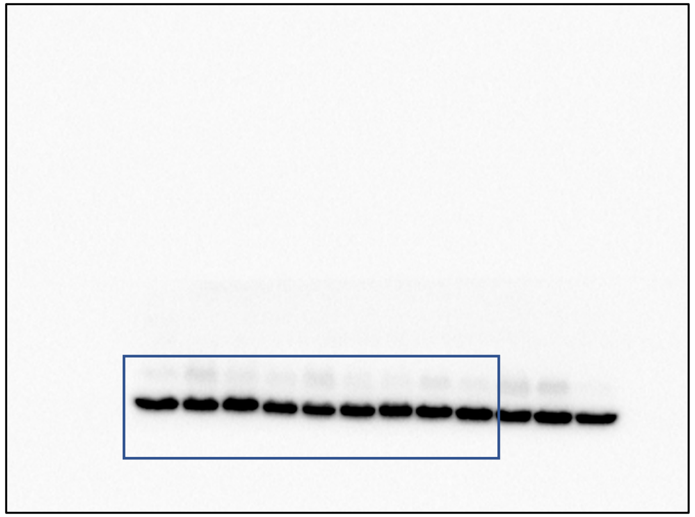

Anti-βACTIN

Full unedited blot/gel for Supplementary Figure 7J

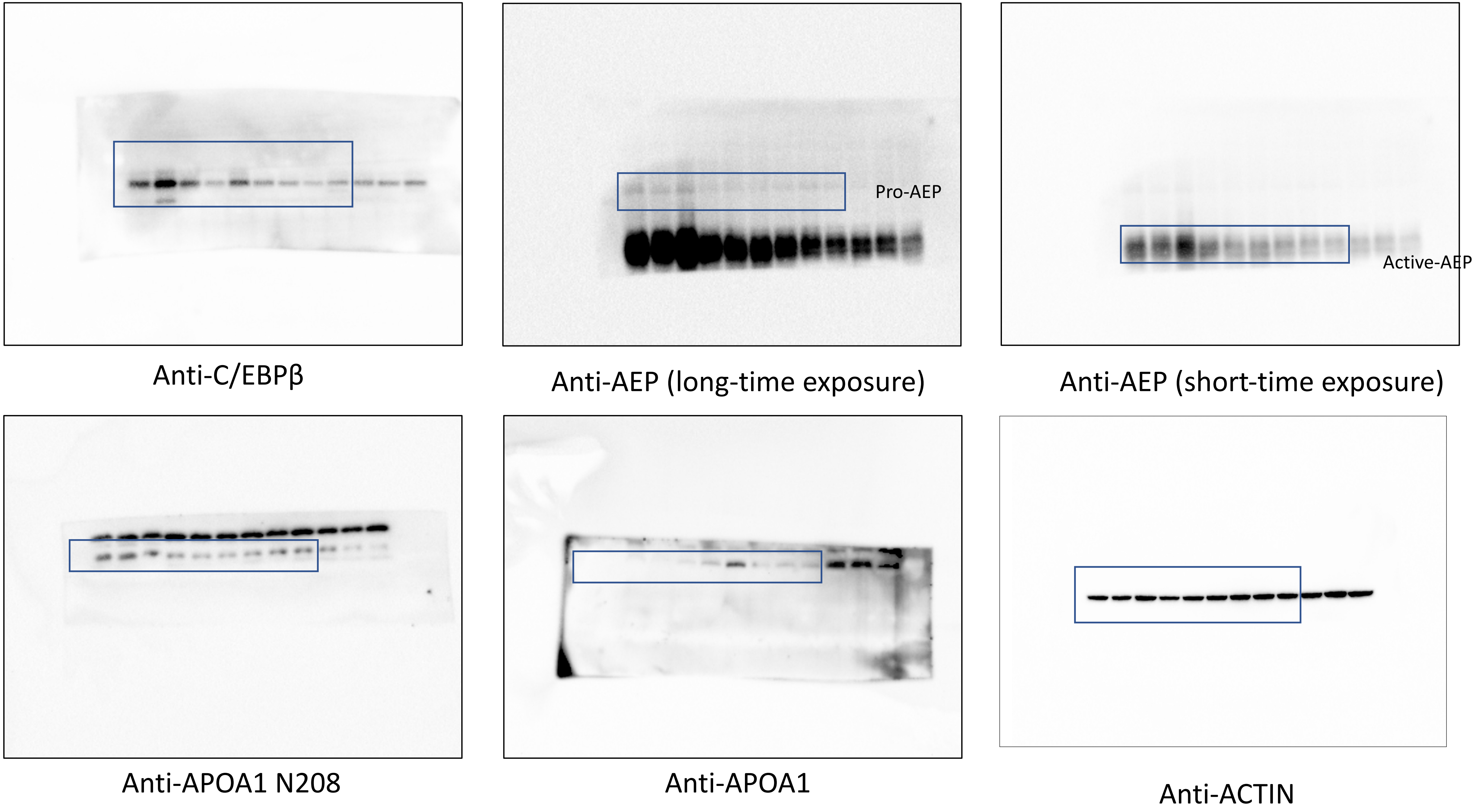

Supplement: Unedited blot and gel images [file jci-135-185128-s009.pdf]
